# Fluorinated Radicals in Divergent Synthesis via Photoredox Catalysis

**DOI:** 10.1021/acs.accounts.5c00239

**Published:** 2025-06-11

**Authors:** Rahul Giri, Anthony J. Fernandes, Dmitry Katayev

**Affiliations:** Department of Chemistry, Biochemistry and Pharmaceutical Sciences, 27210 University of Bern, Freiestrasse 3, 3012 Bern, Switzerland

## Abstract

Fluorine is an essential element in pharmaceuticals, agrochemicals, and material sciences, significantly enhancing the bioactivity, metabolic stability, and physicochemical properties of organic molecules. In medicinal chemistry, nearly 20% of marketed drugs contain at least one fluorine atom within their core structure. Despite its widespread importance, naturally occurring organofluorine compounds are exceedingly rare, necessitating the development of productive synthetic strategies for fluorine incorporation. The majority of fluorination protocols at the industrial level rely on reagents made from highly reactive and hazardous hydrogen fluoride (HF) or elemental fluorine (F_2_), which present substantial challenges in handling and safety at the laboratory scale. Moreover, considerations of cost, availability, and synthetic performance have led to a renewed interest in utilizing readily accessible, bulk-manufactured compounds such as fluorinated acids and anhydrides. The activation of these redox-active reagents presents a promising avenue to achieve selective, efficient, and sustainable fluoroalkylation reactions.

Over the past four decades, advancements in classical organic synthesis have given rise to new and transformative fields, enabling access to previously elusive chemical reactions. Among these, photoredox catalysis has emerged as a powerful tool, driving the evolution of synthetic organic chemistry through innovative concepts such as late-stage functionalization, atom economy, bifunctional reagents, switchable divergent synthesis, and multicomponent reactions. This account details our five-year journey in advancing radical fluoroalkyl chemistry through a detailed reactivity exploration of redox-active fluorinated acids and anhydrides. We also highlight the key concept of switchable divergent synthesis through photoredox catalysis as an elegant tool for facilitating molecular design. By carefully tuning reaction parameters, such as solvent, gas pressure, concentration, and additives, we achieve precise control over reaction intermediates, allowing for the selective generation of multiple fluorine-containing products from a common set of starting materials. This strategy not only improves synthetic efficiency but also broadens the chemical space accessible to fluorinated molecules, reducing costs and streamlining synthetic workflows. These photoredox methodologies have enabled the direct synthesis of a diverse range of fluorinated compounds, including trifluoromethylated ketones, γ-lactones, γ-lactams, esters, with high selectivity and remarkable functional group tolerance. Furthermore, the scalability and operational simplicity of these photoredox protocols make them attractive for broader applications, aligning with the goals of sustainable and cost-effective synthetic methods. Beyond synthetic applications, we have focused on elucidating the mechanistic aspects of these transformations. Through a combination of spectroscopic, experimental and computational studies, including a newly designed DLPNO–CCSD­(T)-based reactivity scale, we have gained valuable insights into the origins of divergence, radical reactivity, and a deeper understanding of the effects of radical polarity.

## Graphical abstract

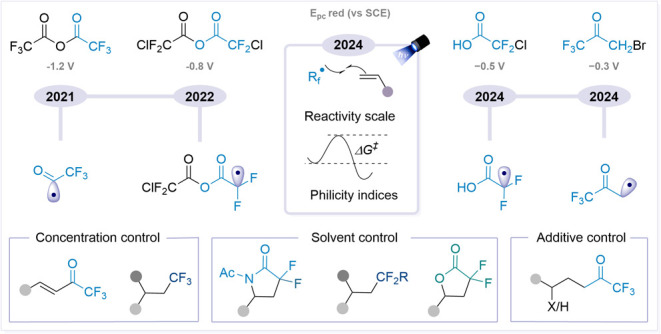

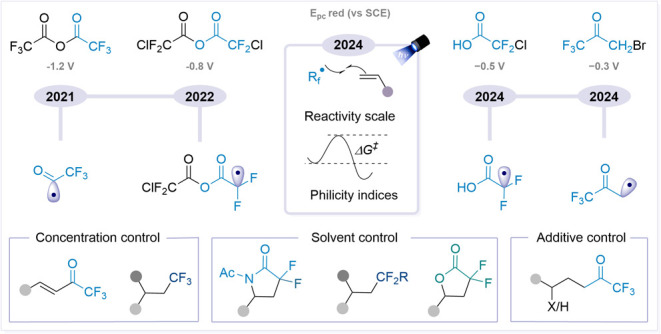

## Key references

-

Zhang, K. ; Rombach, D. ; Nötel, N. Y. ; Jeschke, G. ; Katayev, D. Radical Trifluoroacetylation of Alkenes Triggered by a Visible-Light-Promoted C-O Bond Fragmentation of Trifluoroacetic Anhydride. Angew. Chem., Int. Ed. 2021, 60­(41), 22487–22495 10.1002/anie.202109235 PMC851841334289531.^1^ *This work demonstrates the formation of one of the first trifluoroacetyl radical from trifluoroacetic anhydride (TFAA). Reaction with alkenes produces α,β-unsaturated trifluoromethyl ketones. Adjusting concentrations yields CF*_*3*_ *-adducts exclusively, and the* ^*•*^ *COCF*_*3*_ *radical can be stabilized under CO atmosphere*.
-

Giri, R. ; Mosiagin, I. ; Franzoni, I. ; Yannick Nötel, N. ; Patra, S. ; Katayev, D. Photoredox Activation of Anhydrides for the Solvent-Controlled Switchable Synthesis of gem-Difluoro Compounds. Angew. Chem., Int. Ed. 2022, 61, e202209143 10.1002/anie.202209143 PMC982652935997088.^2^ *In this work, we present a switchable protocol for synthesizing diverse gem-difluoro compounds using chlorodifluoroacetic anhydride (CDFAA) as a cost-effective fluoroalkylating reagent. By switching the solvent, up to three distinct fluorinated compounds can be obtained from the same starting materials*.
-

Giri, R. ; Zhilin, E. ; Katayev, D. Divergent Functionalization of Alkenes Enabled by Photoredox Activation of CDFA and α-Halo Carboxylic Acids. Chem. Sci. 2024, 15­(27), 10659–10667 10.1039/D4SC01084A 38994427 PMC11234866.^3^ *We utilized α-haloacids for the divergent functionalization of olefins, using solvents and additives to control reactivity, and showcased their effectiveness in the concise synthesis of natural products*.
-

Giri, R. ; Zhilin, E. ; Fernandes, A. J. ; Ordan, Q. E. L. ; Kissling, M. ; Katayev, D. Divergent Synthesis of Trifluoromethyl Ketones via Photoredox Activation of Halotrifluoroacetones. Helv. Chim. Acta 2024, 107, e202400125 10.1002/hlca.202400125 .^4^ *We employed α-halo ketones for the divergent synthesis of trifluoromethyl ketones by controlling reactivity through reaction intermediates*.
-

Fernandes, A. J. ; Giri, R. ; Houk, K. N. ; Katayev, D. Review and Theoretical Analysis of Fluorinated Radicals in Direct C_Ar_-H Functionalization of (Hetero)­Arenes. Angew. Chem., Int. Ed. 2024, 63, e202318377 10.1002/anie.202318377 38282182.^5^ *This work provides original computational analysis of key intrinsic parameters (e.g., philicity indices, redox potentials) and a reactivity scale to contextualize the reactivity of fluorinated radicals*.

## 1 Introduction

*“Very few people will use elemental fluorine other than at near-gunpoint, and some of the other classic reagents are still quite unfriendly, tending to leave cursing chemists swearing never to touch them again.”*

-By Derek Lowe^6^

Fluorine’s unique features, including high electronegativity, low polarizability, and small size, have a profound impact on the physical, chemical, and biological properties of molecules.^7,8^ As a result, fluorinated compounds are widely employed to enhance metabolic stability and lipophilicity,^7^ while also finding extensive applications in ^18^F imaging^9^ and advanced materials.^10^ Due to these properties, fluorine is an important component of pharmaceutical development and is often considered as *“magic element”*.^11^ A Reaxys database analysis shows that trifluoromethyl (R–CF_3_), where R can be any element, and difluoromethyl (R–CF_2_H) compounds are the most common fluorinated building blocks, owing to their synthetic versatility and commercial availability. In contrast, structures such as R–CBr_2_F or R–CI_2_F are much less common due to synthetic difficulties or instability. Fluorine’s broader impact is evident by its presence in over 20% of pharmaceuticals and 30% of agrochemicals (Figure 1A).^12–14^ However, due to the strength of the C–F bond, these compounds are highly resistant to degradation, which also contributes to their toxicity and environmental persistence.

**Figure 1:**
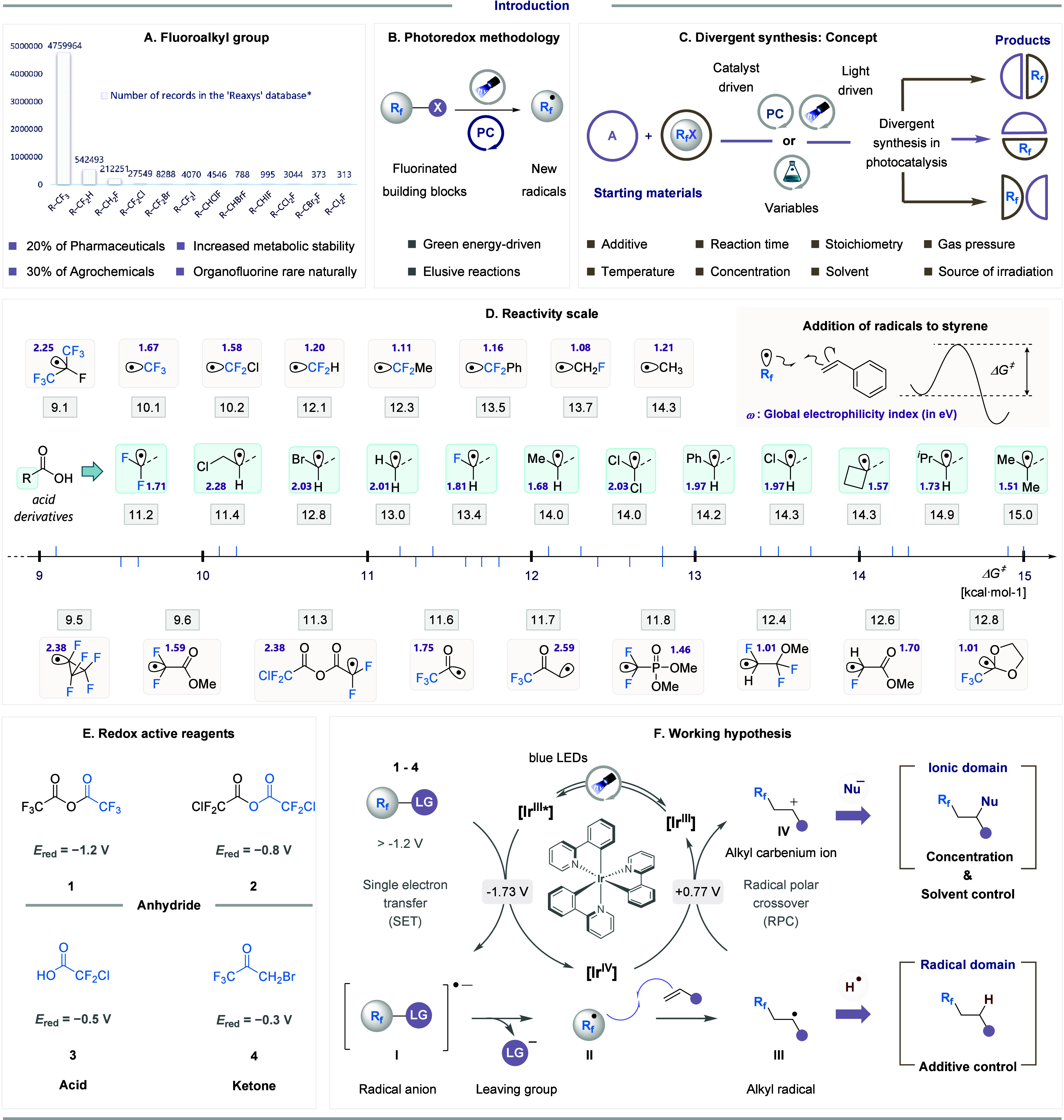
**A.** Significance of fluorine and related availability of the chemical space in Reaxys database; [*] Total available compounds were assessed using a Reaxys search reporting known compounds (on May 08, 2025). Duplicate records were not removed, and some values may include repetitions. **B.** Activation of redox active reagents using photoredoxchemistry. **C.** Concept of divergent synthesis. **D.** Reactivity scale in radical addition to olefins, computed at the DLPNO-(U)­CCSD­(T)/aug-cc-pVTZ,SMD­(MeCN)//(U)­M062X-D3/def2-TZVP,SMD­(MeCN)) level of theory. **E.** Redox-active species examined in this account (−1.2 to 0 V (vs SCE) for reagents 1–4). **F.** Underlying mechanistic framework.

Since naturally occurring organofluorine compounds are rare,^15^ recent efforts have focused on developing selective, safe, and controlled fluorination methods to improve their accessibility for academic research and industrial applications.^5,16,17^ Conventional fluorination often relies on harsh reagents, leading to low selectivity and significant handling challenges,^18^ although recent breakthroughs by the Gouverneur group are reshaping the field.^19–21^ To circumvent these challenges, researchers have developed bench-stable fluorinated functional group transfer reagents (FGTRs) as safer and more attractive alternatives.^22–25^ Despite their efficiency and versatility, FGTRs are limited by step-intensive preparation. To meet the growing demand in organofluorine chemistry, the focus has shifted toward commercially viable feedstocks (including anhydrides, acids, etc.) to facilitate previously inaccessible transformations while ensuring atom economy and cost-effective production.^26–28^ The activation of these laboratory commodities using green methodologies, such as photoredox catalysis, has proven beneficial for sustainable transformations,^29^ generating reactive radicals under mild conditions to facilitate selective, safe, and controlled fluoroalkylation reactions (Figure 1B).

Radical reactions are decisive for progress in chemical synthesis as they enable transformations that are difficult or inaccessible with traditional two-electron ionic mechanisms.^30^ For example, paradigms such as hydrogen atom transfer (HAT),^31^ radical addition to alkenes, and radical-polar crossover (RPC)^32^ have opened up new horizons in the selective functionalization of unsaturated hydrocarbons, which are of utmost importance in synthetic organic chemistry. A crucial realization in this context is that controlling reaction intermediates in radical processes allows for selective modulation of product outcomes. By leveraging this principle, precise adjustments of reaction conditions such as solvent, concentration, additives, temperature, light, and catalyst can steer the reaction pathway, leading to the production of a variety of different fluorinated compounds from the same starting materials (Figure 1C). Such an approach, coined switchable divergent synthesis, is of great value as it takes full advantage of the polyvalent reactivity of key radical intermediates, minimizing the cost and effort to expand the chemical space and making it an attractive tool for the synthesis of multiple products through simple adjustment of reaction parameters.^33^

Over the past five years, we have focused our efforts on developing new methodologies for the direct activation of commercially viable, low-cost sources as redox active reagents, including trifluoroacetic anhydride (TFAA),^1^ chlorodifluoroacetic anhydride (CDFAA),^2^ α-halocarboxylic acids,^3^ and α-halotrifluoroacetones^4^ to generate fluorinated radicals. These radicals facilitate fluoroalkyl incorporation onto alkenes, constructing C–C bonds while simultaneously forming C–O, C–N, and C–H bonds. In some instances, they act as bifunctional reagents, enhancing atom economy, which is crucial for the synthesis of complex organic molecules and late-stage diversification. We have showcased that these switchable divergent reactions are scalable and demonstrate excellent chemo- and regioselectivity with the applications in concise synthesis of natural products. Extensive studies combining computational modeling, spectroscopy, and experimental analysis provide deep insights into the reaction mechanism and reactivity of fluorinated radicals.

Given the significance of this field, applied theoretical chemistry provides a rational understanding of the reactivity and nature of radicals, and it is an essential component in enhancing the chemical intuition of experimental chemists.^34,35^ In this context, the electrophilicity concept introduced by Parr et al. has proven particularly useful in the description and prediction of radical reactivity (e.g., rate enhancement through polarity match).^36–39^ We have been involved in computational studies^5^ to better understand the reactivity of fluorinated radicals^40,41^ through calculations of their philicity parameters (electrophilicity and nucleophilicity indices) based on De Proft’s method.^42^ Shortly thereafter, this concept was extended to various commonly encountered radicals.^43^ While the electrophilicity parameter is a useful and straightforward approach to predict chemical reactivity, it remains a relatively simplistic descriptor that can overlook key mechanistic complexities. In contrast, the calculation of the reaction barrier provides a more accurate perspective. Therefore, we elaborated a reactivity scale based on high-accuracy DLPNO–CCSD­(T) calculations^44^ of reaction barriers for the addition of fluorinated radicals to benzene, providing a complete picture of their overall reactivity.^45,46^

In this account, we take the opportunity to unify our efforts in switchable divergent synthesis and quantify the reactivity of fluorinated radicals involved through the elaboration of a reactivity scale. We have calculated Gibbs-free energy barriers for the addition of various radicals—including those explored experimentally by our group and key radicals as reference—to styrene and compiled the results in Figure 1D, alongside their electrophilicity (ω) (see SI for computational details). As observed in our previous study on radical addition to benzene^5^ and consistent with Dolbier’s report,^41^ highly fluorinated species rank among the most reactive of the series. The activation energies span approximately 6 kcal·mol^–1^, ranging from 9.1 kcal·mol^–1^ for ^•^CF­(CF_3_) radical to 15 kcal·mol^–1^ for the ^•^CMe_2_CO_2_H radical. The reactivity window of carboxyl-substituted radicals is relatively broad, highlighting the notable influence of the substitution on reactivity. Among these, ^•^CF_2_CO_2_H is the most reactive toward styrene (11.2 kcal·mol^–1^). The reactivity trend with respect to fluorine substitution in these acids follows ^•^CF_2_CO_2_H > ^•^CH_2_CO_2_H > ^•^CHFCO_2_H (11.2, 13.0, 13.4 kcal·mol^–1^, respectively). The −CO_2_H moiety has a pronounced effect on radicals electrophilicity, increasing it by approximately 0.5 eV compared to a hydrogen-substituted analogue ω­(^•^CF_2_H) = 1.20 *versus ω*(^•^CF_2_CO_2_H) = 1.71). The impact of deprotonation of the −CO_2_H moiety on philicity was also assessed for ^•^CF_2_CO_2_M, ^•^CHF_2_CO_2_M, and ^•^CH_2_CO_2_M radicals (M = Li, Na, and K). A decrease in electrophilicity of 0.3–0.5 eV was observed for the Li salts, while a more pronounced drop of 0.6–0.9 eV occurred for the K salts, consistent with the weaker coordination of the larger K^+^ ion with the negatively charged −CO_2_^–^ group (see SI). Interestingly, electrophilicity decreases with increasing fluorination in the ^•^Rf-CO_2_H series: ω­(^•^CH_2_CO_2_H) = 2.01 > ω­(^•^CHFCO_2_H) = 1.81 > ω­(^•^CF_2_CO_2_H) = 1.71. In contrast, the electrophilicity of bromo- and chloro-substituted radicals (^•^CHBrCO_2_H, ^•^CHClCO_2_H and ^•^CCl_2_CO_2_H) remains unchanged (ω = 1.97–2.03), closely resembling ^•^CH_2_CO_2_H. This suggests a significant stabilization of fluorinated radicals via *n*_F_→*p*_SOMO_ bonding interactions (see SI), a stabilization effect that does not translate efficiently to larger halogens due to their more diffuse orbitals.^47^ As expected, steric effects also influence radical behavior, with bulkier substituents leading to reduced reactivity (CH­(^*i*^Pr)­CO_2_H 14.9 and CMe_2_CO_2_H 15.0 kcal·mol^–1^). Finally, a direct correlation between computed reaction barriers and electrophilicity values yielded no meaningful results (r^2^ = 0.09). However, applying a modified Roberts-Steel relationship^48,49^ —similar to our previous work,^5^ incorporating radical electrophilicity—resulted in a significantly improved correlation (r^2^ = 0.79, see SI). This underscores the importance of electrophilicity in the description of radicals’ reactivity, although the electrophilicity value alone is insufficient, particularly for structurally diverse radicals.

An outline of the mechanism of this process is illustrated in Figure 1F. Our approach relied on photoredox catalysis, specifically leveraging the redox properties of tris­(2-phenylpyridine) iridium­(III) *fac*-[Ir­(ppy)_3_]. Upon excitation by visible light at 440 nm—wavelengths where most organic molecules do not absorb—*fac*-[Ir­(ppy)_3_] undergoes single-electron transfer (SET) with anhydride, acid, or ketone reagents (**1**–**4**, Figure 1E). Radical **I** forms via outer-sphere electron transfer, enabled by the strongly reducing excited-state photocatalyst (*E*_1/2_ [Ir^IV^/Ir^III^*] = – 1.73 V vs SCE) compared to the reagents (*E*_*red*_ = – 1.2 to 0 V vs SCE).^50^ Radical intermediate **I** undergoes a mesolytic cleavage to yield the corresponding radical **II**, which adds to the olefin forming alkyl radical **III**. From **III**, the reaction outcomes can diverge either through direct trapping by HAT reagents to afford hydro-functionalization (radical domain), or subsequent SET oxidation by Ir^IV^ (*E*_1/2_ [Ir^IV^/Ir^III^] = +0.77 V vs SCE) to form carbenium ion **IV** (ionic domain) and regenerates the photocatalyst via a RPC pathway. Afterward, solvent and concentration adjustments allow control over the reactivity of species **IV**, facilitating difunctionalization through intra- or intermolecular nucleophilic attack.

We now revisit the reactions and reactivity investigated in our previous studies, with particular emphasis on the concept of divergence and the fundamental mechanisms that drive these transformations.

## 2 Activation of Trifluoroacetic Anhydride

Our journey on fluorinated radicals started with inspiration from prior photocatalytic decarboxylative methodologies, such as those reported by Stephenson and Sodeoka, who demonstrated the generation of fluoroalkyl radicals from perfluoroalkyl anhydrides using an external oxidant (e.g., pyridine *N*-oxide or urea·H_2_O_2_)^51,52^ We hypothesized that trifluoroacetic anhydride (TFAA, Figure 2A), a cost-effective and widely available reagent, could be directly activated under photoredox conditions and serve as a precursor for trifluoroacetyl radicals (Figure 2B).

**Figure 2:**
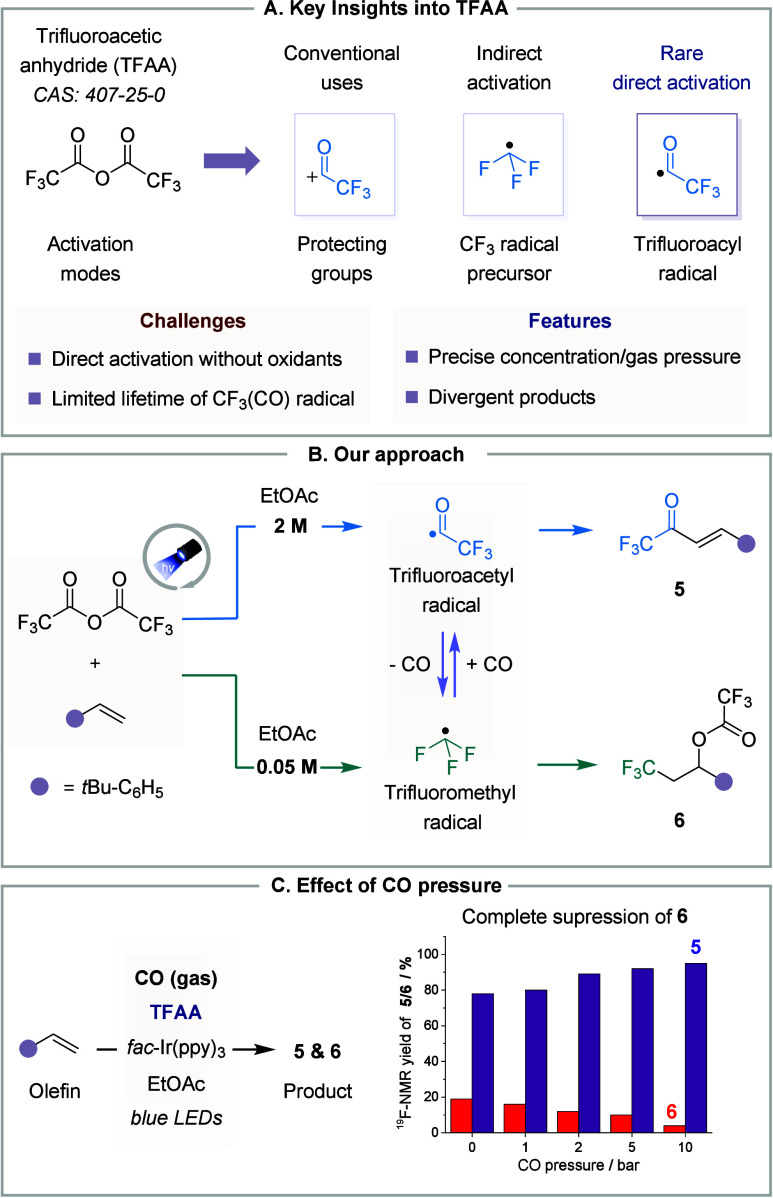
TFAA in divergent synthesis. **A.** Key features. **B.** Approach to divergent synthesis. **C.** Effect of CO pressure on product ratio.

Cyclic voltammetry analysis revealed that TFAA exhibits a reduction onset at approximately – 1.2 V (vs SCE), suggesting its susceptibility to a SET reduction when paired with a suitable photocatalyst. We anticipated that this reductive event would trigger an irreversible C–O bond fragmentation, forming a transient electrophilic ^•^COCF_3_ radical (ω = 1.75 eV) and a trifluoroacetate anion. Theoretical calculations supported this hypothesis, indicating an exergonic process (*ΔG* = – 4.8 kcal·mol^–1^) favoring the formation of the trifluoroacetyl radical over alternative pathways.

To evaluate this trifluoroacetylation strategy, Ir­(ppy)_3_ was chosen as the photocatalyst to reduce TFAA and promote the proposed SET. In the presence of an olefin, this approach efficiently delivered the trifluoroacetylated product **5** in an impressive 79% yield, demonstrating excellent chemo- and regioselectivity. Concentration adjustments further refined the protocol; reducing the substrate concentration to 0.05 M exclusively produced the trifluoromethylated adduct **6** in 85% yield, suggesting that lower concentration favors decarbonylation. This equilibrium between the trifluoroacetyl and trifluoromethyl radicals was further corroborated by high-pressure experiments, where increasing CO pressure (1–10 bar) suppressed trifluoromethylation by forcing the equilibrium toward ^•^COCF_3_ radical, resulting in nearly exclusive formation of **5** (Figure 2C). To further elucidate the competition between the two pathways, we have now calculated the reaction barrier for the decarbonylation using the same computational method as in the reactivity scale (Figure 1D) to enable reactivity comparison (see SI). The barrier for decarbonylation is found to be 12.0 kcal·mol^–1^ in acetonitrile (11.8 kcal·mol^–1^ in EtOAc), which is 0.4 kcal·mol^–1^ higher than the barrier for radical addition of ^•^COCF_3_ to styrene (11.4 kcal·mol^–1^ in EtOAc). These results indicate that radical addition is favored over decarbonylation in the case of styrene, yet the small energetic difference allows one to efficiently steer the reaction pathway by varying the reaction concentration. With optimized conditions established, we evaluated the substrate scope across a diverse array of alkenes (Figure 3). Aryl-substituted olefins bearing electron-donating and electron-neutral groups at various positions underwent trifluoroacetylation in moderate to excellent isolated yields. Scaling the reaction to 15.6 mmol with 4-*tert*-butylstyrene maintained a 67% yield, affirming its preparative potential. Notably, trifluoromethylated products became predominant when low concentrations (**6**, **14**) or electron-deficient olefins were used (**15**), resulting from Giese addition in the latter case.^53^ This is consistent with a slower radical addition due to the polarity mismatch between the electrophilic ^•^COCF_3_ radical (ω = 1.75 eV) and electron-deficient alkenes, collaterally favoring decarbonylation. Remarkably, functional groups such as halogens, esters, amides, and acetal remained untouched under the reaction conditions (**7**-**10**), offering handles for subsequent synthetic elaboration. The reaction also proved to be compatible with substrates bearing α-substituents and heterocycles, highlighting the method’s versatility. The protocol’s utility can be extended to pharmacologically relevant scaffolds. Key precursors to anti-inflammatory drugs, such as those bearing trifluoromethylated pyrazole moieties (e.g., Mavacoxib analog).^54^ Structurally intricate substrates like cholesterol (**12**) and nitogenin (**13**) exhibited exclusive functionalization at the less-hindered olefin, affording the corresponding CF_3_-enones. Finally, derivatization of these latter enabled the synthesis of trifluoromethylated heterocycles (*S*-, *O*-, *N*-containing, varying ring sizes), challenging to access via conventional routes (**16**-**18**). These results collectively demonstrate the method’s robustness and relevance to medicinal chemistry.

**Figure 3:**
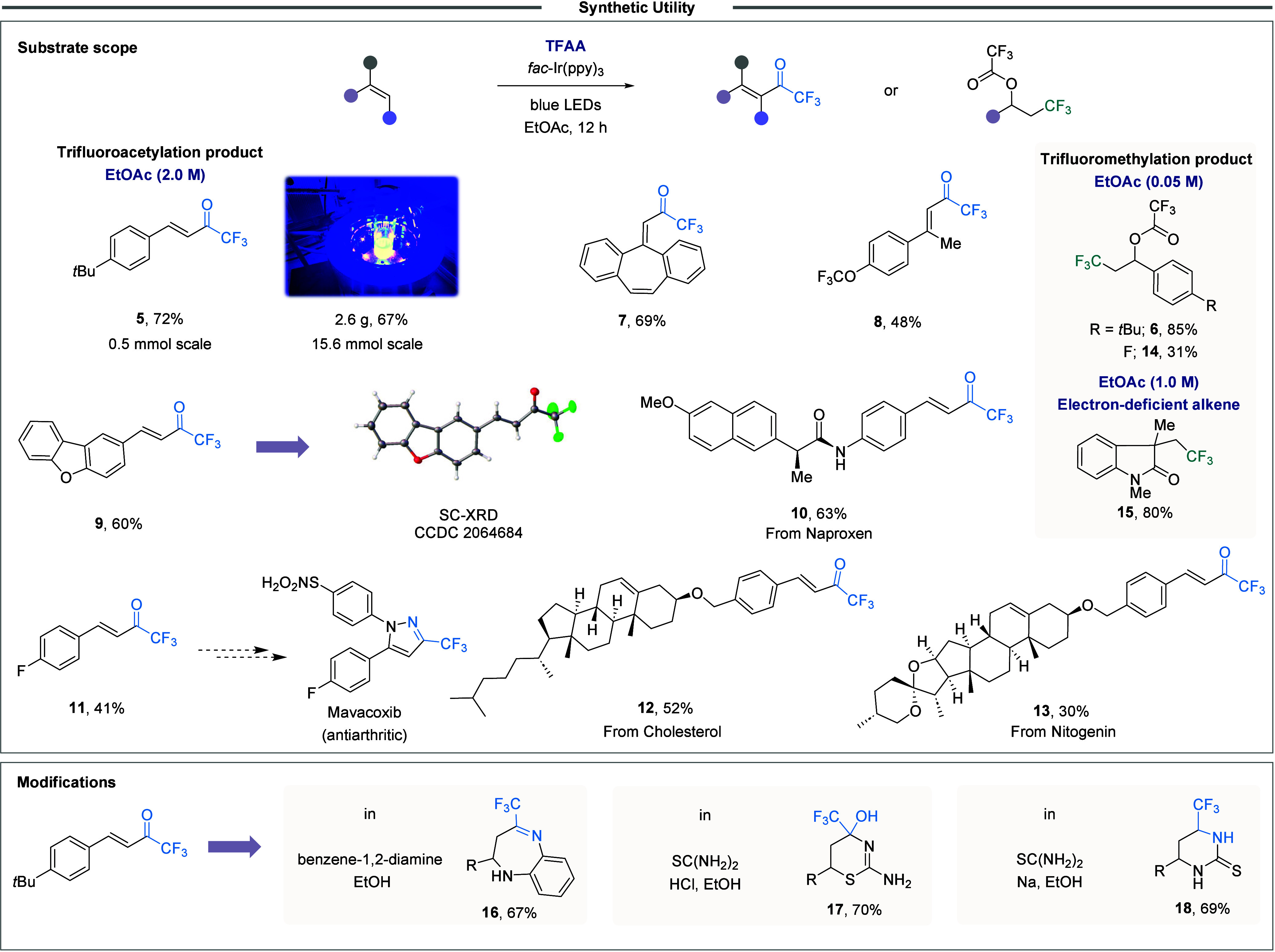
Synthetic applications of the protocol.

The mechanistic hypothesis (Figure 4A) was then investigated, the irreversible reduction of TFAA at – 1.2 V (vs SCE) confirmed by cyclic voltammetry analysis, Stern–Volmer studies revealed efficient quenching of ^3^[Ir­(ppy)_3_] triplet state by TFAA (k = 2 × 10^9^ mol^–1^ s^–1^), but not by styrene (Figure 4B). This excluded energy or electron transfer to the alkene and indicated TFAA activation. Irradiation of Ir­(ppy)_3_ in the presence of TFAA produced a broad absorption (500–700 nm) and emission (470–520 nm), consistent with Ir^IV^ formation, corroborated by spectroelectrochemical data (Figure 4B). Cyclic voltammetry analysis under irradiation showed depletion of the Ir^III^/Ir^IV^ feature, with a new oxidation peak at 0.6 V vs Fc^+^/Fc (0.98 V vs SCE),^55^ indicating irreversible catalyst oxidation. Adding 4-*tert*-butylstyrene preserved the Ir^III^/Ir^IV^ couple, suggesting regeneration of the Ir^III^ photocatalyst through oxidation of a radical intermediate.

**Figure 4:**
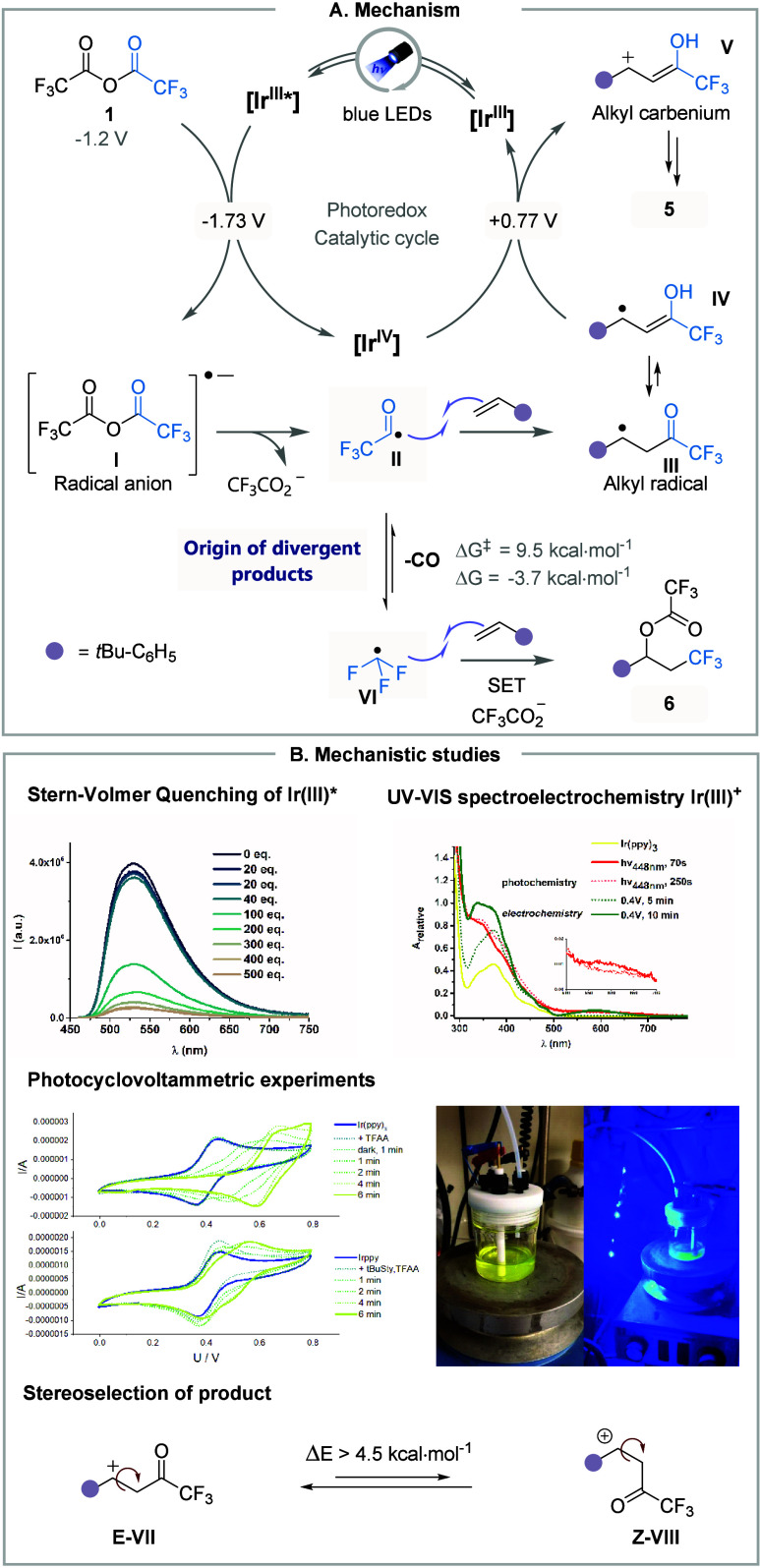
Mechanism and related studies.

The fleeting nature of the ^•^COCF_3_ radical (**II**), prone to decarbonylation to form ^•^CF_3_ (**VI**) radical and CO, was further validated by DFT calculation. These revealed a Gibbs free-energy barrier of *ΔG* ^*‡*^ = 9.5 kcal·mol^–1^ (*t*_1/2_ ≈ 0.98 μs, based on Eyring’s theory) and a reaction Gibbs free-energy of *ΔG* = −3.7 kcal·mol^–1^, consistent with a facile and reversible process. While the reduction potential of the benzylic radical **III** formed after ^•^COCF_3_ radical addition was calculated to be *E*_ox,calc_ = 0.76 V vs SCE, suggesting an essentially isoergonic back electron transfer (BET) to Ir^IV^ (*E*_1/2_ = 0.77 V vs SCE), the enol tautomerism (**IV**) which was confirmed through a deuterium labeling experiment, lowered this value to *E*_calc_ = 0.43–0.47 V vs SCE, rendering the exergonic process (*ΔG* = – 7.8 kcal·mol^–1^). Subsequent oxidation of intermediate **III**, followed by deprotonation of the enol carbocation **V**, resulted in the selective formation of (*E*)-alkene **5**. To better understand the stereoselectivity of this event, the carbenium ion conformation was studied by DFT. Calculations revealed that the minimum energy conformation of this species is indeed that of the (*E*)-conformer, with an electronic energy about 4.5 kcal·mol^–1^ lower than that of the (*Z*)-conformer (**VIII** to **VII**). Additionally, the control experiment supported that photoisomerization occurs during the reaction since subjecting (*Z*)-enone to blue light irradiation yielded a 2:1 (*E*/*Z*)-mixture, suggesting thermodynamic control. The CF_3_-product **6**, on the other hand, is formed through the addition of **VI** onto the olefin, followed by a sequence of SET and nucleophilic addition.

## 3 Activation of Chlorodifluoroacetic Anhydride

In our pursuit to investigate the chemical space of fluorinated anhydrides, with the concept of divergent synthesis at the core of our strategy, we decided to explore chlorodifluoroacetic anhydride (CDFAA) as a compelling bifunctional reagent. This compound is commonly employed as a protecting group and an electrophilic acyl donor for *O*-, *N*-, and *S*-centers (Figure 5A). The Stephenson^56^ and Sodeoka^57^ groups have demonstrated that the difluoromethylene functionality (−CF_2_-) can be effectively introduced into olefins and heteroarenes through the indirect activation of CDFAA. This strategy utilizes oxidants such as pyridinium *N*-oxide and perfluorodiacyl peroxides for efficient radical chlorodifluoromethylation.

**Figure 5:**
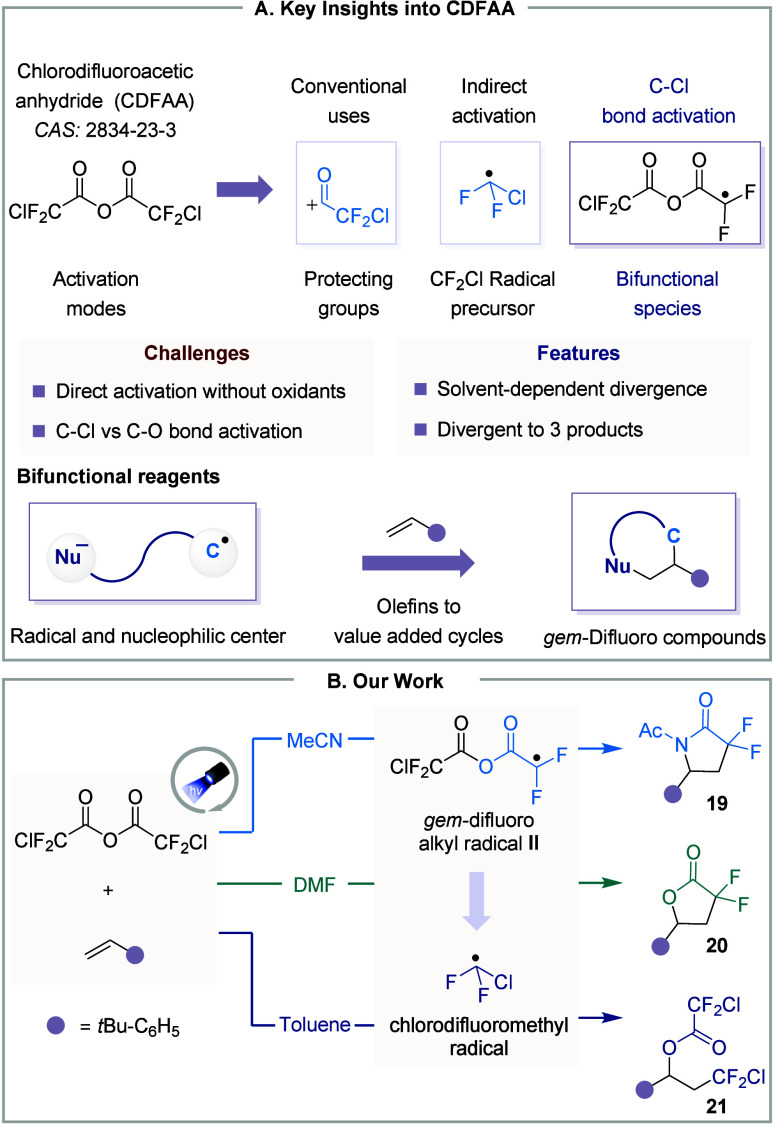
CDFAA in divergent synthesis. **A.** Key features. **B.** Approach to divergent synthesis.

These findings inspired us to explore the possibility of direct activation of chlorodifluoroacetic anhydride (CDFAA) without relying on oxidants. Nonetheless, we quickly recognized the inherent challenges, as SET reduction could lead to either an acyl radical (^•^COCF_2_Cl) via C–O cleavage or a *gem*-difluoroalkyl radical **II** via C–Cl cleavage. The weak C–O bond poses a risk of decarbonylation to ^•^CF_2_Cl. However, DFT calculations showed C–Cl cleavage is highly exergonic and irreversible, releasing the *gem*-difluoro radical and chloride ion in both solvent models (MeCN and DMF). Consistent with this, no acyl product was observed experimentally.

Notably, alkyl radical **II** has two reactive centers: a radical site and a nucleophilic oxygen atom. With this in mind, we envisioned leveraging the dual reactivity of this bifunctional synthon and hypothesized that in the presence of olefins, it could enable a new atom- and step-efficient synthetic route, leading to the formation of cyclic *gem*-difluoro compounds. After carefully designing and optimizing the reaction conditions, we screened key parameters such as photocatalyst, concentration, solvents, and other factors. This led to the establishment of conditions enabling the divergent synthesis of three different products. In MeCN, a three-component Ritter-type addition selectively formed lactam (**19**). In DMF, lactone formation (**20**) was exclusively occurred. Meanwhile, toluene was found to promote the oxy-perfluoroalkylation reaction (**21**). This demonstrates that tuning reaction conditions offers control over the selectivity of the product outcomes. The reaction demonstrated broad versatility, which highlights the applicability of the protocol (Figure 6). The reaction with α,α-disubstituted olefins yielded products **22** and **23**. Using CD_3_CN or a deuterated olefin gave products **24** and **25**. The method proved versatile, producing lactones from disubstituted styrenes (**26**, **28**, **29**) while alkynes remained unreacted (**27**). In toluene, product **30** was exclusively obtained in the reaction with 4-fluorostyrene. The protocol was equally successful for late-stage functionalization, as shown by L-camphanic acids yielding products **31**–**33** in different solvents. The D-fructopyranose derivative gave **34** and **35** in MeCN and DMF, while non-fluorinated anhydrides afforded **36** and **37**. Lactam **19** and lactone **20** were also transformed into valuable compounds **38**–**41** in a single step, which are otherwise difficult to access.

**Figure 6:**
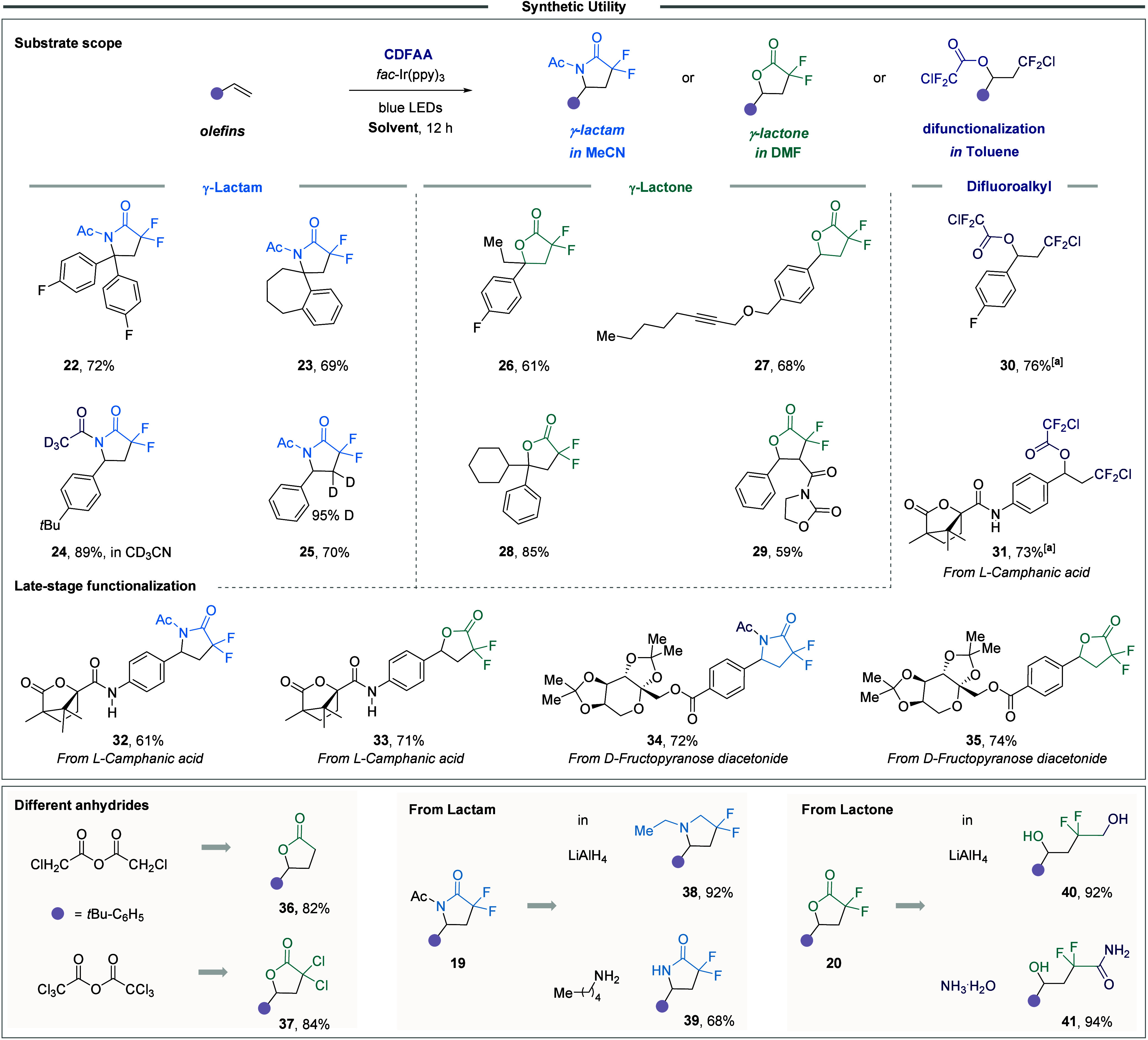
Synthetic applications of the protocol. [a] The corresponding lactone compound was formed in approximately 5% yield.

The strategic reaction mechanism is illustrated in Figure 7A. At the beginning of the photoredox catalytic cycle, Ir­(III) is the only species capable of absorbing the 440 nm blue LED light, as shown by the UV–vis spectrum (Figure 7B). Upon excitation, Ir­(III)* undergoes a SET event to form the radical anion intermediate **I**. This species then undergoes mesolytic cleavage, yielding the *gem*-difluoro carboxyalkyl radical **II** and a chloride ion. Computational data indicate this cleavage is highly exergonic in all solvent environments (*ΔG*_DMF_ = – 71.5, *ΔG*_MeCN_ = – 71.6, and *ΔG*_Toluene_ = – 42.3 in kcal·mol^–1^). The generated radical **II** adds to the olefin, yielding the stabilized alkyl radical intermediate **III**. The SET oxidation of this species by Ir­(IV) generates the corresponding carbocation **IV**. The slightly negative Hammett constants of – 0.52 for the lactam and – 0.42 for the lactone indicate that substituent effects exert only a modest influence on the overall reaction rate, making it unlikely that the SET oxidation—leading to a carbocationic intermediate—is rate-determining. Instead, the modest correlation suggests that the radical addition step is rate-determining. This interpretation is further supported by the high electrophilicity of the radical species involved (ω = 2.38 eV), which induces significant charge polarization in the transition state. At this critical point, the intermediate **IV** is intercepted by the solvent, influencing the selective formation of different products. In the case of DMF, coordination of the solvent results in intermediate **V**, while in MeCN, the selective Ritter-type addition to the carbocation **IV** takes place. The polar coordinating solvent DMF was found to be more favorable than the addition of MeCN by nearly 10 kcal·mol^–1^. To further validate this exergonic step, we conducted a competition experiment using a solvent mixture of DMF and MeCN, where even with just 10% DMF in MeCN, the lactone product predominated. The intramolecular cyclization of intermediates **V** and **VI** leads to the formation of products **20** and **19**, respectively, through subsequent reaction steps.

**Figure 7:**
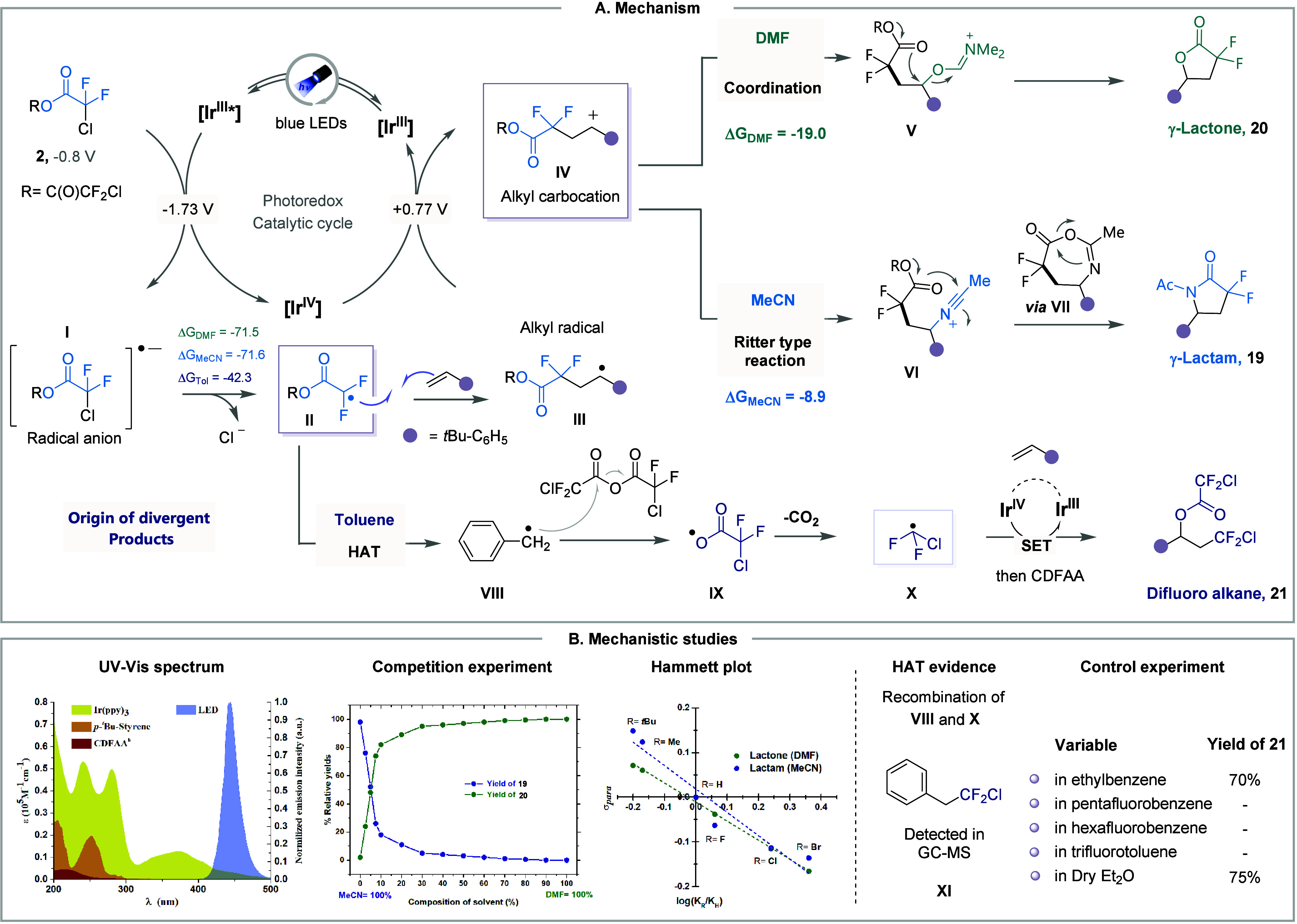
**A.** Underlying reaction pathways and origin of the divergent synthesis. **B.** Key mechanistic experiments.

A final series of experiments was conducted to elucidate the mechanism leading to the formation of product **21**. Experimental evidence suggests the process likely involves a HAT from intermediate **II** to generate a tolyl radical (**VIII**). This radical predominantly reacts with the anhydride to form a carboxyl radical **IX**, which then undergoes decarboxylation to generate the ^•^CF_2_Cl radical (**X**). The recombination of **VIII** and **X** was observed by GC-MS, and this process occurs only in solvents capable of undergoing HAT, supporting the proposed pathway. Furthermore, radical **X** adds to the olefin, followed by an RPC process with Ir­(IV), ultimately leading to the formation of the difunctionalized product **21**. Although spontaneous C–O bond fragmentation of **I** was not observed during our DFT studies, it was computed to be more exergonic than the C–Cl bond cleavage in toluene. Therefore, the straightforward formation of radical **X** through C–O mesolytic cleavage cannot be excluded.

## 4 Activation of Chlorodifluoroacetic Acid

As seen so far, anhydrides have proven to be excellent precursors for the synthesis of *gem*-difluoro cycles, making them valuable reagents in this transformation. However, their synthesis originates from corresponding acids or acyl chlorides, reducing efficiency in terms of atom- and step economy. Furthermore, anhydrides are moisture sensitive, which can lead to synthetic complications. To overcome this limitation, we employed α-halodifluoroacetic acids, particularly CDFA, as a cost-effective, widely available redox-active reagent for *gem*-difluoro synthons. We hypothesized that upon activation of the C–Cl bond, it could generate a *gem*-difluoro carboxy alkyl radical (Figure 8A), which could also serve as a bifunctional species in the presence of an olefin molecule. Additionally, it also holds potential for delivering divergent products upon solvent and additive switch (Figure 8B).

**Figure 8:**
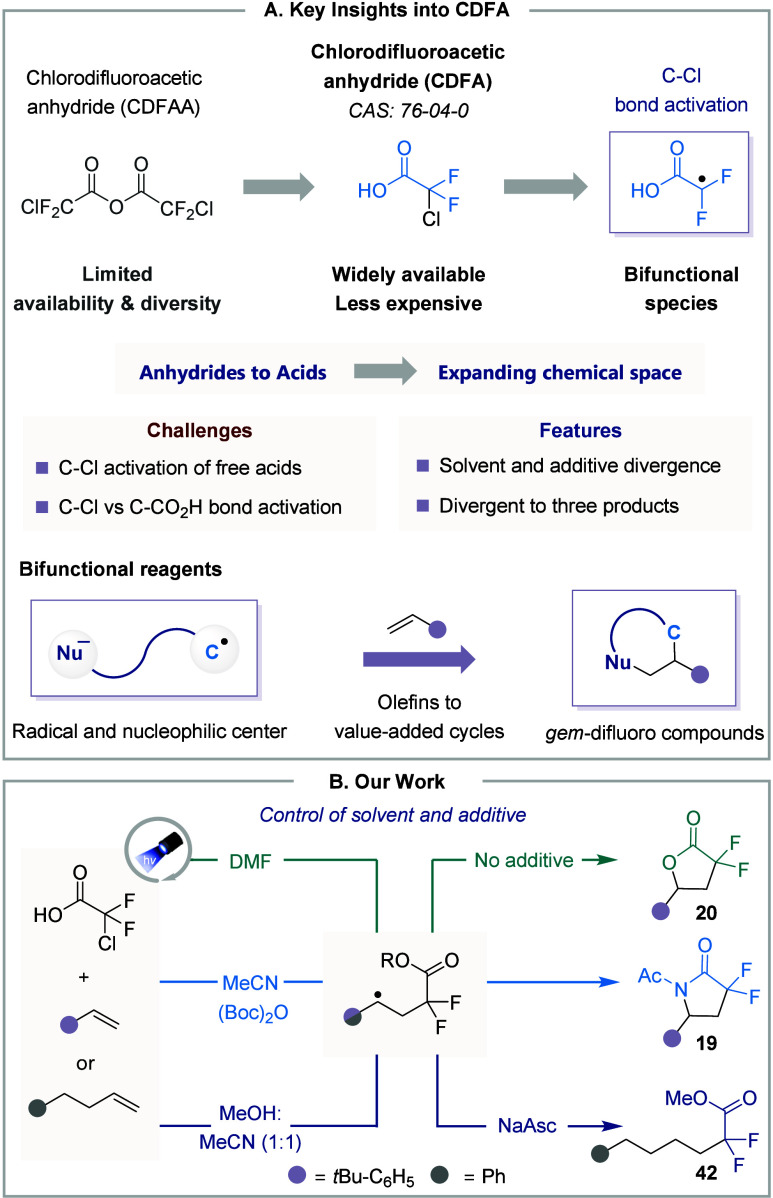
CDFA as redox active reagent. **A.** Key features. **B.** Approach to divergent synthesis.

After extensive optimization, we identified a straightforward condition for synthesizing lactone **20**. However, lactam formation proved challenging, as the reaction predominantly yielded lactone adduct. We attributed this to the preferential intramolecular cyclization of the acid’s oxygen rather than MeCN addition to the carbocation. To suppress the acid’s nucleophilicity, we introduced Boc-anhydride to form a mixed anhydride *in situ*, which successfully facilitated lactam formation **19**. Encouraged by this result, we aimed to gain further control over the alkyl radical intermediate and found that sodium ascorbate (NaAsc) effectively serves as a HAT reagent, while MeOH engages in esterification of the acid group, thereby facilitating the formation of linear difluoro esters in a single step (**42**). After optimizing the formation of γ-lactones, γ-lactams, and difluoromethyl esters, we applied the method to a variety of alkenes. The representative examples are summarized in Figure 9. Lactone formation proved to be highly productive. For instance, styrene with benzylic chlorine yielded **43**, while indene afforded product **44**. For compound **45**, the reaction occurred exclusively at the exocyclic olefin motif and was confirmed by X-ray. Although lactam synthesis was less efficient, we successfully obtained the products from indene and estrone derivatives, **46** and **47**, respectively.

**Figure 9:**
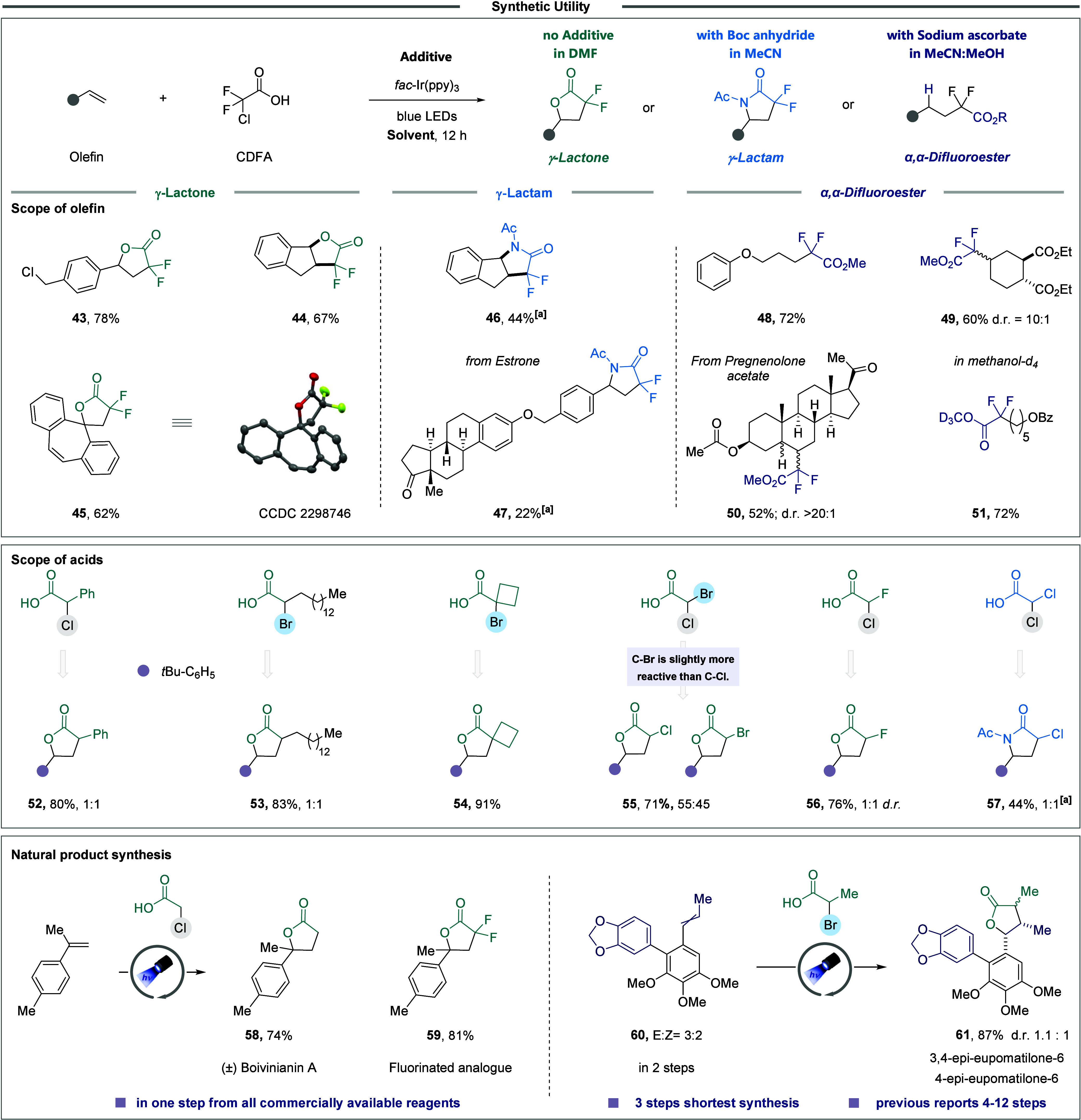
Synthetic applications of the protocol. [a] The corresponding lactone compound was formed in approximately 10% yield.

One of the primary challenges in our previous studies involving anhydrides and acids under Ir-photocatalyzed conditions was the lack of reactivity with nonstyrenyl olefins. It is generally encountered in the literature that this photocatalyst is unable to oxidize alkyl radicals,^58–60^ despite the oxidation potential of secondary alkyl radicals being only slightly higher than benzylic radicals (*E*_1/2_ (iPr^•^) = 0.47 V > *E*_1/2_ (PhCHMe^•^) = 0.37 V, vs SCE),^61^ a value that remains lower than that for the reduction of Ir^IV^(ppy)_3_ (*E*_1/2_ [Ir^IV^/Ir^III^] = 0.77 V vs SCE). One can assume that the absence of an aromatic group in alkyl derivatives disfavors the formation of the complex necessary for the SET event to occur between Ir^IV^(ppy)_3_ and the alkyl radical, therefore inhibiting the RPC pathway.^62^ To overcome this challenge, we relied on an alternative approach consisting in closing the Ir^IV^/Ir^III^ catalytic cycle with an external reductant. After a thorough analysis of literature reports, we found that sodium ascorbate (NaAsc) could not only reduce the photocatalyst but also serve as a HAT reagent, playing a dual role in the reaction.^63,64^ Building on these results, we broadened the scope of α,α-difluoroester synthesis beyond styrenes, successfully reacting a variety of unactivated olefins and achieving good yields. Benzyl ethers, cyclic olefins, and pregnenolone acetate all participated in the reaction, yielding products **48**–**50** in good yields. Notably, when *d*_3_-MeOH was employed, the reaction resulted in the formation of the CD_3_-incorporated product (**51**). Our methodology thus provides a straightforward platform for accessing various *gem*-difluoro esters from a single acid precursor by simply varying the alcohol additive, unlike traditional methods that rely on preformed esters.^65^ We also examined different halogen substitutions at the α-position of the acid group. Our findings revealed that fluorine remained untouched under the reaction conditions, while chlorine exhibited slightly less reactivity than bromine (**55**–**57**), with the reactivity order being (F ≪ Cl < Br).

This developed protocol was also applied to the concise synthesis of bioactive compounds. We efficiently synthesized (±)-Boivinianin A (**58**) and its fluorinated analogue (**59**) in a single step from all commercially available starting materials. Additionally, we achieved the most step-efficient synthesis reported for a mixture of (±)-3,4-epi- and 4-*epi*-eupomatilone-6 (**61**). The mechanism for the formation of lactam **19** and lactone **20** begins with an irreversible SET of *gem*-difluoroacetic acids (**3**), followed by the subsequent reaction with olefins, achieving divergent synthesis in a similar manner in MeCN and DMF, as described in Figure 7A.

Moreover, a mechanism for the synthesis of the hydrofunctionalized product **42** in MeOH:MeCN via a NaAsc-mediated HAT mechanism is outlined in Figure 10, highlighting the key steps in the reaction pathway. The process begins with the conversion of CDFA to its ester under acidic conditions, leading to the formation of *gem*-chlorodifluoroester **I** at room temperature. Upon photoexcitation, the Ir photocatalyst facilitates the SET reduction of **I**, generating radical anion **II**. Subsequent mesolytic cleavage of **II** furnishes radical **III**, which undergoes radical addition to the unactivated olefin, forming the alkyl radical **IV**. This intermediate then participates in a HAT with NaAsc, ultimately yielding product **42** and ascorbate radical anion. NaAsc plays a crucial additional role in this reaction, as it is also required to reduce Ir­(IV) back to Ir­(III), thereby closing the catalytic cycle, while simultaneously producing radical **VI**. In line with our proposed mechanism, the facile oxidation of benzylic radicals produced from styrene substrates predominantly leads to lactone as the major product through an RPC mechanism. In contrast, the challenging oxidation of nonstabilized radicals generated from unactivated olefins favors the HAT pathway. This observation clearly indicates that the SET process is significantly more efficient and faster than HAT under these conditions.

**Figure 10:**
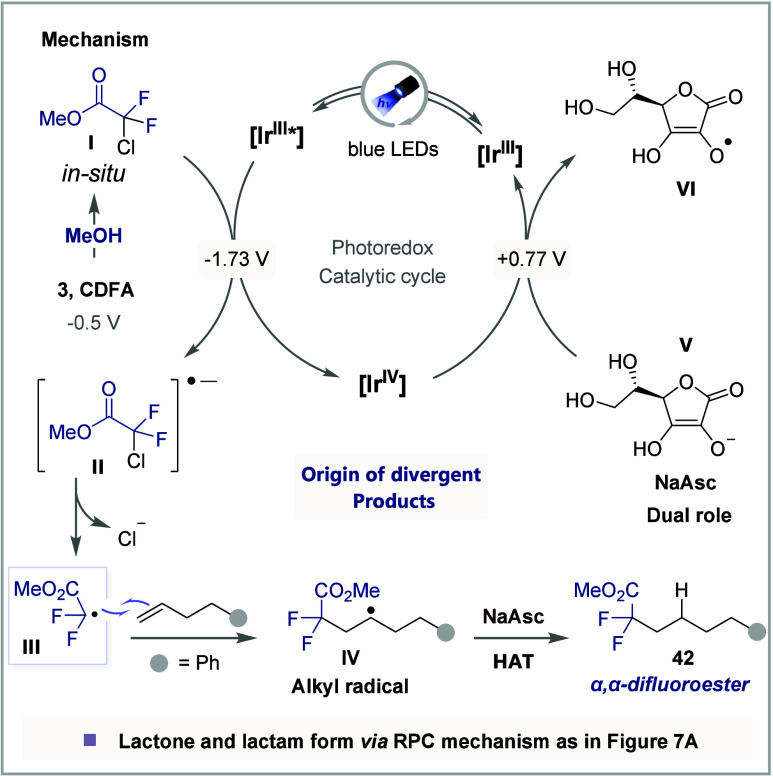
NaAsc-mediated HAT mechanism.

## 5 Activation of α-Halotrifluoromethyl Ketones

During our investigation on the activation of TFAA for the generation of trifluoroacetyl radical, we encountered a significant challenge related to the limited lifetime of ^•^C­(O)­CF_3_ radical due to its dissociation into CO and ^•^CF_3_ radical. To address this challenge, we envisioned that direct activation of chlorotrifluoroacetone could enable the synthesis of the homologated trifluoromethylketones through the generation of a stable ^•^CH_2_COCF_3_ moiety (Figure 11A).

**Figure 11:**
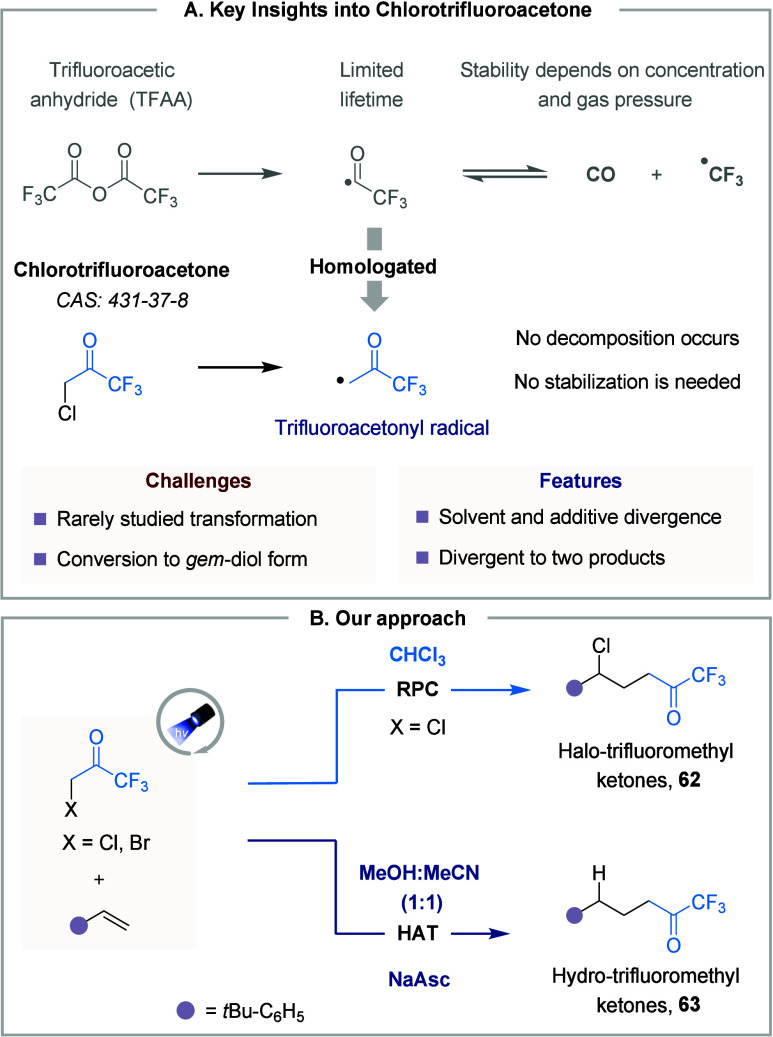
Trifluoromethyl ketones in divergent synthesis. **A.** Key features. **B.** Approach to divergent synthesis.

By employing an Ir-catalyzed photoredox strategy, we successfully developed a protocol enabling selective and divergent access to hydro- and halo-trifluoromethyl ketones through precise control of reaction intermediates. We revealed that in chloroform, the reaction follows an RPC mechanism, yielding halo-trifluoromethylated products. Alongside, switching the solvent system to MeOH: MeCN and employing sodium ascorbate (NaAsc) as the HAT reagent allowed for the selective formation of hydro-trifluoromethyl ketones (Figure 11B). After establishing the protocols, we first explored the application of chloro-trifluoromethyl ketones and found that this process is only limited to styrenes (**62**, **64**).

The reaction proceeds via a similar SET reduction of reagent **4**, generating the trifluoroacetonyl radical, which adds to the olefin to form alkyl radical intermediate **I** (Figure 12B). The presence of species **I** was confirmed using TEMPO as a radical trap (Figure 12B). Following a subsequent SET event through RPC mechanism, the ion pair collapse between carbenium ion **II** and the chloride anion yields the halo-trifluoromethyl ketone product **62** (*ΔG* = −60.0 kcal·mol^–1^). The RPC mechanistic pathway is followed when the reaction is carried out with a Cl-based reagent, whereas the XAT mechanism is favored when a Br-based reagent is used. Therefore, the reactivity can be extended beyond styrenes to include less activated unsaturated hydrocarbons (**65**, **66**). This difference is likely attributed to a radical chain process via a halogen atom transfer (XAT) mechanism, where the alkyl radical abstracts a bromine atom from reagent **4**.^66^

**Figure 12:**
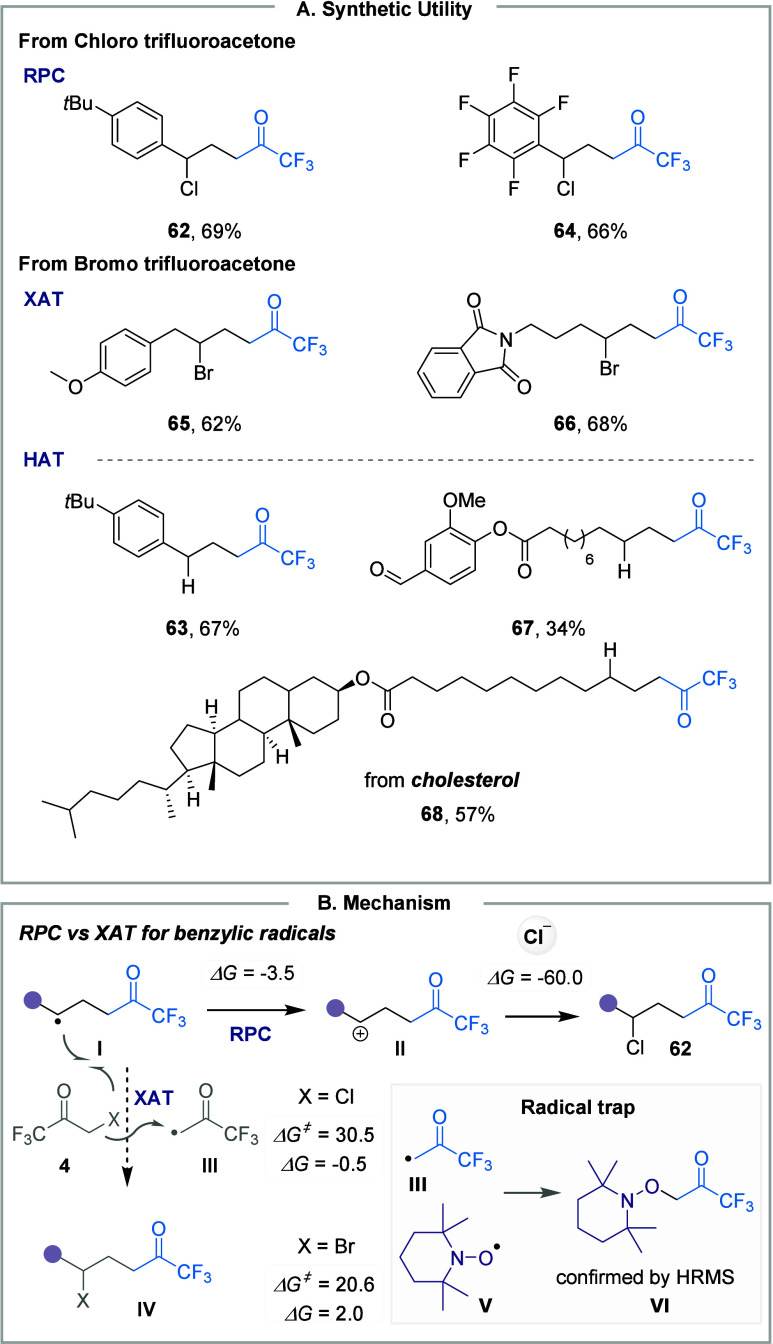
**A.** Synthetic applications of the protocol. **B.** Mechanism involving benzylic radicals.

In the case of styrene substrate, DFT calculations revealed that the radical chain pathway involving a benzylic radical was unfavorable (*ΔG* ^*‡*^ = 20.6, *ΔG* = +2.0 in kcal·mol^–1^) compared to the exergonic RPC process (*ΔG* = −3.5 kcal·mol^–1^) (Figure 12B). We demonstrated again the dual role of NaAsc in this study, enabling the divergent synthesis of products **63, 67** and **68**. In the presence of NaAsc, exclusive formation of the hydro-functionalization product was observed following a HAT event (*ΔG* ^*‡*^ = 17.2, *ΔG* = −19.7 in kcal·mol^–1^).

## 6 Conclusions, Outlook, and Challenges

This account highlights our journey in advancing organofluorine and radical chemistry, demonstrating how photoredox catalysis serves as a powerful tool for the direct activation of bulk prepared chemicals. The unique reactivity of fluorinated radicals has enabled highly selective bond formations under mild conditions, unlocking new pathways for constructing divergent products from common reactive intermediates. Our development of switchable divergent synthesis, in which reaction conditions dictate distinct product outcomes from a common radical intermediate, represents a significant achievement. By harnessing electronic effects and tunable reaction environments, we have demonstrated that fluorinated radicals can be selectively directed toward different functionalization pathways with high selectivity. By capitalizing on the power of fluorinated radicals and the strategic implementation of switchable reaction manifolds, we anticipate continued innovation at the interface of radical chemistry and modern organic synthesis, shaping the next generation of functional molecules.

Moving forward, the development of novel redox-active reagents capable of generating elusive radicals for the selective functionalization of hydrocarbons within the framework of switchable divergent synthesis represents an exciting research direction for advancing synthetic methodology and expanding the scope of radical chemistry. Several promising routes for further exploration include developing asymmetric strategies for constructing cyclic structures such as lactams and lactones, as well as activating widely available feedstock materials, particularly those without carbonyl functional groups, such as alkyl chlorides, to enable olefin functionalization and broaden the chemical space of organofluorine compounds. Additionally, we foresee that insights from computational studies will further enhance our understanding of applied theoretical chemistry, enabling more precise reaction design and a deeper comprehension of underlying mechanisms involving fluorinated species.

Although concerns over fluorinated compounds, known as ″forever chemicals,″ have led to legal actions, production discontinuation, and calls for stricter regulations, these issues are crucial and deserve focused attention.^67^ Importantly, recent breakthroughs in the degradation of PFAS instil a lot of promises and inspiring opportunities for the field.^68–72^ Nevertheless, the smaller organofluorine compounds discussed in this account are less toxic than PFAS and therefore lie outside the scope of the present discussion. Here, we focus on the beneficial role of fluorine in synthetic organic chemistry, where fluorinated motifs continue to drive innovation in medicinal chemistry, materials science, and other fields, leveraging fluorine’s unique electronic and steric properties to enhance molecular performance.

## Supplementary Material



## Notes

Additional computational details and methods: philicity of radicals and other parameters (Table S1); computed energies for the reactivity scale (Table S2); linear regressions (Figure S1); computed energies for the decarbonylation versus radical addition (Tables S3–4); computed geometries (xyz coordinates) (PDF)
