# Supplementary material for: Fluorinated Radicals in Divergent Synthesis via Photoredox Catalysis
Source: Acc Chem Res. 2025 Jun 11;58(13):2046–60. doi: 10.1021/acs.accounts.5c00239 (PMC12224337; doi:10.1021/acs.accounts.5c00239)
Supplement: Supplementary file 1 [file ar5c00239_si_001.pdf]

Fluorinated Radicals in Divergent Synthesis *via* Photoredox Catalysis

Rahul Giri,†[a] Anthony J. Fernandes,†[a] Dmitry Katayev*[a]

[a] Department für Chemie und Biochemie, Universität Bern, Freiestrasse 3, 3012 Bern (Switzerland)

† These authors contributed equally to this work

* Corresponding author

Email: dmitry.katayev@unibe.ch

1. COMPUTATIONAL DETAILS 2
1.1. COMPUTATIONAL METHOD 2
1.2. COMPUTED PARAMETERS 3
1.3. REACTIVITY SCALE: RADICAL ADDITION TO STYRENE 5
1.4. LINEAR REGRESSIONS 11
1.5. DECARBONYLATION *VS* RADICAL ADDITION 12
1.6. COORDINATES (XYZ) 13
1.6.1. Philicity parameters 13
1.6.2. Reactivity scale 15
1.6.3. Decarbonylation vs radical addition 30
2. REFERENCES 31

1. Computational details

1.1. Computational method

The DFT calculations have been performed with the Gaussian 9 program package.[1]

The conformational space of all molecules has been initially searched using meta-dynamics simulations based on tight-binding quantum chemical calculations as implemented in the software package Conformer-Rotamer Ensemble Sampling Tool CREST.[2]

Global electrophilicity indices were calculated as reported by De Proft *et al.* in gas phase at the (U)B3LYP-D3(BJ)/6-311+G(d,p) level of theory.[3]

The reactivity scale was established as follows:

The structures located with CREST have been subjected to (U)M062X-D3/def2-TZVP,SMD(MeCN) geometry optimization.[4] The nature of all stationary points (minima and transition states) was verified through the computation of the vibrational frequencies. Single point energies from these geometries were calculated at the DLPNO-(U)CCSD(T)/cc-pVTZ,SMD(MeCN) level of theory using ORCA version 5.0.3.[5] Quasiharmonic corrections to enthalpy (Head-Gordon) and entropy (Grimme) were made using Paton's GoodVibes software[6] (ver. 3.2) and incorporated single-point corrections to energy. Temperature corrections were applied to 298.15 K (25 °C) and pressure to 1 atm unless otherwise specified. All energies are reported in kcal mol^{-1} unless otherwise stated.

The polarizable continuum model (PCM) with SMD parameters[7] was applied to consider solvent effects for both geometries and energies. SMD parameters of acetonitrile and ethyl acetate are available in the software package used.

1.2. Computed parameters

Table S1. Radical parameters computed by DFT at the (U)B3LYP-D3(BJ)/6-311+G(d,p) level of theory. *IP*: Ionization Potential, *EA*: Electron Affinity, η: Chemical hardness, μ: Chemical potential (-Electronegativity), ω: Global Electrophilicity index, ω-: Nucleophilicity index. (For some radicals discussed in this manuscript, global electrophilicity indices and other parameters were previously computed in the original studies).[8]

	**Parameters in gas phase (U)B3LYP-D3(BJ)/6-311+G(d,p)**					
**Radical**	***IP* (eV)**	***EA* (eV)**	**η (eV)**	**μ (eV)**	**ω (eV)**	**ω- (eV)**
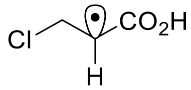	9.70	2.09	7.61	-5.90	2.284	0.178
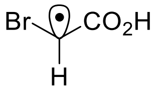	9.35	1.76	7.59	-5.55	2.034	0.204
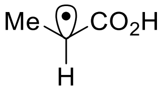	9.25	1.17	8.08	-5.21	1.677	0.237
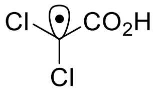	9.07	1.80	7.27	-5.44	2.033	0.211
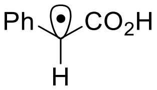	7.82	1.86	5.96	-4.84	1.966	0.244
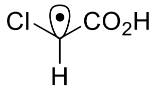	9.54	1.62	7.92	-5.58	1.966	0.205
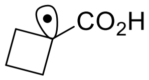	8.49	1.12	7.37	-4.81	1.566	0.266
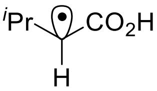	8.92	1.32	7.60	-5.12	1.727	0.240
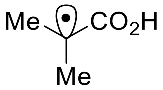	8.51	1.01	7.50	-4.76	1.509	0.272
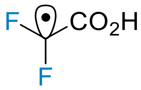	9.75	1.12	8.63	-5.43	1.709	0.222
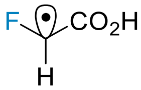	10.00	1.25	8.75	-5.62	1.807	0.206
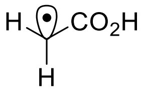	10.40	1.53	8.87	-5.96	2.006	0.180
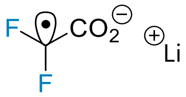	8.88	0.55	8.32	-4.71	1.335	0.285
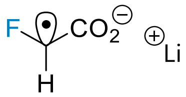	9.00	0.54	8.46	-4.77	1.343	0.281
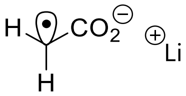	9.37	0.78	8.59	-5.07	1.499	0.253
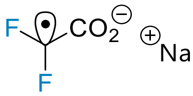	8.41	0.39	8.02	-4.40	1.207	0.313

	Parameters in gas phase (U)B3LYP-D3(BJ)/6-311+G(d,p)					
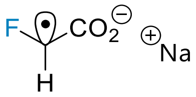	8.41	0.35	8.06	-4.38	1.189	0.315
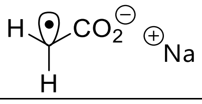	8.76	0.48	8.28	-4.62	1.288	0.294
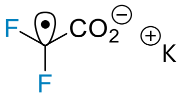	8.09	0.20	7.89	-4.15	1.089	0.337
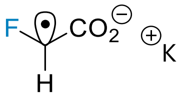	8.07	0.17	7.91	-4.12	1.073	0.340
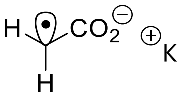	8.42	0.29	8.12	-4.35	1.167	0.318

1.3. Reactivity scale: Radical addition to styrene

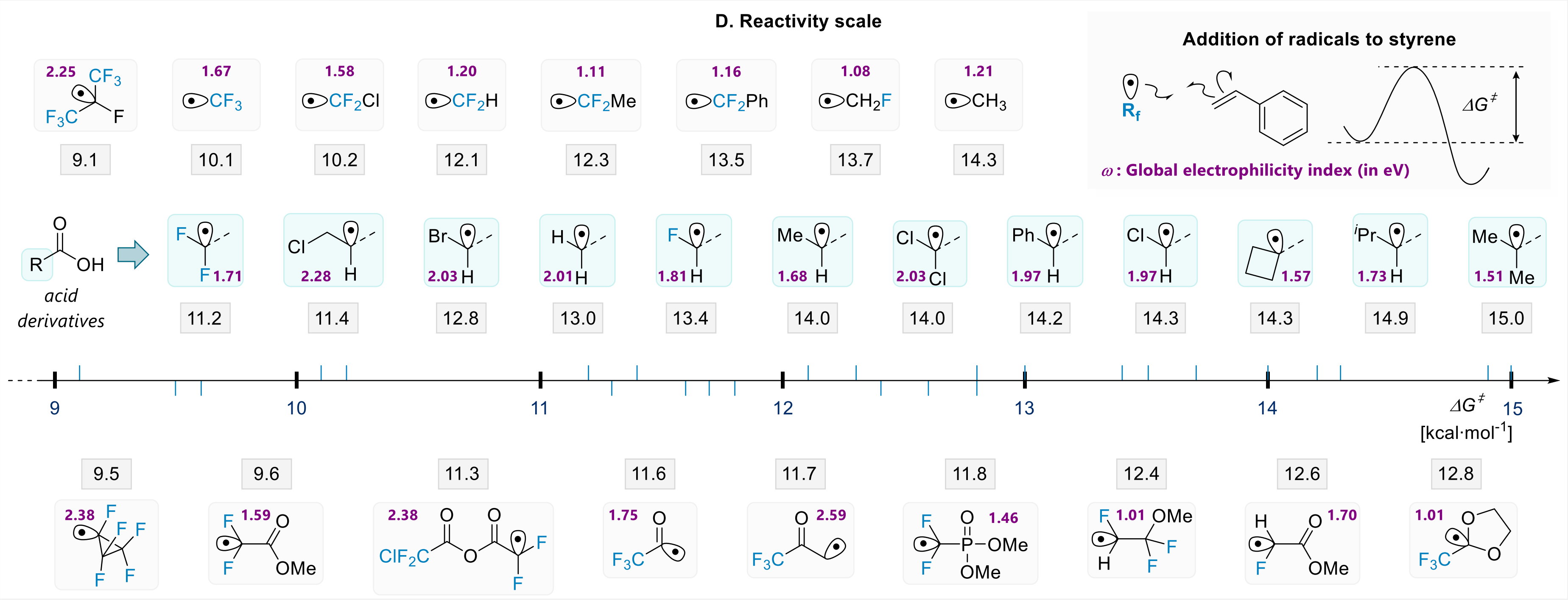

Table S2. Computed energies at the DLPNO-(U)CCSD(T)/cc-pVTZ,SMD(MeCN)//(U)M062X-D3/Def2TZVP,SMD(MeCN) level of theory.

		DLPNO-(U)CCSD(T)/cc-pVTZ,SMD(MeCN)//(U)M062X-D3/Def2TZVP,SMD(MeCN)								
		In Hartree								In kcal/mol
**Species**	**Im. Freq.**	***E_SPC***	***E***	***ZPE***	***H_SPC***	***T.S***	***T.qh-S***	***G(T)_SPC***	***qh-G(T)_SPC***	***ΔG***
**Styrene**		-309.084269	-309.628023	0.130267	-308.946252	0.038799	0.038503	-308.985051	-308.984755	
$\cdot CF_3$		-337.194877	-337.597186	0.011913	-337.178568	0.031088	0.031089	-337.209655	-337.209656	0
**TS**	-285.37	-646.282972	-647.229387	0.142703	-646.128045	0.052903	0.050289	-646.180948	-646.178335	**10.1**
**P**		-646.360002	-647.303128	0.145049	-646.203397	0.050116	0.048533	-646.253514	-646.25193	-36.1

		DLPNO-(U)CCSD(T)/cc-pVTZ,SMD(MeCN)//(U)M062X-D3/Def2TZVP,SMD(MeCN)								
		In Hartree								In kcal/mol
**Species**	**Im. Freq.**	***E_SPC***	***E***	***ZPE***	***H_SPC***	***T.S***	***T.qh-S***	***G(T)_SPC***	***qh-G(T)_SPC***	***ΔG***
$^{\bullet}CF_2Cl$		-697.185425	-697.938517	0.010121	-697.170574	0.032505	0.032507	-697.203079	-697.203082	0
**TS**	-284.89	-1006.273825	-1007.570521	0.140983	-1006.120251	0.054148	0.051387	-1006.174399	-1006.171639	**10.2**
**P**		-1006.343387	-1007.636263	0.143749	-1006.187759	0.05124	0.049303	-1006.238999	-1006.237061	-30.9
$^{\bullet}CF_2H$		-238.047244	-238.33568	0.018769	-238.024413	0.029085	0.029085	-238.053498	-238.053499	0
**TS**	-418.94	-547.131521	-547.964306	0.1497	-546.970219	0.051067	0.048732	-547.021285	-547.018951	**12.1**
**P**		-547.203067	-548.032954	0.153944	-547.038345	0.047959	0.046518	-547.086304	-547.084863	-29.2
$^{\bullet}CF_2Me$		-277.304077	-277.658389	0.045926	-277.252845	0.032668	0.032672	-277.285513	-277.285517	0
**TS**	-353.61	-586.38862	-587.286765	0.176413	-586.199168	0.053721	0.051484	-586.252889	-586.250652	**12.3**
**P**		-586.456389	-587.351212	0.180202	-586.264036	0.050531	0.049023	-586.314568	-586.313059	-26.8
$^{\bullet}CF_2Ph$		-468.693815	-469.397531	0.09708	-468.587982	0.042683	0.042005	-468.630666	-468.629988	0
**TS**	-412.65	-777.779364	-779.026018	0.229154	-777.534151	0.063339	0.059007	-777.597489	-777.593158	**13.5**
**P**		-777.839126	-779.081601	0.2322	-777.591507	0.059452	0.056525	-777.650959	-777.648032	-20.9
$^{\bullet}CH_2F$		-138.898926	-139.073199	0.024186	-138.870757	0.026736	0.026736	-138.897492	-138.897492	0
**TS**	-468.94	-447.980626	-448.699227	0.156408	-447.813225	0.049087	0.047194	-447.862312	-447.860419	**13.7**
**P**		-448.049074	-448.76576	0.161088	-447.877795	0.045957	0.045051	-447.923752	-447.922846	-25.5
$^{\bullet}CH_3$		-39.761876	-39.82237	0.028655	-39.729138	0.02392	0.023921	-39.753058	-39.753059	0
**TS**	-511.18	-348.842853	-349.447133	0.162406	-348.670134	0.046249	0.044826	-348.716383	-348.714961	**14.3**
**P**		-348.906986	-349.511915	0.167181	-348.730167	0.043819	0.04329	-348.773986	-348.773457	-22.4
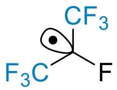		-812.261917	-813.245762	0.036221	-812.215635	0.04683	0.045384	-812.262464	-812.261019	0
**TS**	-293.75	-1121.354063	-1122.880535	0.167119	-1121.169194	0.066038	0.062032	-1121.235232	-1121.231226	**9.1**
**P**		-1121.416616	-1122.939079	0.170488	-1121.229238	0.062478	0.059614	-1121.291716	-1121.288851	-27.0
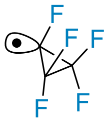		-612.666769	-613.434269	0.028685	-612.630045	0.040762	0.040601	-612.670806	-612.670646	0
**TS**	-221.65	-921.756955	-923.068171	0.159274	-921.581676	0.062389	0.058631	-921.644065	-921.640307	**9.5**

		DLPNO-(U)CCSD(T)/cc-pVTZ,SMD(MeCN)//(U)M062X-D3/Def2TZVP,SMD(MeCN)								
		In Hartree								In kcal/mol
**Species**	**Im. Freq.**	***E_SPC***	***E***	***ZPE***	***H_SPC***	***T.S***	***T.qh-S***	***G(T)_SPC***	***qh-G(T)_SPC***	***ΔG***
**P**		-921.829362	-923.135755	0.163103	-921.651137	0.058005	0.055957	-921.709142	-921.707094	-32.4
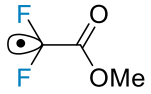		-465.605363	-466.226416	0.060607	-465.536212	0.041737	0.041383	-465.577949	-465.577595	0
**TS**	-403.52	-774.698064	-775.859633	0.192209	-774.490224	0.058994	0.056804	-774.549218	-774.547028	**9.6**
**P**		-774.758589	-775.915099	0.195252	-774.548271	0.057731	0.055516	-774.606003	-774.603788	-26.0
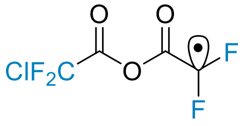		-1236.196022	-1237.670537	0.045997	-1236.137632	0.053173	0.051021	-1236.190805	-1236.188653	0
**TS**	-363.65	-1545.284921	-1547.301079	0.177119	-1545.087751	0.073394	0.067672	-1545.161145	-1545.155424	**11.3**
**P**		-1545.344212	-1547.353657	0.179464	-1545.145004	0.072676	0.066913	-1545.21768	-1545.211917	-24.2
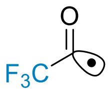		-450.369562	-450.933586	0.020774	-450.342412	0.036548	0.036277	-450.378961	-450.378689	0
**TS**	-167.00	-759.458178	-760.56829	0.152593	-759.291873	0.055578	0.05305	-759.347451	-759.344923	**11.6**
**P**		-759.513729	-760.621837	0.153989	-759.346116	0.055523	0.053096	-759.401639	-759.399212	-22.4
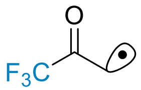		-489.626375	-490.258748	0.047735	-489.571157	0.03924	0.038661	-489.610398	-489.609819	0
**TS**	-371.62	-798.714794	-799.889889	0.17951	-798.520329	0.058362	0.055561	-798.578691	-798.57589	**11.7**
**P**		-798.758372	-799.931733	0.181471	-798.561712	0.061552	0.057416	-798.623265	-798.619128	-15.4
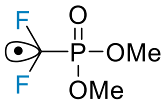		-883.529185	-884.682187	0.099487	-883.417539	0.050815	0.049493	-883.468354	-883.467032	0
**TS**	-342.85	-1192.616153	-1194.3115	0.230192	-1192.365863	0.072231	0.067121	-1192.438095	-1192.432984	**11.8**
**P**		-1192.680556	-1194.372584	0.234024	-1192.427544	0.067056	0.063905	-1192.494599	-1192.491448	-24.9
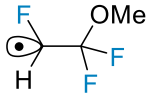		-490.812147	-491.441541	0.070102	-490.733421	0.042023	0.041251	-490.775444	-490.774672	0

		DLPNO-(U)CCSD(T)/cc-pVTZ,SMD(MeCN)//(U)M062X-D3/Def2TZVP,SMD(MeCN)								
		In Hartree								In kcal/mol
**Species**	**Im. Freq.**	***E_SPC***	***E***	***ZPE***	***H_SPC***	***T.S***	***T.qh-S***	***G(T)_SPC***	***qh-G(T)_SPC***	***ΔG***
**TS**	-437.85	-799.899058	-801.071043	0.201758	-799.681233	0.062124	0.058408	-799.743357	-799.739642	**12.4**
**P**		-799.964162	-801.134026	0.205409	-799.743394	0.058863	0.056399	-799.802257	-799.799793	-25.3
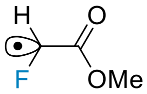		-366.470453	-366.975945	0.068192	-366.394755	0.038505	0.038393	-366.43326	-366.433148	0
**TS**	-490.79	-675.55789	-676.605221	0.200012	-675.343208	0.056238	0.054564	-675.399446	-675.397772	**12.6**
**P**		-675.610894	-676.65483	0.203241	-675.393276	0.05617	0.054086	-675.449446	-675.447362	-18.5
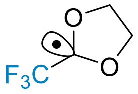		-603.994382	-604.789676	0.083855	-603.901629	0.04347	0.04247	-603.945099	-603.944099	0
**TS**	-324.06	-913.07949	-914.417348	0.214326	-912.848506	0.064318	0.060015	-912.912824	-912.908521	**12.8**
**P**		-913.134924	-914.469555	0.217468	-912.901535	0.06035	0.057619	-912.961886	-912.959155	-19.0
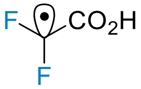		-426.379863	-426.933844	0.033139	-426.339888	0.037266	0.037213	-426.377154	-426.377102	0
**TS**	-407.07	-735.468107	-736.563575	0.164409	-735.289395	0.057646	0.054646	-735.347041	-735.344041	**11.2**
**P**		-735.530829	-736.620541	0.167192	-735.349907	0.055857	0.053088	-735.405764	-735.402995	-25.8
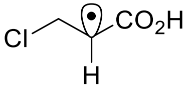		-726.499987	-727.352558	0.067251	-726.425244	0.039893	0.039091	-726.465137	-726.464335	0
**TS**	-447.65	-1035.588643	-1036.982325	0.198998	-1035.374645	0.059447	0.05623	-1035.434092	-1035.430876	**11.4**
**P**		-1035.634376	-1037.026298	0.201778	-1035.417824	0.05943	0.056144	-1035.477254	-1035.473968	-15.6
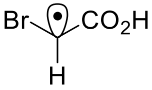		-2800.227483	-2802.033005	0.038748	-2800.182464	0.03704	0.036972	-2800.219504	-2800.219436	0
**TS**	-434.63	-3109.313778	-3111.66019	0.170311	-3109.129706	0.056688	0.054124	-3109.186393	-3109.18383	**12.8**
**P**		-3109.365431	-3111.708786	0.173671	-3109.178243	0.056167	0.053533	-3109.23441	-3109.231776	-17.3
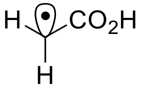		-228.109394	-228.433058	0.047203	-228.056979	0.032045	0.032048	-228.089023	-228.089027	0

		DLPNO-(U)CCSD(T)/cc-pVTZ,SMD(MeCN)//(U)M062X-D3/Def2TZVP,SMD(MeCN)								
		In Hartree								In kcal/mol
**Species**	**Im. Freq.**	***E_SPC***	***E***	***ZPE***	***H_SPC***	***T.S***	***T.qh-S***	***G(T)_SPC***	***qh-G(T)_SPC***	***ΔG***
**TS**	-507.83	-537.193454	-538.059801	0.179073	-537.001645	0.054635	0.051442	-537.05628	-537.053087	**13.0**
**P**		-537.246618	-538.112652	0.182276	-537.052072	0.052041	0.049868	-537.104112	-537.101939	-17.7
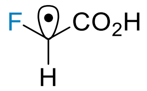		-327.245352	-327.683661	0.040676	-327.198785	0.03447	0.034471	-327.233254	-327.233256	0
**TS**	-489.94	-636.329895	-637.310874	0.172181	-636.144324	0.054717	0.05229	-636.199041	-636.196614	**13.4**
**P**		-636.384093	-637.361898	0.175071	-636.196054	0.053709	0.051518	-636.249763	-636.247572	-18.5
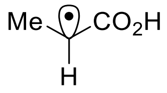		-267.359673	-267.750034	0.074575	-267.278246	0.036816	0.036404	-267.315061	-267.31465	0
**TS**	-527.82	-576.444438	-577.376434	0.206916	-576.223362	0.056213	0.053728	-576.279576	-576.27709	**14.0**
**P**		-576.490601	-577.420886	0.209886	-576.267001	0.056291	0.05331	-576.323292	-576.32031	-13.1
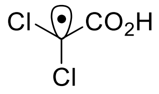		-1146.385562	-1147.638814	0.030648	-1146.347718	0.039239	0.039002	-1146.386958	-1146.38672	0
**TS**	-464.55	-1455.469026	-1457.263421	0.16139	-1455.292776	0.059361	0.056361	-1455.352137	-1455.349137	**14.0**
**P**		-1455.51634	-1457.305814	0.163596	-1455.338157	0.058447	0.055579	-1455.396603	-1455.393736	-14.0
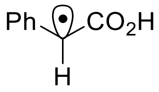		-458.759148	-459.495234	0.127841	-458.621865	0.04436	0.043437	-458.666225	-458.665302	0
**TS**	-482.38	-767.84492	-769.122567	0.259731	-767.568431	0.061589	0.058983	-767.63002	-767.627414	**14.2**
**P**		-767.879532	-769.156679	0.261878	-767.600669	0.065359	0.060808	-767.666028	-767.661477	-7.2
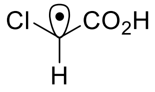		-687.251011	-688.039411	0.03925	-687.205632	0.035783	0.035731	-687.241414	-687.241362	0
**TS**	-463.27	-996.334106	-997.664646	0.170861	-996.149563	0.056849	0.053756	-996.206411	-996.203319	**14.3**
**P**		-996.383984	-997.711509	0.173411	-996.197087	0.056571	0.053529	-996.253658	-996.250616	-15.4
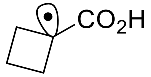		-344.610136	-345.1364	0.10931	-344.492765	0.04039	0.039881	-344.533155	-344.532646	0
**TS**	-506.21	-653.694481	-654.76082	0.240725	-653.438494	0.058755	0.056165	-653.497249	-653.494658	**14.3**

		DLPNO-(U)CCSD(T)/cc-pVTZ,SMD(MeCN)//(U)M062X-D3/Def2TZVP,SMD(MeCN)								
		In Hartree								In kcal/mol
**Species**	**Im. Freq.**	***E_SPC***	***E***	***ZPE***	***H_SPC***	***T.S***	***T.qh-S***	***G(T)_SPC***	***qh-G(T)_SPC***	***ΔG***
**P**		-653.742982	-654.806362	0.243644	-653.484304	0.058427	0.055586	-653.542731	-653.53989	-14.1
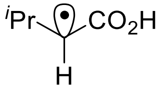		-345.846881	-346.36636	0.129962	-345.707505	0.043213	0.042414	-345.750718	-345.749919	0
**TS**	-509.89	-654.930066	-655.990651	0.261677	-654.651626	0.0625	0.059246	-654.714127	-654.710872	**14.9**
**P**		-654.978692	-656.037502	0.264788	-654.697427	0.061142	0.058432	-654.75857	-654.75586	-13.3
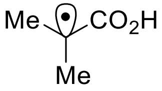		-306.609629	-307.066116	0.102183	-306.499217	0.039898	0.039665	-306.539115	-306.538883	0
**TS**	-533.00	-615.692949	-616.689664	0.234101	-615.443304	0.058761	0.056413	-615.502065	-615.499717	**15.0**
**P**		-615.737121	-616.730913	0.236924	-615.485164	0.057709	0.055096	-615.542873	-615.54026	-10.4

1.4. Linear regressions

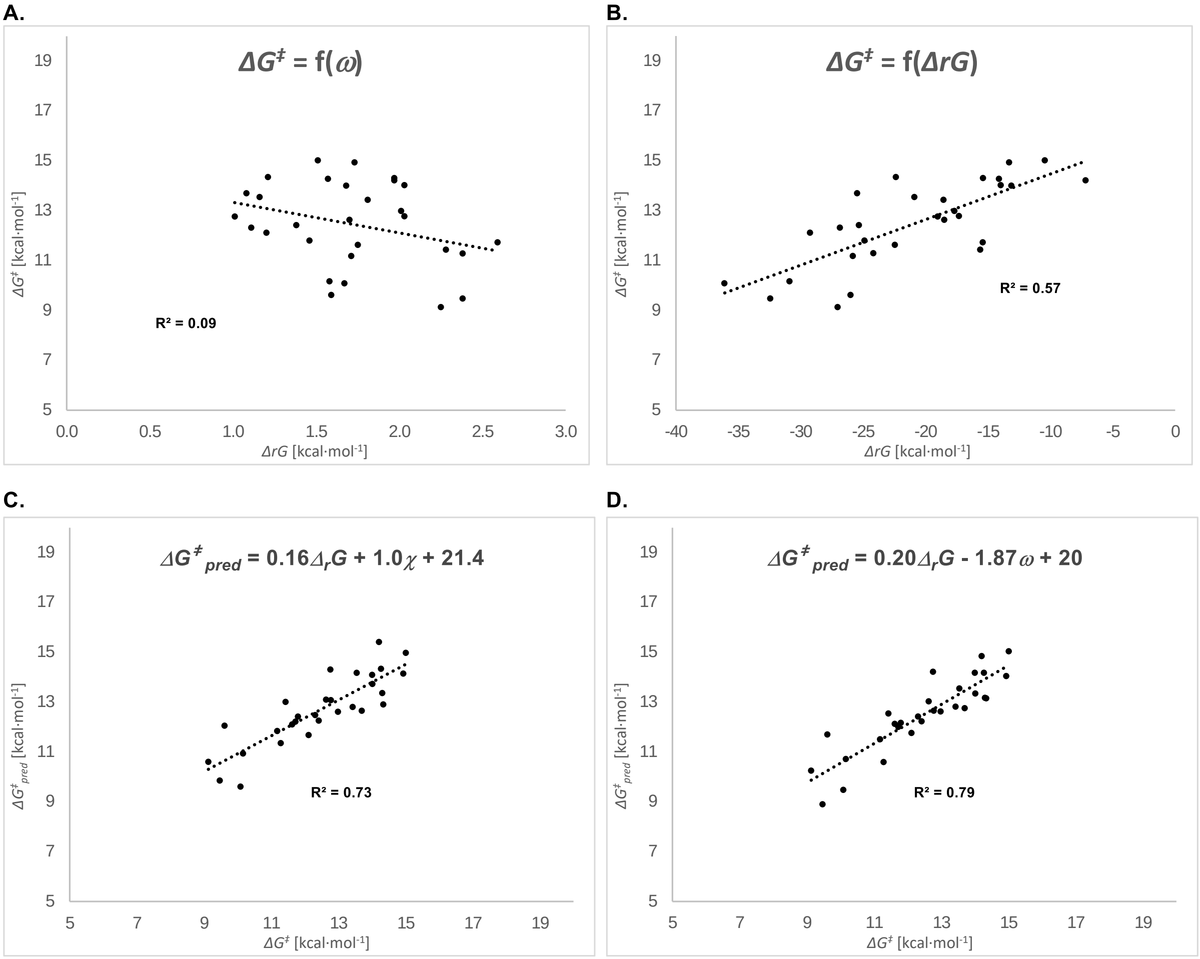

Figure S1. A) Initial correlation between the computed activation barrier ($\Delta G^{\ddagger}_{\mathrm{pred}}$) and the global electrophilicity index. **B)** Bell-Evans-Polanyi plot. **C)** Plot of the predicted activation barrier ($\Delta G^{\ddagger}_{\mathrm{pred}}$) obtained from a modified Roberts-Steel relationship (containing the Gibbs-free energy of the reaction and the electronegativity of the radical), against those calculated ($\Delta G^{\ddagger}$). **D)** Plot of the predicted activation barrier ($\Delta G^{\ddagger}_{\mathrm{pred}}$) obtained from a modified Roberts-Steel relationship (containing the reaction Gibbs-free energy of the reaction and the global electrophilicity index of the radical), against those calculated ($\Delta G^{\ddagger}$).

1.5. Decarbonylation *vs* radical addition

Table S3. Computed energies at the DLPNO-(U)CCSD(T)/cc-pVTZ,SMD(MeCN)//(U)M062X-D3/Def2TZVP,SMD(MeCN) level of theory.

		DLPNO-(U)CCSD(T)/cc-pVTZ,SMD(MeCN)//(U)M062X-D3/Def2TZVP,SMD(MeCN)								
		In Hartree								In kcal/mol
**Species**	**Im. Freq.**	***E_SPC***	***E***	***ZPE***	***H_SPC***	***T.S***	***T.qh-S***	***G(T)_SPC***	***qh-G(T)_SPC***	***ΔG***
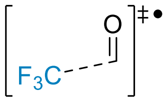	-258.79	-450.346543	-450.912756	0.018009	-450.321562	0.038427	0.038027	-450.359989	-450.35959	**12.0**

Table S4. Computed energies at the DLPNO-(U)CCSD(T)/cc-pVTZ,SMD(EtOAc)//(U)M062X-D3/Def2TZVP,SMD(EtOAc) level of theory.

		DLPNO-(U)CCSD(T)/cc-pVTZ,SMD(EtOAc)//(U)M062X-D3/Def2TZVP,SMD(EtOAc)								
		In Hartree								In kcal/mol
**Species**	**Im. Freq.**	***E_SPC***	***E***	***ZPE***	***H_SPC***	***T.S***	***T.qh-S***	***G(T)_SPC***	***qh-G(T)_SPC***	***ΔG***
**Styrene**		-309.083796	-309.626791	0.13034	-308.945718	0.03874	0.038482	-308.984458	-308.984201	
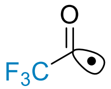		-450.370375	-450.934142	0.020832	-450.343169	0.036563	0.036275	-450.379732	-450.379445	
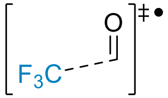	-260.45	-450.347529	-450.913528	0.018026	-450.322492	0.038693	0.038119	-450.361185	-450.3606	**11.8**
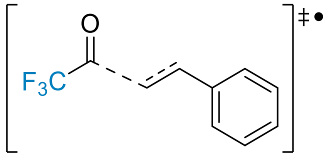	-175.14	-759.458401	-760.567398	0.152513	-759.292101	0.056087	0.053316	-759.348189	-759.345418	**11.4**

1.6. Coordinates (xyz)

1.6.1. Philicity parameters

CH(CH$_2$Cl)COOH-rad

C	1.224867000	-4.033383000	-0.373906000
O	1.794642000	-3.146751000	0.231360000
C	0.413165000	-5.056399000	0.257899000
H	0.300728000	-4.968636000	1.330538000
O	1.301166000	-4.164347000	-1.726222000
H	1.851391000	-3.437824000	-2.052004000
C	-0.328528000	-6.086616000	-0.479851000
H	0.055066000	-6.254781000	-1.480690000
H	-0.416258000	-7.018206000	0.073733000
Cl	-2.073971000	-5.504723000	-0.708367000

CHBrCOOH-rad

C	1.220278000	-4.060100000	-0.378699000
O	1.948296000	-3.290463000	0.221573000
C	0.351241000	-4.982470000	0.315009000
H	0.350106000	-4.985839000	1.394069000
Br	-0.785806000	-6.185375000	-0.524995000
O	1.159990000	-4.123587000	-1.728859000
H	1.781854000	-3.464538000	-2.069118000

CHMeCOOH-rad

C	1.248259000	-4.035373000	-0.370073000
O	1.859667000	-3.170633000	0.235394000
C	0.341526000	-4.975008000	0.242829000
H	0.234773000	-4.860149000	1.314777000
O	1.375322000	-4.190804000	-1.722183000
H	1.999991000	-3.514003000	-2.018452000
C	-0.411998000	-6.033136000	-0.470900000
H	-0.211627000	-6.025877000	-1.540900000
H	-0.158716000	-7.023790000	-0.070987000
H	-1.491501000	-5.912150000	-0.312101000

CCl$_2$COOH-rad

C	1.237447000	-4.066457000	-0.358425000
O	1.983349000	-3.303822000	0.214039000
C	0.365260000	-5.015141000	0.328815000
Cl	-0.677916000	-6.079000000	-0.505839000
O	1.117865000	-4.135970000	-1.704580000
H	1.726826000	-3.478371000	-2.070670000
Cl	0.382961000	-5.067676000	2.030420000

CHPhCOOH-rad

C	1.275886000	-3.905817000	-0.258766000
O	1.840605000	-3.164342000	0.525669000
C	0.519600000	-5.070309000	0.159862000
H	0.527543000	-5.188364000	1.236452000
O	1.326749000	-3.674437000	-1.603504000
H	1.859815000	-2.874233000	-1.717392000
C	-0.195991000	-6.032814000	-0.596769000
C	-0.316584000	-6.034887000	-2.013642000
C	-0.844635000	-7.078157000	0.119874000
C	-1.041996000	-7.024266000	-2.655262000
C	-1.565232000	-8.059864000	-0.533735000
C	-1.669772000	-8.040289000	-1.928331000
H	0.163881000	-5.256075000	-2.585184000
H	-0.764895000	-7.094266000	1.200973000
H	-1.122469000	-7.008451000	-3.736000000
H	-2.049026000	-8.844433000	0.036055000
H	-2.234457000	-8.808774000	-2.442685000

CHClCOOH-rad

C	1.212363000	-4.068541000	-0.380702000
O	1.941394000	-3.302031000	0.221318000
C	0.340862000	-4.991820000	0.309360000
H	0.334765000	-5.002315000	1.388445000
Cl	-0.697479000	-6.086005000	-0.480561000
O	1.148957000	-4.132658000	-1.730649000
H	1.771375000	-3.474989000	-2.072688000

cbutCOOH-rad

C	1.100663000	-4.001837000	-0.291666000
O	1.815450000	-3.215146000	0.307689000
O	0.866525000	-3.896167000	-1.635766000
H	1.360392000	-3.122365000	-1.942324000
C	-0.577305000	-6.738284000	1.248914000
C	-0.486325000	-6.189978000	-0.213307000
C	0.425119000	-5.117405000	0.302879000
C	0.392667000	-5.603371000	1.718076000
H	-0.188873000	-7.748073000	1.378601000
H	-1.574139000	-6.683968000	1.685763000
H	-1.423985000	-5.828010000	-0.648666000
H	-0.028494000	-6.865119000	-0.944721000
H	-0.017315000	-4.889152000	2.440055000
H	1.360629000	-5.937619000	2.107185000

CH^{i}PrCOOH-rad

C	1.059448000	-3.783576000	-0.296530000
O	1.805503000	-3.017040000	0.289845000
C	0.821986000	-5.154029000	0.084952000
H	1.389875000	-5.490515000	0.945320000
O	0.358707000	-3.393977000	-1.405104000
H	0.589456000	-2.467792000	-1.563880000
C	-0.056749000	-6.105993000	-0.651229000
H	-0.868111000	-5.534693000	-1.112836000
C	0.737192000	-6.778104000	-1.798351000
H	1.577152000	-7.355116000	-1.401516000
H	0.084533000	-7.459715000	-2.350832000
H	1.126057000	-6.033266000	-2.494727000
C	-0.650888000	-7.157792000	0.295196000
H	-1.236434000	-6.691419000	1.091172000
H	-1.305627000	-7.840540000	-0.252044000
H	0.140379000	-7.753638000	0.760949000

CMe$_2$COOH-rad

C	1.298048000	-4.078103000	-0.348587000
O	2.005885000	-3.258092000	0.214961000
C	0.471185000	-5.064408000	0.315341000
O	1.228083000	-4.131649000	-1.714680000

H	1.819721000	-3.439824000	-2.041794000
C	-0.376465000	-6.027724000	-0.442432000
H	-0.075495000	-6.113079000	-1.484808000
H	-0.348823000	-7.016779000	0.026933000
H	-1.427998000	-5.705824000	-0.424007000
C	0.442094000	-5.080487000	1.803231000
H	-0.587917000	-4.981381000	2.169080000
H	0.810903000	-6.041836000	2.184329000
H	1.048998000	-4.280297000	2.223085000

CF_2CO_2H-rad

C	-2.519751000	0.006826000	-0.049417000
F	-1.796133000	0.366604000	-1.081825000
F	-3.532953000	-0.758236000	-0.359550000
C	-2.258397000	0.402238000	1.306553000
O	-1.161306000	1.193843000	1.392295000
O	-2.946331000	0.058874000	2.245610000
H	-1.048861000	1.417763000	2.327386000

CHFCOOH-rad

C	1.224858000	-4.055743000	-0.367427000
O	1.970480000	-3.290497000	0.216939000
C	0.356953000	-4.959436000	0.340101000
H	0.319185000	-5.009154000	1.418354000
F	-0.448048000	-5.784283000	-0.315466000
O	1.127949000	-4.142183000	-1.713663000
H	1.747868000	-3.497332000	-2.084189000

CH_2COOH-rad

C	1.224292000	-4.054429000	-0.342649000
O	1.992966000	-3.285492000	0.205087000
C	0.338500000	-4.964574000	0.341608000
H	0.351421000	-4.969713000	1.421528000
H	-0.319201000	-5.622607000	-0.208533000
O	1.121589000	-4.138623000	-1.699723000
H	1.747343000	-3.499108000	-2.069398000

CF_2CO_2Li-rad

C	-2.446645000	-0.094950000	-0.039783000
F	-1.868348000	0.362039000	-1.133624000
F	-3.580664000	-0.716148000	-0.298631000
C	-2.154443000	0.460942000	1.277722000
O	-1.151222000	1.230508000	1.399890000
O	-2.897443000	0.129445000	2.252754000
Li	-1.729119000	1.266461000	3.182654000

$CHFCO_2Li$-rad

C	1.169796000	-4.108405000	-0.533871000
O	1.966886000	-3.290135000	0.043069000
C	0.363687000	-4.952526000	0.326803000
H	0.392397000	-4.934065000	1.407126000
F	-0.485155000	-5.825655000	-0.210046000
O	1.080862000	-4.187452000	-1.799878000
Li	2.379295000	-2.850981000	-1.725302000

CH_2CO_2Li-rad

C	1.161078000	-4.116746000	-0.541586000
O	1.989717000	-3.284631000	-0.031320000
C	0.351276000	-4.951796000	0.332403000
H	0.455499000	-4.863030000	1.405262000
H	-0.348965000	-5.655025000	-0.097558000
O	1.036284000	-4.222807000	-1.811311000
Li	2.340224000	-2.900692000	-1.813113000

CF_2CO_2Na-rad

C	-2.441201000	-0.154503000	-0.082243000
F	-1.910578000	0.358202000	-1.183800000
F	-3.617386000	-0.711734000	-0.334355000
C	-2.149315000	0.431807000	1.241488000
O	-1.147485000	1.199093000	1.322646000
O	-2.903585000	0.094687000	2.198953000
Na	-1.658334000	1.420744000	3.478293000

$CHFCO_2Na$-rad

C	1.143100000	-4.135042000	-0.512476000
O	1.928754000	-3.322427000	0.080544000
C	0.345896000	-4.982310000	0.362766000
H	0.383115000	-4.961687000	1.442871000
F	-0.508747000	-5.867646000	-0.157331000
O	1.026222000	-4.237792000	-1.770576000
Na	2.549429000	-2.642314000	-1.937898000

CH_2CO_2Na-rad

C	1.135440000	-4.143030000	-0.516693000
O	1.957436000	-3.317814000	0.006054000
C	0.327961000	-4.976102000	0.372443000
H	0.438360000	-4.881877000	1.444574000
H	-0.376193000	-5.682898000	-0.046186000
O	0.983428000	-4.277061000	-1.777326000
Na	2.518682000	-2.715942000	-2.040089000

CF_2CO_2K-rad

C	-2.446321000	-0.186568000	-0.126488000
F	-1.935189000	0.346836000	-1.231722000
F	-3.637297000	-0.722150000	-0.374462000
C	-2.153903000	0.413005000	1.201836000
O	-1.154505000	1.179763000	1.270713000
O	-2.910351000	0.071594000	2.152519000
K	-1.590318000	1.535816000	3.748586000

$CHFCO_2K$-rad

C	1.118992000	-4.162281000	-0.487451000
O	1.903965000	-3.354086000	0.109334000
C	0.314257000	-5.003627000	0.393166000
H	0.347540000	-4.978421000	1.473715000
F	-0.545690000	-5.891010000	-0.121898000
O	0.998807000	-4.276308000	-1.742449000
K	2.729897000	-2.483486000	-2.116517000

CH_2CO_2K-rad

C	1.110005000	-4.169258000	-0.490179000
O	1.931263000	-3.344526000	0.031494000
C	0.304269000	-5.000699000	0.407363000

H	0.417874000	-4.902510000	1.479381000
H	-0.401814000	-5.710329000	-0.004327000
O	0.948681000	-4.312905000	-1.747310000
K	2.674835000	-2.554498000	-2.233643000

1.6.2. Reactivity scale

CF_3-rad

C	0.166687000	-5.032573000	0.413563000
F	-0.709860000	-5.955056000	0.092068000
F	1.022389000	-5.479956000	1.301630000
F	0.783159000	-4.574005000	-0.650367000

CF_3-TS

C	-2.416073000	-0.147856000	0.194745000
F	-2.794970000	0.621937000	1.196667000
F	-1.695773000	-1.156672000	0.646463000
F	-3.473191000	-0.599854000	-0.455221000
C	-0.963633000	1.159973000	-1.282725000
H	-0.978247000	0.291205000	-1.931334000
H	-1.774091000	1.869122000	-1.400707000
C	0.085210000	1.455642000	-0.498117000
C	1.434168000	-0.640257000	-0.861774000
C	1.275506000	0.627035000	-0.286481000
C	2.296667000	1.117264000	0.532841000
C	3.444227000	0.373899000	0.767995000
C	3.590001000	-0.879770000	0.188601000
C	2.579510000	-1.382618000	-0.626097000
H	0.657010000	-1.051406000	-1.494380000
H	4.223420000	0.772481000	1.405809000
H	2.686205000	-2.361433000	-1.077674000
H	4.483006000	-1.464285000	0.371958000
H	2.182217000	2.095108000	0.987136000
H	0.064531000	2.382381000	0.067838000

CF_3-P

C	-2.470942000	-0.105728000	-0.085829000
F	-3.370877000	-1.092346000	-0.214815000
F	-2.720938000	0.773393000	-1.070322000
F	-2.753721000	0.525016000	1.066133000
C	-1.065021000	-0.627676000	-0.125416000
H	-0.979537000	-1.375910000	0.669801000
H	-0.951361000	-1.161421000	-1.075552000
C	-0.056521000	0.458926000	0.024618000
C	1.866217000	-1.099603000	-0.038391000
C	1.333082000	0.206844000	0.062329000
C	2.243900000	1.281180000	0.203412000
C	3.605013000	1.058652000	0.240191000
C	4.110901000	-0.237824000	0.138248000
C	3.232200000	-1.309691000	-0.000673000
H	1.197153000	-1.944587000	-0.146678000
H	4.284094000	1.895533000	0.348325000
H	3.620696000	-2.317686000	-0.080297000
H	5.179224000	-0.409784000	0.166635000
H	1.852072000	2.288822000	0.282512000
H	-0.397433000	1.484343000	0.098398000

CF_2H-rad

C	-1.214050000	1.178472000	-0.062176000
H	-1.557907000	0.568662000	-0.893096000
F	-1.728639000	2.400232000	-0.020096000
F	0.103847000	1.231053000	0.077894000

CF_2H-TS

C	-2.828636000	0.370052000	0.048598000
H	-3.605581000	0.818505000	0.663503000
C	-1.390103000	-0.937253000	1.399542000
H	-1.290785000	-0.061687000	2.030615000
H	-2.227048000	-1.591648000	1.610824000
C	-0.401218000	-1.347685000	0.581209000
C	1.787317000	-1.198483000	-0.551643000
C	0.827884000	-0.612064000	0.281149000
C	1.088036000	0.667003000	0.790699000
C	2.266964000	1.325675000	0.482787000
C	3.214708000	0.726821000	-0.342334000
C	2.968903000	-0.538648000	-0.858932000
H	1.597388000	-2.185635000	-0.958373000
H	2.448319000	2.314710000	0.885781000
H	3.697747000	-1.013060000	-1.504703000
H	4.134506000	1.245709000	-0.581417000
H	0.361270000	1.154398000	1.429054000
H	-0.516943000	-2.291919000	0.057505000
F	-3.293095000	-0.468699000	-0.875136000
F	-2.021266000	1.259134000	-0.526094000

CF_2H-P

C	-2.639162000	0.108450000	0.315708000
H	-3.481332000	0.495233000	0.888577000
C	-1.628568000	-0.643227000	1.150191000
H	-1.295172000	0.038713000	1.934414000
H	-2.174308000	-1.456272000	1.634482000
C	-0.493663000	-1.184030000	0.348829000
C	1.762159000	-1.178340000	-0.598766000
C	0.749010000	-0.535384000	0.154900000
C	1.044237000	0.743138000	0.685548000
C	2.277680000	1.330474000	0.475444000
C	3.259959000	0.676999000	-0.265861000
C	2.989883000	-0.581850000	-0.802297000
H	1.557021000	-2.157693000	-1.016383000
H	2.478977000	2.310327000	0.891481000
H	3.747505000	-1.096335000	-1.380889000
H	4.223770000	1.143411000	-0.425534000
H	0.295247000	1.276585000	1.256395000
H	-0.633010000	-2.147402000	-0.126166000
F	-3.135422000	-0.695241000	-0.673909000
F	-2.047200000	1.160197000	-0.326628000

CF_2Me-rad

C	-1.228215000	1.165269000	-0.078252000
F	-1.711238000	2.411027000	-0.029431000
F	0.095571000	1.218620000	0.102120000
C	-1.688581000	0.340520000	-1.214899000
H	-1.278814000	-0.663643000	-1.122810000
H	-1.349321000	0.783196000	-2.158218000
H	-2.775894000	0.291031000	-1.212638000

CF_2Me-TS

C	-2.375100000	-0.579783000	0.268338000
C	-1.000086000	0.150708000	-1.515043000
H	-0.814554000	-0.897328000	-1.721415000
H	-1.888645000	0.580678000	-1.961375000
C	-0.058926000	0.951179000	-0.977400000

C	1.596000000	-0.828868000	-0.327032000
C	1.229793000	0.519449000	-0.434871000
C	2.141341000	1.487578000	0.001403000
C	3.379113000	1.127448000	0.514390000
C	3.731006000	-0.212968000	0.607920000
C	2.831625000	-1.187985000	0.185989000
H	0.907349000	-1.603915000	-0.641368000
H	4.068964000	1.894677000	0.844360000
H	3.095762000	-2.235875000	0.261399000
H	4.695543000	-0.499047000	1.008417000
H	1.868961000	2.534830000	-0.068928000
H	-0.261442000	2.015382000	-0.900578000
F	-2.958909000	0.547414000	0.698019000
F	-1.512076000	-0.981532000	1.210890000
C	-3.303741000	-1.621546000	-0.221193000
H	-2.732458000	-2.478482000	-0.574770000
H	-3.970393000	-1.941597000	0.587274000
H	-3.899910000	-1.220907000	-1.039718000

CF$_2$Me-P

C	-2.143468000	-0.513401000	0.000032000
C	-1.230285000	-0.124005000	-1.151685000
H	-0.826215000	-1.050418000	-1.563104000
H	-1.868494000	0.326199000	-1.914952000
C	-0.158701000	0.828829000	-0.747622000
C	1.580856000	-0.877484000	-0.236363000
C	1.141049000	0.465293000	-0.323193000
C	2.061834000	1.477502000	0.044584000
C	3.336279000	1.165693000	0.471614000
C	3.747590000	-0.164948000	0.549644000
C	2.860054000	-1.177908000	0.191701000
H	0.908001000	-1.683169000	-0.499905000
H	4.018872000	1.960093000	0.748143000
H	3.172826000	-2.213435000	0.251467000
H	4.747384000	-0.409248000	0.885121000
H	1.745375000	2.512859000	-0.013250000
H	-0.401974000	1.884337000	-0.754595000
F	-2.662268000	0.629570000	0.566537000
F	-1.386665000	-1.078064000	1.001591000
C	-3.268187000	-1.442763000	-0.349381000
H	-2.864350000	-2.370707000	-0.752395000
H	-3.850138000	-1.658657000	0.546633000
H	-3.909294000	-0.976226000	-1.096165000

CF$_2$Ph-rad

C	-1.131300000	1.163389000	-0.147565000
F	-0.981388000	2.470592000	-0.253828000
F	-0.359209000	0.677961000	0.806941000
C	-1.591654000	0.370591000	-1.217086000
C	-1.500002000	-1.034261000	-1.148876000
C	-1.997288000	-1.804105000	-2.182044000
C	-2.592726000	-1.205879000	-3.290767000
C	-2.685994000	0.182647000	-3.359995000
C	-2.195489000	0.973230000	-2.339122000
H	-1.039054000	-1.501218000	-0.288364000
H	-1.919980000	-2.882850000	-2.126240000
H	-2.980283000	-1.817643000	-4.095055000
H	-3.145185000	0.651252000	-4.221458000
H	-2.269498000	2.051467000	-2.395863000

CF$_2$Ph-TS

C	-1.250299000	-0.134379000	0.523882000
C	-0.244333000	0.239970000	-1.519645000
H	0.382227000	-0.627887000	-1.346369000
H	-1.195842000	0.058333000	-2.005818000
C	0.239719000	1.497847000	-1.432750000
C	1.911575000	3.233690000	-0.914330000
C	1.551553000	1.880914000	-0.915456000
C	2.473588000	0.950293000	-0.415720000
C	3.708008000	1.362251000	0.058051000
C	4.053376000	2.711118000	0.047958000
C	3.148487000	3.645465000	-0.439717000
H	1.206424000	3.964077000	-1.295286000
H	4.406851000	0.628999000	0.442007000
H	3.405709000	4.697677000	-0.449626000
H	5.019155000	3.029596000	0.419895000
H	2.221558000	-0.103126000	-0.393298000
H	-0.399636000	2.323600000	-1.730529000
F	-2.208188000	0.788695000	0.509588000
F	-0.283424000	0.246113000	1.353437000
C	-1.638748000	-1.519892000	0.521022000
C	-2.887561000	-1.896810000	0.011052000
C	-0.728771000	-2.500879000	0.935974000
C	-3.223116000	-3.238120000	-0.059439000
C	-1.078986000	-3.837879000	0.859470000
C	-2.324231000	-4.212189000	0.363773000
H	-3.584908000	-1.138889000	-0.322639000
H	0.241902000	-2.208197000	1.316094000
H	-4.192514000	-3.527469000	-0.445391000
H	-0.377568000	-4.593535000	1.190393000
H	-2.592259000	-5.259474000	0.305986000

CF$_2$Ph-P

C	-1.060834000	-0.181561000	0.236779000
C	-0.307900000	0.122416000	-1.052506000
H	0.490213000	-0.617400000	-1.134649000
H	-1.013618000	-0.063554000	-1.865718000
C	0.204455000	1.518937000	-1.115254000
C	1.887642000	3.280240000	-0.910708000
C	1.513179000	1.922506000	-0.758354000
C	2.489176000	1.029926000	-0.253614000
C	3.756509000	1.475969000	0.070451000
C	4.103146000	2.815819000	-0.092118000
C	3.156335000	3.714039000	-0.584767000
H	1.155106000	3.980562000	-1.295776000
H	4.485577000	0.774352000	0.457455000
H	3.416148000	4.757817000	-0.713171000
H	5.097937000	3.156540000	0.164954000
H	2.244682000	-0.015022000	-0.110429000
H	-0.478382000	2.285731000	-1.460353000
F	-2.100850000	0.701873000	0.366293000
F	-0.244447000	0.075343000	1.307418000
C	-1.576512000	-1.592710000	0.324829000
C	-2.855600000	-1.898958000	-0.123428000
C	-0.739992000	-2.598992000	0.795601000
C	-3.302447000	-3.213424000	-0.086688000
C	-1.190826000	-3.911097000	0.830607000
C	-2.472026000	-4.219517000	0.390116000
H	-3.502119000	-1.114596000	-0.496360000
H	0.259475000	-2.356172000	1.135348000
H	-4.301431000	-3.450740000	-0.430112000
H	-0.540127000	-4.692630000	1.202131000
H	-2.822618000	-5.243595000	0.418134000

CH_2F-rad

C	-0.919362000	1.013091000	-0.495421000
H	-1.811598000	0.433701000	-0.306908000
F	-0.088629000	1.096197000	0.558032000
H	-0.868261000	1.859052000	-1.165403000

CH_2F-TS

C	-2.978358000	-0.054641000	-0.790832000
H	-3.315247000	-1.073503000	-0.930031000
H	-2.263283000	0.394594000	-1.468266000
C	-1.738159000	-0.213301000	1.187153000
H	-1.723333000	0.869587000	1.147065000
H	-2.604150000	-0.663611000	1.656051000
C	-0.627918000	-0.952065000	0.971129000
C	1.718859000	-1.340518000	0.315338000
C	0.640844000	-0.456144000	0.442337000
C	0.829910000	0.874762000	0.044420000
C	2.053398000	1.300244000	-0.447539000
C	3.117726000	0.410536000	-0.559693000
C	2.943508000	-0.913680000	-0.176906000
H	1.586016000	-2.375073000	0.612035000
H	2.178828000	2.333248000	-0.748731000
H	3.762739000	-1.617060000	-0.263100000
H	4.072115000	0.748376000	-0.943726000
H	0.013802000	1.583444000	0.117768000
H	-0.668975000	-2.021522000	1.154229000
F	-3.980865000	0.785962000	-0.452143000

CH_2F-P

C	-2.633662000	-0.054041000	-0.447453000
H	-2.945868000	-1.035247000	-0.807281000
H	-1.994698000	0.424224000	-1.191319000
C	-1.943098000	-0.150039000	0.902581000
H	-1.719512000	0.863411000	1.246371000
H	-2.638326000	-0.600677000	1.612827000
C	-0.703580000	-0.973576000	0.806530000
C	1.661808000	-1.356067000	0.289959000
C	0.544048000	-0.487708000	0.347876000
C	0.738888000	0.854213000	-0.059964000
C	1.976526000	1.290223000	-0.494817000
C	3.062038000	0.417765000	-0.540891000
C	2.892821000	-0.909279000	-0.144833000
H	1.534094000	-2.387877000	0.597655000
H	2.101086000	2.321361000	-0.802890000
H	3.731665000	-1.593875000	-0.177855000
H	4.028154000	0.766869000	-0.882260000
H	-0.091424000	1.549365000	-0.035143000
H	-0.764892000	-2.022150000	1.071998000
F	-3.786084000	0.729528000	-0.325034000

CH_3-rad

C	-0.965034000	1.095865000	-0.407303000
H	-1.807608000	0.422472000	-0.369128000
H	-0.885056000	1.815641000	-1.207677000
H	-0.205582000	1.054419000	0.358668000

CH_3-TS

C	-3.361271000	-0.771334000	1.049433000
H	-2.506294000	-1.353618000	1.364497000
H	-4.163418000	-1.292556000	0.544130000
H	-3.625123000	0.090199000	1.646635000
C	-2.420573000	0.282977000	-0.806130000
H	-2.326655000	-0.705014000	-1.239801000
H	-3.354674000	0.798954000	-0.986998000
C	-1.340247000	0.976578000	-0.378224000
C	0.305965000	-0.922776000	-0.363157000
C	0.002687000	0.435621000	-0.190942000
C	1.038529000	1.297652000	0.191122000
C	2.329581000	0.828627000	0.380708000
C	2.615810000	-0.519234000	0.199152000
C	1.596062000	-1.390682000	-0.171782000
H	-0.474900000	-1.619059000	-0.644103000
H	3.114511000	1.515457000	0.673173000
H	1.808746000	-2.444044000	-0.309018000
H	3.622216000	-0.890154000	0.347579000
H	0.818551000	2.349479000	0.336560000
H	-1.467550000	2.015793000	-0.090136000

CH_3-P

C	-3.141262000	-0.589702000	0.719857000
H	-2.401347000	-1.255545000	1.167900000
H	-4.028211000	-1.177201000	0.476149000
H	-3.419664000	0.156490000	1.466388000
C	-2.574541000	0.086334000	-0.536731000
H	-2.332283000	-0.682671000	-1.275108000
H	-3.347316000	0.718857000	-0.977519000
C	-1.375953000	0.917541000	-0.232611000
C	0.261356000	-0.951483000	-0.286253000
C	-0.059269000	0.415902000	-0.101006000
C	1.002803000	1.293198000	0.231809000
C	2.297255000	0.834372000	0.363821000
C	2.590162000	-0.516914000	0.173862000
C	1.562081000	-1.400090000	-0.150444000
H	-0.522556000	-1.656433000	-0.534117000
H	3.090211000	1.527914000	0.616736000
H	1.781740000	-2.450909000	-0.297215000
H	3.605880000	-0.876512000	0.277847000
H	0.780370000	2.343845000	0.381594000
H	-1.523859000	1.973583000	-0.035178000

$CF(CF_3)_2$-rad

C	1.443840000	3.934796000	-0.316008000
F	1.915404000	4.246811000	-1.499153000
C	2.454259000	3.365105000	0.632033000
F	1.889967000	3.117746000	1.809517000
F	3.474587000	4.206768000	0.826683000
F	2.965279000	2.233415000	0.154029000
C	0.242208000	4.710441000	0.147284000
F	-0.399223000	5.214715000	-0.897815000
F	0.581723000	5.723024000	0.954562000
F	-0.594236000	3.929703000	0.826980000

$CF(CF_3)_2$-TS

C	-1.090098000	0.584787000	0.184611000
C	0.491721000	0.680581000	-1.692088000
H	0.832857000	1.606847000	-1.244481000
H	-0.310256000	0.751967000	-2.417263000
C	1.159694000	-0.477970000	-1.538859000
C	2.860633000	-1.995263000	-0.616679000
C	2.306368000	-0.712104000	-0.662029000
C	2.865074000	0.287488000	0.144350000
C	3.945939000	0.006814000	0.963208000
C	4.490381000	-1.273963000	0.995429000
C	3.943989000	-2.275051000	0.202981000

H	2.430501000	-2.775761000	-1.234241000
H	4.368104000	0.789308000	1.581514000
H	4.360301000	-3.274481000	0.224893000
H	5.336238000	-1.488807000	1.636637000
H	2.453551000	1.289082000	0.133664000
H	0.809869000	-1.351382000	-2.082185000
C	-2.025105000	1.721109000	-0.112960000
F	-2.774479000	1.458481000	-1.179565000
F	-2.852261000	1.973788000	0.912787000
F	-1.339556000	2.841077000	-0.339575000
C	-1.597198000	-0.830923000	0.189861000
F	-0.625548000	-1.659495000	0.561849000
F	-2.616398000	-1.005186000	1.048004000
F	-2.030520000	-1.196615000	-1.013315000
F	-0.294333000	0.849727000	1.216988000

CF(CF$_3$)$_2$-P

C	-0.917562000	0.550013000	-0.083102000
C	0.127366000	0.525141000	-1.202069000
H	0.714209000	1.437358000	-1.087293000
H	-0.408372000	0.596708000	-2.151423000
C	0.995916000	-0.687410000	-1.193847000
C	2.948441000	-2.016996000	-0.586559000
C	2.216714000	-0.808154000	-0.488657000
C	2.757768000	0.220270000	0.318436000
C	3.958921000	0.041335000	0.979127000
C	4.661732000	-1.156310000	0.866894000
C	4.145364000	-2.184358000	0.078669000
H	2.549073000	-2.816945000	-1.199637000
H	4.354397000	0.842287000	1.591945000
H	4.684453000	-3.119433000	-0.012863000
H	5.601524000	-1.287835000	1.387871000
H	2.229153000	1.158458000	0.428232000
H	0.664328000	-1.541561000	-1.771408000
C	-1.830549000	1.787669000	-0.207817000
F	-2.689598000	1.634873000	-1.215207000
F	-2.527092000	2.001863000	0.904271000
F	-1.109371000	2.881544000	-0.439435000
C	-1.762995000	-0.742738000	-0.033016000
F	-1.053131000	-1.734567000	0.494638000
F	-2.857176000	-0.595642000	0.714695000
F	-2.149461000	-1.109080000	-1.252703000
F	-0.308454000	0.651561000	1.141644000

perfFcp-rad

C	-1.027143000	-0.547727000	-0.080818000
C	0.505849000	-0.487564000	-0.052659000
C	-0.314503000	0.584477000	0.521802000
F	-1.621777000	-1.465565000	0.680493000
F	-1.685196000	-0.409990000	-1.230584000
F	1.193188000	-0.299020000	-1.177617000
F	1.141625000	-1.355655000	0.733709000
F	-0.355024000	1.090028000	1.723135000

perfFcp-TS

C	0.369216000	-1.938104000	0.857490000
H	0.543031000	-1.262337000	1.686465000
H	-0.342497000	-2.738170000	1.017919000
C	1.095204000	-1.891476000	-0.269153000
C	2.202257000	0.327243000	0.115226000
C	2.082082000	-0.863845000	-0.610403000
C	2.917173000	-1.062353000	-1.713310000
C	3.860643000	-0.110368000	-2.071892000
C	3.975777000	1.063555000	-1.338766000
C	3.140753000	1.279537000	-0.246406000
H	1.548624000	0.515907000	0.958942000
H	4.502941000	-0.283607000	-2.926513000
H	3.218342000	2.199138000	0.320492000
H	4.706351000	1.811920000	-1.619933000
H	2.822275000	-1.976689000	-2.288276000
H	0.936318000	-2.654223000	-1.025832000
C	-2.568503000	-0.285026000	0.854694000
C	-1.570715000	0.835916000	1.120336000
C	-1.316959000	-0.183712000	0.094463000
F	-3.740633000	0.049652000	0.307442000
F	-2.749404000	-1.264544000	1.745142000
F	-0.861033000	0.873040000	2.251908000
F	-1.934394000	2.075388000	0.777247000
F	-1.152577000	-0.013918000	-1.199232000

perfFcp-P

C	-0.119930000	-1.497602000	0.571932000
H	0.291398000	-1.022110000	1.460531000
H	-0.320358000	-2.542477000	0.828896000
C	0.799996000	-1.412644000	-0.598455000
C	2.406498000	0.234847000	0.366824000
C	1.962670000	-0.611404000	-0.678311000
C	2.741327000	-0.646040000	-1.863050000
C	3.885354000	0.113397000	-1.991646000
C	4.303542000	0.939030000	-0.948066000
C	3.554789000	0.990534000	0.226018000
H	1.844683000	0.302691000	1.289330000
H	4.460473000	0.067092000	-2.908399000
H	3.873048000	1.630754000	1.039807000
H	5.202173000	1.534219000	-1.048511000
H	2.419695000	-1.287122000	-2.675963000
H	0.545278000	-2.013852000	-1.463090000
C	-2.322052000	-0.262116000	1.343658000
C	-1.681210000	0.613885000	0.316990000
C	-1.460168000	-0.882791000	0.263001000
F	-3.641215000	-0.430244000	1.284227000
F	-1.891405000	-0.250917000	2.604017000
F	-0.669887000	1.414820000	0.649407000
F	-2.466567000	1.178807000	-0.597272000
F	-2.153165000	-1.516497000	-0.717879000

CF$_2$CO$_2$Me-rad

C	-2.556652000	0.016266000	-0.031582000
F	-1.797827000	0.334208000	-1.039017000
F	-3.529626000	-0.786905000	-0.342099000
C	-2.307338000	0.409441000	1.326931000
O	-1.242126000	1.203148000	1.415794000
C	-0.929444000	1.660277000	2.738386000
H	-0.048452000	2.285735000	2.630655000
H	-1.759627000	2.238458000	3.142314000
H	-0.716029000	0.813528000	3.389387000
O	-3.011472000	0.051781000	2.245902000

CF$_2$CO$_2$Me-TS

C	-0.962577000	1.902600000	-0.568539000
H	-0.921164000	1.546092000	-1.591924000
H	-1.799503000	2.542128000	-0.312619000
C	0.095116000	1.808510000	0.266544000
C	2.231612000	0.865534000	1.054889000

C	1.317005000	1.049640000	0.011225000
C	1.600022000	0.467490000	-1.231430000
C	2.755681000	-0.273477000	-1.417142000
C	3.652598000	-0.453690000	-0.368227000
C	3.385655000	0.119513000	0.869367000
H	2.023034000	1.310851000	2.021485000
H	2.958559000	-0.715634000	-2.384970000
H	4.079080000	-0.014497000	1.690475000
H	4.554717000	-1.033932000	-0.517312000
H	0.913948000	0.595903000	-2.059339000
H	0.024811000	2.265130000	1.249199000
C	-2.327950000	0.103297000	-0.051272000
F	-3.253352000	0.166704000	-0.986749000
C	-1.373966000	-1.009365000	-0.130057000
O	-0.728811000	-1.160317000	1.016698000
C	0.341685000	-2.115547000	1.008182000
H	-0.056947000	-3.116943000	0.848903000
H	1.057809000	-1.870605000	0.224349000
H	0.805640000	-2.045567000	1.987406000
O	-1.165101000	-1.617866000	-1.151385000
F	-2.813920000	0.450576000	1.124495000

CF_2CO_2Me-P

C	-1.130775000	1.574321000	-0.487859000
H	-0.891579000	1.428656000	-1.542661000
H	-1.740485000	2.476901000	-0.401217000
C	0.085038000	1.675314000	0.367483000
C	2.378969000	1.164059000	1.032777000
C	1.325938000	1.051228000	0.091970000
C	1.573417000	0.293506000	-1.076874000
C	2.799695000	-0.313354000	-1.281111000
C	3.818412000	-0.192287000	-0.339149000
C	3.596789000	0.552177000	0.819635000
H	2.208148000	1.739346000	1.935577000
H	2.966425000	-0.886973000	-2.184882000
H	4.383833000	0.652342000	1.557120000
H	4.774901000	-0.671345000	-0.505264000
H	0.802805000	0.183961000	-1.829653000
H	-0.006938000	2.220537000	1.298451000
C	-2.045941000	0.429305000	-0.077811000
F	-3.149471000	0.425493000	-0.866939000
C	-1.375376000	-0.948355000	-0.204716000
O	-0.781650000	-1.303507000	0.913669000
C	0.004014000	-2.505478000	0.855559000
H	-0.633146000	-3.356729000	0.621176000
H	0.781737000	-2.399903000	0.099587000
H	0.442612000	-2.617711000	1.842187000
O	-1.374662000	-1.561493000	-1.235310000
F	-2.485998000	0.610596000	1.194765000

$ClCF_2CO_2COCF_2$-rad

C	-1.632372000	-0.385553000	0.438591000
O	-1.755691000	-1.274348000	1.236008000
C	-2.694695000	0.297539000	-0.202929000
F	-3.924545000	0.018534000	0.062223000
F	-2.533538000	1.241080000	-1.067635000
O	-0.396949000	0.154082000	0.070093000
C	0.576677000	-0.695723000	-0.299230000
O	0.463372000	-1.842823000	-0.565031000
C	1.924671000	0.049984000	-0.329270000
F	1.792793000	1.252507000	-0.886523000
F	2.797651000	-0.650697000	-1.041350000
Cl	2.514187000	0.225508000	1.325492000

$ClCF_2CO_2COCF_2$-TS

C	1.321573000	1.377086000	-1.291273000
O	1.917762000	1.946518000	-2.156235000
C	1.864938000	0.452995000	-0.341452000
C	1.727303000	-1.437632000	-1.659056000
H	0.691803000	-1.262943000	-1.930343000
H	2.481020000	-1.053238000	-2.336226000
C	2.070417000	-2.338578000	-0.709347000
C	1.688092000	-3.853336000	1.188874000
C	1.156675000	-3.014612000	0.202545000
C	-0.233335000	-2.842842000	0.145354000
C	-1.058041000	-3.486902000	1.050321000
C	-0.515349000	-4.316654000	2.028581000
C	0.859956000	-4.499864000	2.093676000
H	2.762278000	-3.990092000	1.239799000
H	-2.130158000	-3.342775000	0.996972000
H	1.287343000	-5.145437000	2.850690000
H	-1.164906000	-4.817649000	2.735496000
H	-0.670749000	-2.200953000	-0.609381000
H	3.124434000	-2.551606000	-0.556715000
F	1.160704000	0.101292000	0.706632000
F	3.147830000	0.481215000	-0.100633000
O	-0.092361000	1.406645000	-1.244462000
C	-0.631598000	2.150079000	-0.275685000
O	-0.049418000	2.840895000	0.494527000
C	-2.160133000	1.968801000	-0.250481000
F	-2.657185000	1.880821000	-1.481896000
F	-2.717691000	3.008957000	0.356086000
Cl	-2.531774000	0.491711000	0.643777000

$ClCF_2CO_2COCF_2$-P

C	-1.584611000	-1.516074000	-0.392997000
O	-2.047998000	-2.225400000	-1.214139000
C	-2.380315000	-0.470951000	0.408012000
C	-2.496950000	0.860941000	-0.316684000
H	-3.079777000	0.667432000	-1.219240000
H	-3.090750000	1.498759000	0.342660000
C	-1.179367000	1.464093000	-0.670215000
C	0.832048000	2.794690000	-0.285647000
C	-0.414427000	2.304480000	0.174215000
C	-0.832631000	2.689575000	1.469299000
C	-0.044514000	3.514398000	2.249769000
C	1.181242000	3.980407000	1.778691000
C	1.612507000	3.613728000	0.504269000
H	1.166236000	2.515915000	-1.278388000
H	-0.384266000	3.798484000	3.238225000
H	2.563266000	3.973752000	0.130566000
H	1.794199000	4.622856000	2.397960000
H	-1.778540000	2.335904000	1.859117000
H	-0.776458000	1.232614000	-1.649136000
F	-1.805574000	-0.318952000	1.624354000
F	-3.610449000	-0.993485000	0.616917000
O	-0.249394000	-1.431808000	-0.076055000
C	0.635567000	-2.366395000	-0.501748000
O	0.408428000	-3.479713000	-0.817939000
C	2.048727000	-1.750905000	-0.509293000
F	2.949510000	-2.722957000	-0.552144000
F	2.259342000	-1.024242000	0.586439000
Cl	2.211522000	-0.727110000	-1.937771000

CF_3CO-rad

C	0.278081000	-5.812253000	-1.149477000
F	-0.886709000	-6.423757000	-1.286185000
C	0.243167000	-4.892944000	0.111067000
O	1.088486000	-4.822518000	0.906338000
F	1.234694000	-6.727485000	-1.073773000
F	0.496852000	-5.051302000	-2.212711000

CF_3CO-TS

C	-2.254178000	-0.343035000	-0.135599000
F	-3.256438000	-0.622191000	-0.968448000
C	-1.515343000	0.909274000	-0.677834000
O	-0.851690000	0.872618000	-1.648464000
F	-2.762976000	-0.133989000	1.069609000
F	-1.457635000	-1.409669000	-0.073696000
C	-0.446893000	1.827385000	1.172317000
H	-0.076948000	2.616083000	0.528616000
H	-1.321175000	2.057663000	1.767551000
C	0.238845000	0.686776000	1.382606000
C	1.971562000	-1.002999000	0.939755000
C	1.454716000	0.271640000	0.685413000
C	2.107771000	1.087699000	-0.245178000
C	3.244610000	0.637864000	-0.897254000
C	3.748960000	-0.631763000	-0.634442000
C	3.109307000	-1.451840000	0.286838000
H	1.467283000	-1.641693000	1.656350000
H	3.741730000	1.280176000	-1.613572000
H	3.496517000	-2.441547000	0.494852000
H	4.638089000	-0.978597000	-1.146297000
H	1.733026000	2.081854000	-0.454354000
H	-0.154839000	-0.030961000	2.096950000

CF_3CO-P

C	-2.946554000	0.098474000	-0.323488000
F	-2.890603000	1.333990000	-0.826203000
C	-1.710340000	-0.207739000	0.561439000
O	-1.892127000	-0.666482000	1.650952000
F	-4.075959000	-0.018072000	0.359869000
F	-2.996193000	-0.754665000	-1.352533000
C	-0.388343000	0.097537000	-0.071486000
H	-0.385447000	-0.361085000	-1.070387000
H	-0.375263000	1.178953000	-0.272116000
C	0.773067000	-0.327539000	0.748698000
C	2.456134000	0.442177000	-0.900822000
C	2.113266000	-0.153704000	0.335461000
C	3.170404000	-0.583613000	1.173387000
C	4.487739000	-0.426990000	0.794436000
C	4.804313000	0.162160000	-0.430079000
C	3.779979000	0.593363000	-1.269881000
H	1.674159000	0.785847000	-1.566953000
H	5.279607000	-0.763937000	1.452327000
H	4.019958000	1.052672000	-2.221228000
H	5.838658000	0.283275000	-0.725508000
H	2.927208000	-1.042043000	2.125301000
H	0.580331000	-0.787274000	1.709282000

CF_3COCH_2-rad

C	1.116257000	-4.052323000	-1.833322000
F	1.327953000	-5.258990000	-2.372356000
C	1.234895000	-4.111572000	-0.294986000
O	2.137446000	-3.490723000	0.244817000
F	1.996888000	-3.211565000	-2.358744000
F	-0.105448000	-3.659349000	-2.210954000
C	0.274094000	-4.907248000	0.378351000
H	0.329331000	-4.981424000	1.455185000
H	-0.499451000	-5.435793000	-0.160616000

CF_3COCH_2-TS

C	-2.145585000	0.609069000	0.005160000
F	-2.221848000	0.274164000	1.299196000
C	-1.481651000	-0.487853000	-0.853003000
O	-0.760437000	-0.125420000	-1.773876000
F	-3.401598000	0.830541000	-0.413982000
F	-1.492576000	1.764433000	-0.075031000
C	-1.779358000	-1.828221000	-0.507024000
H	-1.508827000	-2.600333000	-1.212529000
H	-2.524346000	-2.049532000	0.244976000
C	-0.055450000	-2.367853000	0.966762000
H	-0.446348000	-1.694208000	1.718847000
H	-0.438839000	-3.379930000	0.978619000
C	1.060525000	-2.081962000	0.255481000
C	1.339458000	0.302138000	0.984712000
C	1.751798000	-0.801784000	0.225068000
C	2.864181000	-0.656887000	-0.613604000
C	3.542213000	0.548889000	-0.695197000
C	3.119808000	1.635648000	0.061377000
C	2.017064000	1.506614000	0.900734000
H	0.483540000	0.222366000	1.643945000
H	4.398629000	0.642984000	-1.351149000
H	1.685027000	2.351637000	1.491291000
H	3.645817000	2.580083000	-0.001806000
H	3.187295000	-1.504194000	-1.207580000
H	1.465100000	-2.846273000	-0.401456000

CF_3COCH_2-P

C	-3.597706000	0.146615000	0.010254000
F	-3.843336000	-0.647954000	1.057788000
C	-2.176040000	-0.122643000	-0.549791000
O	-2.081140000	-0.583029000	-1.650853000
F	-4.533996000	-0.077280000	-0.900967000
F	-3.719910000	1.410214000	0.426885000
C	-1.047713000	0.217465000	0.368654000
H	-1.222422000	-0.300359000	1.318023000
H	-1.119541000	1.286643000	0.596424000
C	0.309625000	-0.132795000	-0.216044000
H	0.329536000	-1.198913000	-0.473771000
H	0.443744000	0.395713000	-1.166502000
C	1.421786000	0.191692000	0.719961000
C	3.790364000	0.353974000	1.342153000
C	2.789110000	0.034994000	0.393388000
C	3.212400000	-0.439365000	-0.870877000
C	4.557261000	-0.580059000	-1.160379000
C	5.525300000	-0.258519000	-0.211760000
C	5.129478000	0.208734000	1.042295000
H	3.485510000	0.716351000	2.317513000
H	4.859563000	-0.943271000	-2.135296000
H	5.876828000	0.458238000	1.785938000
H	6.576442000	-0.370969000	-0.445022000
H	2.474292000	-0.695009000	-1.621313000
H	1.176312000	0.552661000	1.712726000

$CF_2PO(OMe)2$-rad

C	-2.630526000	-0.180918000	-0.000287000
F	-3.875822000	-0.041323000	-0.419957000

F	-2.121492000	-1.325542000	-0.415726000
P	-2.240659000	0.386623000	1.673840000
O	-0.678403000	0.406936000	1.542238000
O	-2.761712000	1.864508000	1.668077000
C	0.149669000	0.446128000	2.724567000
H	1.177461000	0.408666000	2.376213000
H	-0.023620000	1.371961000	3.272716000
H	-0.066374000	-0.413570000	3.356180000
C	-2.216292000	2.858827000	0.778658000
H	-2.158776000	2.475154000	-0.241234000
H	-2.894470000	3.706527000	0.814282000
H	-1.226421000	3.154820000	1.122834000
O	-2.803209000	-0.394086000	2.775581000

$CF_2PO(OMe)2$-TS

C	0.383900000	0.958441000	1.434982000
H	0.558920000	1.958833000	1.055399000
H	-0.483697000	0.827506000	2.069187000
C	1.330126000	-0.001283000	1.378378000
C	3.531376000	-0.939451000	0.775286000
C	2.584514000	0.080988000	0.632812000
C	2.875850000	1.141299000	-0.235594000
C	4.076449000	1.180616000	-0.924587000
C	5.013132000	0.163005000	-0.765569000
C	4.735068000	-0.898068000	0.086777000
H	3.312928000	-1.770226000	1.436906000
H	4.283653000	2.006140000	-1.594468000
H	5.456475000	-1.695783000	0.213639000
H	5.949849000	0.196441000	-1.307661000
H	2.152695000	1.934822000	-0.381584000
H	1.152019000	-0.941703000	1.891970000
C	-0.847094000	0.682269000	-0.563496000
F	-0.985653000	1.913330000	-1.049520000
F	-0.009341000	-0.006406000	-1.336792000
P	-2.382675000	-0.206290000	-0.176699000
O	-1.886365000	-1.448689000	0.646543000
O	-2.970694000	0.763784000	0.900495000
C	-1.544269000	-2.688937000	-0.001350000
H	-2.261612000	-2.912275000	-0.789284000
H	-0.537531000	-2.621958000	-0.412975000
H	-1.580184000	-3.457977000	0.765568000
C	-4.343823000	0.625658000	1.321658000
H	-4.520372000	1.416014000	2.045092000
H	-5.008414000	0.742673000	0.467321000
H	-4.494676000	-0.346735000	1.789961000
O	-3.228271000	-0.518637000	-1.333961000

$CF_2PO(OMe)2$-P

C	0.086475000	1.454364000	0.866206000
H	0.639575000	2.357791000	0.594779000
H	-0.525646000	1.691085000	1.738420000
C	1.000700000	0.312105000	1.160069000
C	3.017864000	-1.040882000	0.893288000
C	2.254760000	0.098297000	0.537465000
C	2.794686000	0.965977000	-0.441018000
C	4.020968000	0.702804000	-1.021219000
C	4.752595000	-0.426143000	-0.658048000
C	4.239969000	-1.295516000	0.304785000
H	2.621041000	-1.719097000	1.640311000
H	4.414172000	1.382575000	-1.767318000
H	4.802226000	-2.175675000	0.592218000
H	5.711078000	-0.626040000	-1.119949000
H	2.244094000	1.846121000	-0.746357000
H	0.682583000	-0.406763000	1.903919000
C	-0.864311000	1.199763000	-0.292724000
F	-1.502217000	2.366574000	-0.612313000
F	-0.158262000	0.846997000	-1.409752000
P	-2.187695000	-0.080887000	-0.066043000
O	-1.495735000	-1.310028000	0.629740000
O	-2.993817000	0.581542000	1.100629000
C	-0.770516000	-2.283731000	-0.145828000
H	-1.442691000	-2.770347000	-0.850546000
H	0.054402000	-1.809412000	-0.678799000
H	-0.378526000	-3.008132000	0.562889000
C	-4.245116000	0.015540000	1.539774000
H	-4.642208000	0.694867000	2.288308000
H	-4.935243000	-0.057867000	0.700464000
H	-4.075826000	-0.966923000	1.979370000
O	-2.897724000	-0.377916000	-1.312350000

$CHFCF_2OMe$-rad

C	-0.052753000	-0.277231000	-0.092132000
F	-0.496093000	-0.848418000	1.079460000
C	1.429572000	-0.332607000	-0.183898000
F	2.067043000	0.511581000	0.619974000
O	-0.494443000	0.982299000	-0.197071000
C	-1.907452000	1.171049000	-0.015862000
H	-2.472209000	0.503175000	-0.665714000
H	-2.102611000	2.203313000	-0.291290000
H	-2.179988000	1.007280000	1.025577000
F	-0.563606000	-1.083157000	-1.065692000
H	1.952120000	-1.248327000	-0.423305000

$CHFCF_2OMe$-TS

C	-0.456202000	1.969728000	0.578426000
H	-0.256616000	2.563214000	-0.306677000
H	-1.338016000	2.232065000	1.149081000
C	0.475808000	1.154027000	1.114348000
C	2.205323000	1.314755000	-0.708457000
C	1.752850000	0.783490000	0.507328000
C	2.560032000	-0.154238000	1.160984000
C	3.776738000	-0.549527000	0.625342000
C	4.212543000	-0.013921000	-0.579972000
C	3.420585000	0.919747000	-1.242646000
H	1.603601000	2.042398000	-1.239066000
H	4.384284000	-1.278029000	1.148012000
H	3.755044000	1.340676000	-2.183129000
H	5.161252000	-0.320926000	-1.002393000
H	2.219134000	-0.574167000	2.100763000
H	0.254896000	0.663318000	2.057793000
C	-2.101777000	-0.603915000	-0.112403000
F	-0.974975000	-1.317879000	0.175731000
C	-1.750593000	0.640461000	-0.852511000
F	-0.920355000	0.403552000	-1.880117000
O	-2.798947000	-0.298116000	0.988158000
C	-3.275165000	-1.413479000	1.756727000
H	-3.669160000	-0.990305000	2.676051000
H	-2.459425000	-2.097647000	1.988295000
H	-4.066684000	-1.934451000	1.219898000
F	-2.816247000	-1.463401000	-0.923113000
H	-2.539541000	1.348958000	-1.072754000

$CHFCF_2OMe$-P

C	-0.616038000	1.435609000	0.347274000

H	-0.255209000	2.291094000	-0.226087000
H	-1.270988000	1.826286000	1.128290000
C	0.510972000	0.671952000	0.955480000
C	2.195896000	1.198229000	-0.800045000
C	1.813658000	0.573518000	0.411435000
C	2.800743000	-0.177584000	1.096788000
C	4.084454000	-0.292167000	0.603358000
C	4.439708000	0.336454000	-0.590104000
C	3.485543000	1.078940000	-1.282886000
H	1.471405000	1.773319000	-1.361779000
H	4.819128000	-0.873432000	1.147363000
H	3.753832000	1.567935000	-2.211617000
H	5.446929000	0.246394000	-0.976328000
H	2.528360000	-0.667555000	2.024830000
H	0.318566000	0.144052000	1.881761000
C	-2.185514000	-0.566163000	0.048275000
F	-1.279755000	-1.536156000	0.365419000
C	-1.496069000	0.628287000	-0.597277000
F	-0.739281000	0.125044000	-1.643612000
O	-2.876923000	-0.169655000	1.124165000
C	-3.686176000	-1.171458000	1.759497000
H	-4.054563000	-0.714212000	2.673057000
H	-3.090851000	-2.050621000	2.003189000
H	-4.523361000	-1.444099000	1.118613000
F	-2.997406000	-1.164463000	-0.873382000
H	-2.276491000	1.261011000	-1.023778000

$CHFCO_2Me$-rad

C	-2.523973000	0.034959000	-0.053859000
F	-3.542389000	-0.765920000	-0.294390000
C	-2.310156000	0.421356000	1.319155000
O	-1.250150000	1.228164000	1.424310000
C	-0.933016000	1.660999000	2.750632000
H	-0.057713000	2.296634000	2.653324000
H	-1.764034000	2.223431000	3.175321000
H	-0.708400000	0.804103000	3.385057000
O	-3.000468000	0.060484000	2.247034000
H	-1.929505000	0.325501000	-0.906148000

$CHFCO_2Me$-TS

C	-1.050692000	2.033086000	0.988389000
H	-1.237617000	2.240399000	-0.059447000
H	-1.666610000	2.569006000	1.699905000
C	0.093932000	1.436788000	1.403755000
C	1.030117000	0.866651000	-0.854626000
C	1.072463000	0.782400000	0.544026000
C	2.083025000	0.010774000	1.131431000
C	3.011778000	-0.662620000	0.353210000
C	2.953282000	-0.574957000	-1.032904000
C	1.960320000	0.195196000	-1.631195000
H	0.267764000	1.462855000	-1.340112000
H	3.782799000	-1.256901000	0.828155000
H	1.912272000	0.272014000	-2.710616000
H	3.677465000	-1.099701000	-1.643420000
H	2.127034000	-0.061814000	2.212472000
H	0.280441000	1.354966000	2.470207000
C	-2.684300000	0.456427000	0.843234000
F	-3.594778000	1.159240000	0.165193000
C	-1.985242000	-0.556894000	0.073563000
O	-1.245771000	-1.327383000	0.875609000
C	-0.420030000	-2.296846000	0.224106000
H	-1.035189000	-3.003253000	-0.333068000
H	0.282077000	-1.808771000	-0.452631000
H	0.114658000	-2.811637000	1.017468000
O	-2.030659000	-0.661664000	-1.132639000
H	-2.894006000	0.347631000	1.897503000

$CHFCO_2Me$-P

C	-1.238410000	1.753644000	0.897147000
H	-1.183107000	2.076412000	-0.144978000
H	-1.588434000	2.602320000	1.488954000
C	0.078651000	1.294950000	1.420304000
C	1.020854000	0.513887000	-0.747145000
C	1.121705000	0.732496000	0.646949000
C	2.325708000	0.341204000	1.282039000
C	3.358670000	-0.231009000	0.568297000
C	3.236072000	-0.439399000	-0.805738000
C	2.061346000	-0.062173000	-1.452497000
H	0.118814000	0.793964000	-1.277520000
H	4.268237000	-0.520801000	1.080301000
H	1.956856000	-0.223965000	-2.518677000
H	4.045662000	-0.892375000	-1.363617000
H	2.423872000	0.498735000	2.350174000
H	0.238956000	1.372948000	2.489293000
C	-2.343332000	0.694856000	1.003903000
F	-3.542787000	1.286074000	0.651245000
C	-2.106425000	-0.462437000	0.050295000
O	-1.284435000	-1.354160000	0.583889000
C	-0.875907000	-2.427955000	-0.272899000
H	-1.723209000	-3.079211000	-0.485088000
H	-0.471186000	-2.033696000	-1.204076000
H	-0.108801000	-2.969303000	0.273451000
O	-2.580073000	-0.536917000	-1.051419000
H	-2.434623000	0.322974000	2.025215000

CF_3acetal-rad

C	-2.138500000	-0.059379000	-1.297969000
C	-1.443613000	0.760533000	-0.255372000
C	0.326563000	2.046763000	0.183692000
C	-0.873244000	2.933597000	-0.083236000
H	0.513457000	1.912494000	1.249845000
H	1.230496000	2.359573000	-0.330097000
H	-1.038890000	3.690542000	0.677866000
H	-0.842929000	3.384833000	-1.075890000
O	-0.099323000	0.788656000	-0.377183000
O	-1.967175000	1.989286000	-0.043031000
F	-1.633762000	-1.292519000	-1.353651000
F	-3.443397000	-0.150569000	-1.036437000
F	-2.027759000	0.463290000	-2.536460000

CF_3acetal-TS

C	-0.471508000	0.945567000	-1.489270000
H	-0.238491000	0.029816000	-2.020663000
H	-0.044940000	1.854444000	-1.896612000
C	-1.494355000	1.026475000	-0.607252000
C	-2.125029000	-1.407370000	-0.539465000
C	-2.279255000	-0.089536000	-0.086359000
C	-3.228197000	0.155312000	0.914165000
C	-3.992359000	-0.873091000	1.445992000
C	-3.825083000	-2.174264000	0.988868000
C	-2.887965000	-2.433703000	-0.007575000
H	-1.405197000	-1.630273000	-1.317111000
H	-4.718919000	-0.659276000	2.220564000
H	-2.753739000	-3.444598000	-0.373078000

H	-4.419051000	-2.979736000	1.402290000
H	-3.359314000	1.169106000	1.276068000
H	-1.742640000	1.999110000	-0.192750000
C	1.480270000	-0.443104000	0.466347000
C	1.573418000	0.789976000	-0.386270000
C	2.486180000	2.826170000	-0.486395000
C	3.329278000	1.872688000	-1.310292000
H	1.798578000	3.408133000	-1.102209000
H	3.064341000	3.478265000	0.161642000
H	3.533029000	2.225184000	-2.317633000
H	4.255413000	1.596829000	-0.804146000
O	1.730755000	1.922288000	0.339457000
O	2.481921000	0.706032000	-1.390618000
F	2.636925000	-0.713112000	1.105470000
F	0.541585000	-0.313373000	1.402492000
F	1.193421000	-1.516499000	-0.273044000

CF_3acetal-P

C	-0.100435000	0.781309000	-1.236438000
H	-0.143280000	-0.123928000	-1.841316000
H	-0.119197000	1.633179000	-1.923606000
C	-1.230707000	0.889880000	-0.273916000
C	-2.054153000	-1.456935000	-0.442757000
C	-2.136649000	-0.140789000	0.071144000
C	-3.182418000	0.134926000	0.987275000
C	-4.086215000	-0.839199000	1.357940000
C	-3.986850000	-2.129048000	0.835805000
C	-2.964452000	-2.425293000	-0.063138000
H	-1.266201000	-1.717430000	-1.136981000
H	-4.876798000	-0.599514000	2.058764000
H	-2.877663000	-3.425677000	-0.469650000
H	-4.695352000	-2.893239000	1.128896000
H	-3.263369000	1.135661000	1.396426000
H	-1.366299000	1.851061000	0.207803000
C	1.548397000	-0.310312000	0.382093000
C	1.285121000	0.856689000	-0.588353000
C	2.396525000	2.854256000	-0.567672000
C	3.230769000	1.829834000	-1.304385000
H	1.855705000	3.506767000	-1.256950000
H	2.958525000	3.448400000	0.148741000
H	3.633368000	2.188833000	-2.248466000
H	4.030643000	1.428677000	-0.679442000
O	1.471689000	2.032140000	0.151997000
O	2.263862000	0.811859000	-1.588344000
F	2.818630000	-0.310090000	0.798332000
F	0.776708000	-0.251657000	1.469702000
F	1.324530000	-1.488614000	-0.209100000

CF_2CO_2H-rad

C	1.240781000	-4.050735000	-0.352691000
O	1.984036000	-3.296230000	0.234014000
C	0.377128000	-4.968954000	0.326843000
F	0.333875000	-5.034555000	1.621612000
F	-0.440398000	-5.782769000	-0.270275000
O	1.116966000	-4.143731000	-1.681896000
H	1.723186000	-3.499951000	-2.087639000

CF_2CO_2H-TS

C	-2.219484000	0.327322000	-1.359811000
O	-3.126606000	-0.245909000	-1.909505000
C	-2.046954000	0.338722000	0.091722000
C	-0.784998000	-1.573529000	0.444026000
H	-0.872871000	-1.307942000	1.492273000
H	-1.573480000	-2.193823000	0.034404000
C	0.367632000	-1.408275000	-0.240332000
C	1.660575000	-0.135352000	1.502511000
C	1.555033000	-0.693936000	0.221278000
C	2.635501000	-0.552601000	-0.656597000
C	3.784170000	0.123945000	-0.273863000
C	3.874100000	0.674034000	0.998274000
C	2.807745000	0.540121000	1.883383000
H	0.842895000	-0.229394000	2.206550000
H	4.608030000	0.222931000	-0.969649000
H	2.874295000	0.965898000	2.877047000
H	4.768183000	1.204701000	1.300894000
H	2.563100000	-0.980362000	-1.650296000
H	0.426161000	-1.793756000	-1.253999000
F	-3.106343000	0.076396000	0.825820000
F	-1.292717000	1.266125000	0.644086000
O	-1.199438000	0.926546000	-1.974143000
H	-1.306164000	0.806181000	-2.934477000

CF_2CO_2H-P

C	0.885561000	-4.575846000	0.560482000
O	1.229566000	-5.347770000	1.404978000
C	-0.466945000	-4.661368000	-0.165045000
C	-1.498450000	-3.676321000	0.361408000
H	-1.123803000	-2.671885000	0.161239000
H	-2.385337000	-3.835264000	-0.258037000
C	-1.819715000	-3.902246000	1.799275000
C	-1.652393000	-3.532996000	4.205874000
C	-1.263350000	-3.189167000	2.887380000
C	-0.319977000	-2.144613000	2.736923000
C	0.201406000	-1.497240000	3.841587000
C	-0.193722000	-1.855728000	5.128292000
C	-1.126933000	-2.878531000	5.300149000
H	-2.376869000	-4.327649000	4.341906000
H	0.924769000	-0.703295000	3.701572000
H	-1.440769000	-3.161954000	6.297319000
H	0.220568000	-1.344882000	5.988050000
H	0.002132000	-1.838717000	1.749696000
H	-2.514730000	-4.700963000	2.026641000
F	-0.920194000	-5.930718000	-0.041714000
F	-0.264550000	-4.450422000	-1.493065000
O	1.572568000	-3.519686000	0.147816000
H	2.404366000	-3.459167000	0.652829000

$ClCH_2CHCO_2H$-rad

C	1.215144000	-4.038132000	-0.376122000
O	1.810197000	-3.168949000	0.223998000
C	0.428177000	-5.070113000	0.271190000
H	0.354443000	-5.013227000	1.347584000
O	1.230999000	-4.132761000	-1.716154000
H	1.777638000	-3.408097000	-2.063888000
C	-0.326763000	-6.089151000	-0.469962000
H	0.109235000	-6.325947000	-1.435107000
H	-0.483638000	-6.989251000	0.116193000
Cl	-1.993165000	-5.436038000	-0.825242000

$ClCH_2CHCO_2H$-TS

C	-2.000446000	-0.325438000	-1.344927000
O	-1.435698000	0.591568000	-1.902104000
C	-1.948836000	-0.592985000	0.081657000
C	-0.243530000	-2.144955000	0.041872000

H	-0.265171000	-2.154128000	1.125080000
H	-0.898467000	-2.846925000	-0.458316000
C	0.775617000	-1.573754000	-0.648542000
C	2.714538000	-0.095739000	-0.973794000
C	1.823184000	-0.725273000	-0.095932000
C	1.973833000	-0.500858000	1.280434000
C	2.975684000	0.328847000	1.753516000
C	3.850950000	0.951806000	0.867683000
C	3.717703000	0.734745000	-0.498064000
H	2.607322000	-0.263419000	-2.039604000
H	3.078420000	0.492355000	2.819240000
H	4.395784000	1.213683000	-1.193565000
H	4.632314000	1.600967000	1.242536000
H	1.305375000	-0.980477000	1.985030000
H	0.798361000	-1.697132000	-1.727334000
H	-2.651250000	-1.306236000	0.491278000
O	-2.715918000	-1.243001000	-2.021787000
H	-2.672817000	-1.016858000	-2.965980000
C	-1.370626000	0.434702000	0.962349000
H	-1.082182000	0.038552000	1.931094000
H	-0.541576000	0.956665000	0.493605000
Cl	-2.617065000	1.723931000	1.321258000

$ClCH_2CHCO_2H$-P

C	-1.497069000	1.028722000	-0.958202000
O	-1.541809000	1.045969000	-2.159355000
C	-1.661248000	-0.205521000	-0.099397000
C	-0.346164000	-0.516889000	0.630914000
H	-0.135842000	0.282840000	1.347053000
H	-0.499883000	-1.426960000	1.225618000
C	0.792836000	-0.697381000	-0.312930000
C	3.163646000	-0.833257000	-0.915854000
C	2.156317000	-0.649022000	0.062421000
C	2.572483000	-0.425534000	1.395952000
C	3.915877000	-0.386144000	1.721180000
C	4.889853000	-0.565756000	0.741229000
C	4.501131000	-0.790005000	-0.579916000
H	2.865025000	-1.008992000	-1.943245000
H	4.211373000	-0.214407000	2.749305000
H	5.252610000	-0.931685000	-1.347239000
H	5.939888000	-0.533306000	1.002821000
H	1.831590000	-0.288527000	2.173659000
H	0.565321000	-0.932751000	-1.347311000
H	-1.906779000	-1.023611000	-0.777067000
O	-1.272947000	2.126295000	-0.225734000
H	-1.162545000	2.884695000	-0.824811000
C	-2.775474000	-0.029082000	0.915854000
H	-2.935292000	-0.952481000	1.466499000
H	-2.564495000	0.781643000	1.608884000
Cl	-4.331513000	0.371731000	0.112651000

$CHBrCO_2H$-rad

C	1.219746000	-4.062090000	-0.378958000
O	1.943183000	-3.293240000	0.217404000
C	0.351647000	-4.983154000	0.320430000
H	0.349362000	-4.987729000	1.399275000
Br	-0.767948000	-6.163859000	-0.539838000
O	1.157017000	-4.130432000	-1.712529000
H	1.772952000	-3.471868000	-2.076804000

$CHBrCO_2H$-TS

C	-1.115699000	-0.612203000	0.902835000
O	-1.166314000	-1.491816000	0.074397000
C	-1.929037000	0.592882000	0.927616000
C	-0.492005000	1.938669000	-0.273350000
H	-0.636302000	1.300180000	-1.137347000
H	-1.210156000	2.737268000	-0.139209000
C	0.693832000	1.992950000	0.384538000
C	2.974555000	1.290058000	0.973331000
C	1.824183000	1.094433000	0.198635000
C	1.809550000	0.030721000	-0.715491000
C	2.905323000	-0.806652000	-0.839015000
C	4.041050000	-0.600159000	-0.060872000
C	4.072191000	0.453563000	0.844579000
H	2.996259000	2.108744000	1.683826000
H	2.876320000	-1.625841000	-1.547011000
H	4.952554000	0.622431000	1.452442000
H	4.895290000	-1.258087000	-0.161191000
H	0.936159000	-0.146573000	-1.330678000
H	0.819877000	2.746214000	1.156280000
H	-1.964185000	1.198535000	1.820500000
Br	-3.486293000	0.605929000	-0.103398000
O	-0.237589000	-0.616278000	1.920405000
H	0.349244000	-1.383040000	1.803603000

$CHBrCO_2H$-P

C	-0.848577000	-1.162612000	0.579983000
O	-0.249500000	-1.750564000	-0.277693000
C	-1.242612000	0.297418000	0.495212000
C	-0.226043000	1.123258000	-0.278809000
H	-0.089214000	0.691583000	-1.268545000
H	-0.634954000	2.129813000	-0.402555000
C	1.042776000	1.182959000	0.502471000
C	2.525561000	-0.024423000	-1.100559000
C	2.299313000	0.659761000	0.117842000
C	3.404429000	0.820126000	0.991011000
C	4.652904000	0.332962000	0.664155000
C	4.853294000	-0.334815000	-0.543870000
C	3.781140000	-0.506966000	-1.417468000
H	1.709257000	-0.181238000	-1.792471000
H	5.479145000	0.470364000	1.351009000
H	3.930367000	-1.026696000	-2.356191000
H	5.833199000	-0.716299000	-0.801203000
H	3.251114000	1.338512000	1.930765000
H	0.990181000	1.686758000	1.461907000
H	-1.420232000	0.697914000	1.489319000
Br	-3.002045000	0.317660000	-0.388109000
O	-1.257293000	-1.722725000	1.718087000
H	-1.004634000	-2.663526000	1.711633000

CH_2CO_2H-rad

C	1.223049000	-4.057312000	-0.346386000
O	1.989337000	-3.288966000	0.199353000
C	0.339635000	-4.963666000	0.342042000
H	0.350355000	-4.969901000	1.420559000
H	-0.316823000	-5.619971000	-0.208760000
O	1.124178000	-4.136912000	-1.687317000
H	1.747179000	-3.497819000	-2.071571000

CH_2CO_2H-TS

C	-3.191184000	0.600912000	-0.650148000
O	-4.321813000	0.280924000	-0.337007000
C	-2.133018000	-0.307854000	-1.012477000
C	-1.276912000	-0.583804000	1.123949000

H	-1.232382000	0.493305000	1.232539000
H	-2.184649000	-1.066394000	1.463413000
C	-0.162838000	-1.323807000	0.912187000
C	1.383193000	0.549642000	0.278566000
C	1.150782000	-0.806901000	0.545810000
C	2.223895000	-1.699221000	0.436236000
C	3.489883000	-1.254254000	0.087047000
C	3.706963000	0.094294000	-0.168071000
C	2.647717000	0.992215000	-0.072667000
H	0.569933000	1.262907000	0.338180000
H	4.307093000	-1.960791000	0.010415000
H	2.810173000	2.043133000	-0.278242000
H	4.693336000	0.444567000	-0.445204000
H	2.052855000	-2.751829000	0.631705000
H	-0.241442000	-2.405665000	0.962685000
H	-1.224237000	0.082404000	-1.445904000
H	-2.401840000	-1.337948000	-1.186983000
O	-2.797824000	1.894346000	-0.645444000
H	-3.554672000	2.430912000	-0.357926000

CH_2CO_2H-P

C	-3.050819000	-0.522283000	-0.350098000
O	-2.430200000	-1.279486000	-1.050329000
C	-2.603882000	0.847909000	0.075834000
C	-1.136226000	1.106811000	-0.221042000
H	-0.945498000	0.943837000	-1.286703000
H	-0.925556000	2.167782000	-0.039998000
C	-0.227947000	0.257816000	0.600944000
C	1.845126000	0.933593000	-0.576598000
C	1.172827000	0.187307000	0.420448000
C	1.957249000	-0.651122000	1.250018000
C	3.324634000	-0.738511000	1.087824000
C	3.967217000	0.004684000	0.096678000
C	3.216284000	0.838617000	-0.729222000
H	1.277660000	1.587013000	-1.227821000
H	3.902603000	-1.388052000	1.734259000
H	3.709782000	1.417984000	-1.500407000
H	5.040140000	-0.065714000	-0.029586000
H	1.461507000	-1.229550000	2.021594000
H	-0.663857000	-0.333083000	1.400074000
H	-2.822735000	0.963725000	1.139507000
H	-3.243407000	1.560719000	-0.451840000
O	-4.268501000	-0.822383000	0.124318000
H	-4.516814000	-1.701454000	-0.208589000

$CHFCO_2H$-rad

C	1.225259000	-4.055374000	-0.363565000
O	1.972045000	-3.289115000	0.208840000
C	0.360031000	-4.956025000	0.346237000
H	0.309512000	-5.019077000	1.422137000
F	-0.428645000	-5.764036000	-0.333279000
O	1.122994000	-4.148583000	-1.695927000
H	1.738048000	-3.506418000	-2.089794000

$CHFCO_2H$-TS

C	-1.765581000	0.903953000	-0.514678000
O	-1.740869000	1.840559000	0.253012000
C	-2.599453000	-0.269609000	-0.369639000
C	-1.136972000	-1.607704000	0.749027000
H	-1.227617000	-0.921497000	1.583714000
H	-1.887796000	-2.385456000	0.682510000
C	0.020514000	-1.735017000	0.054032000
C	2.292877000	-1.119087000	-0.661005000
C	1.179360000	-0.856537000	0.146911000
C	1.225960000	0.253609000	1.002730000
C	2.343863000	1.070456000	1.037881000
C	3.442072000	0.797514000	0.226984000
C	3.412564000	-0.302473000	-0.621805000
H	2.268650000	-1.974289000	-1.326827000
H	2.361346000	1.925904000	1.701938000
H	4.263043000	-0.523714000	-1.254824000
H	4.314129000	1.438688000	0.257921000
H	0.382267000	0.483342000	1.641596000
H	0.098761000	-2.530339000	-0.681071000
H	-2.832677000	-0.930066000	-1.192022000
F	-3.543362000	-0.187018000	0.568341000
O	-0.974576000	0.801674000	-1.593581000
H	-0.359448000	1.554436000	-1.590084000

$CHFCO_2H$-P

C	-2.591183000	0.759165000	-0.776213000
O	-3.074372000	1.598490000	-0.071047000
C	-2.374350000	-0.691607000	-0.386409000
C	-0.905209000	-1.073787000	-0.408634000
H	-0.836890000	-2.121323000	-0.090238000
H	-0.563792000	-1.049289000	-1.446970000
C	-0.074102000	-0.190153000	0.457484000
C	2.097337000	-0.975806000	-0.446507000
C	1.339811000	-0.158939000	0.425505000
C	2.047861000	0.709685000	1.290715000
C	3.426590000	0.760907000	1.277882000
C	4.154435000	-0.051460000	0.407532000
C	3.478748000	-0.917221000	-0.449086000
H	1.592233000	-1.659210000	-1.117855000
H	3.945394000	1.435343000	1.948374000
H	4.039721000	-1.552634000	-1.123795000
H	5.236129000	-0.010564000	0.399784000
H	1.484209000	1.341044000	1.968400000
H	-0.574486000	0.429395000	1.193943000
H	-2.949610000	-1.324745000	-1.065016000
F	-2.880818000	-0.861779000	0.887864000
O	-2.167078000	0.977947000	-2.021601000
H	-2.306865000	1.915638000	-2.243169000

$CHMeCO_2H$-rad

C	1.231239000	-4.041066000	-0.363910000
O	1.904602000	-3.215178000	0.224557000
C	0.455506000	-5.071158000	0.278948000
H	0.480369000	-5.061432000	1.359215000
O	1.151751000	-4.062136000	-1.710765000
H	1.705180000	-3.339721000	-2.049974000
C	-0.365830000	-6.051862000	-0.460638000
H	0.161837000	-6.432977000	-1.337239000
H	-0.653751000	-6.884265000	0.178732000
H	-1.285206000	-5.581127000	-0.831522000

$CHMeCO_2H$-TS

C	-1.823741000	-0.892164000	0.548454000
O	-1.802417000	-1.820197000	-0.238913000
C	-2.674802000	0.273343000	0.450170000
C	-1.221716000	1.546634000	-0.733558000
H	-1.311693000	0.852303000	-1.561224000
H	-1.965865000	2.331231000	-0.683399000
C	-0.053499000	1.699222000	-0.055984000

C	2.241243000	1.136439000	0.631308000
C	1.115277000	0.836474000	-0.147270000
C	1.165123000	-0.294006000	-0.976743000
C	2.296521000	-1.091969000	-1.015201000
C	3.406394000	-0.781364000	-0.233876000
C	3.373971000	0.338401000	0.588904000
H	2.215983000	2.007397000	1.276493000
H	2.315278000	-1.963347000	-1.658338000
H	4.232842000	0.589511000	1.199173000
H	4.288745000	-1.408152000	-0.266904000
H	0.312159000	-0.554785000	-1.590628000
H	0.023210000	2.509224000	0.663225000
H	-2.708723000	0.918402000	1.317671000
O	-0.997784000	-0.851080000	1.614328000
H	-0.406927000	-1.619844000	1.557749000
C	-3.823446000	0.259032000	-0.492619000
H	-3.562146000	-0.245779000	-1.422791000
H	-4.670987000	-0.278601000	-0.051963000
H	-4.160627000	1.271524000	-0.716184000

CHMeCO$_2$H-P

C	-2.719383000	-0.549654000	0.928102000
O	-3.126144000	-1.664040000	0.722970000
C	-2.479128000	0.510879000	-0.120696000
C	-1.015695000	0.976899000	-0.071952000
H	-0.881478000	1.744188000	-0.843143000
H	-0.822765000	1.480363000	0.881959000
C	-0.037942000	-0.131473000	-0.267089000
C	2.225617000	-1.051418000	-0.495411000
C	1.363374000	0.057644000	-0.321113000
C	1.957134000	1.336900000	-0.204918000
C	3.330733000	1.487373000	-0.260257000
C	4.160032000	0.381437000	-0.432354000
C	3.594775000	-0.889280000	-0.549401000
H	1.789509000	-2.039856000	-0.587273000
H	3.764194000	2.475991000	-0.167652000
H	4.233635000	-1.753806000	-0.683849000
H	5.234423000	0.507047000	-0.474423000
H	1.327867000	2.208149000	-0.069649000
H	-0.407406000	-1.147894000	-0.358707000
H	-3.102661000	1.361729000	0.168020000
O	-2.426521000	-0.116162000	2.163616000
H	-2.602657000	-0.839809000	2.788771000
C	-2.890239000	0.020947000	-1.499208000
H	-2.747997000	0.818456000	-2.229410000
H	-2.292373000	-0.838047000	-1.807516000
H	-3.938347000	-0.277505000	-1.513717000

CCl$_2$CO$_2$H-rad

C	1.235759000	-4.070466000	-0.360816000
O	1.977837000	-3.317996000	0.220958000
C	0.363420000	-5.017627000	0.329049000
Cl	-0.660879000	-6.066212000	-0.499046000
O	1.116640000	-4.135238000	-1.688740000
H	1.720657000	-3.478837000	-2.077833000
Cl	0.382359000	-5.060061000	2.010187000

CCl$_2$CO$_2$H-TS

C	-2.009302000	0.589748000	1.255510000
O	-1.299538000	0.597937000	2.228401000
C	-1.796161000	-0.301295000	0.099302000
C	-0.575209000	1.160342000	-1.125398000
H	-0.556150000	0.420387000	-1.916660000
H	-1.403901000	1.857917000	-1.143148000
C	0.542534000	1.464100000	-0.411957000
C	2.814993000	1.107356000	0.452203000
C	1.798972000	0.733816000	-0.437863000
C	2.045773000	-0.326416000	-1.324341000
C	3.261819000	-0.986121000	-1.311165000
C	4.259742000	-0.605499000	-0.417624000
C	4.032102000	0.444428000	0.463802000
H	2.635294000	1.926531000	1.139119000
H	3.437266000	-1.800648000	-2.002924000
H	4.804675000	0.747215000	1.159631000
H	5.209844000	-1.124923000	-0.412265000
H	1.284686000	-0.633981000	-2.030536000
H	0.499376000	2.297578000	0.282683000
Cl	-0.746911000	-1.625852000	0.368584000
O	-3.023449000	1.437729000	1.065320000
H	-3.068394000	2.029149000	1.836329000
Cl	-3.112030000	-0.607946000	-0.962011000

CCl$_2$CO$_2$H-P

C	-2.022166000	-1.077555000	0.633813000
O	-2.649655000	-1.945905000	0.107006000
C	-1.687794000	0.282872000	-0.008827000
C	-0.243606000	0.704693000	0.217338000
H	-0.102417000	1.679329000	-0.261979000
H	-0.107736000	0.863281000	1.291490000
C	0.726171000	-0.306347000	-0.294169000
C	2.991257000	-1.172558000	-0.638313000
C	2.125417000	-0.171447000	-0.137721000
C	2.709729000	0.943386000	0.506641000
C	4.082388000	1.042558000	0.638877000
C	4.916608000	0.044989000	0.139653000
C	4.359503000	-1.062909000	-0.499509000
H	2.559006000	-2.033516000	-1.135297000
H	4.511227000	1.905208000	1.134194000
H	5.003924000	-1.841103000	-0.889771000
H	5.990660000	0.129245000	0.246716000
H	2.076548000	1.729106000	0.900246000
H	0.354433000	-1.184732000	-0.810513000
Cl	-2.049639000	0.208292000	-1.742565000
O	-1.526936000	-1.134477000	1.864833000
H	-1.773261000	-1.987184000	2.266783000
Cl	-2.788504000	1.463938000	0.764506000

CHPhCO$_2$H-rad

C	1.272269000	-3.909301000	-0.259504000
O	1.828976000	-3.171419000	0.529618000
C	0.518774000	-5.074233000	0.161239000
H	0.519102000	-5.203010000	1.235548000
O	1.326204000	-3.683024000	-1.584355000
H	1.853816000	-2.878422000	-1.722414000
C	-0.194762000	-6.033892000	-0.605172000
C	-0.315486000	-6.036328000	-2.014580000
C	-0.838495000	-7.070946000	0.113320000
C	-1.041745000	-7.023710000	-2.652144000
C	-1.560492000	-8.050904000	-0.534771000
C	-1.666745000	-8.033128000	-1.923785000
H	0.161169000	-5.264446000	-2.598236000
H	-0.754068000	-7.082161000	1.193603000
H	-1.123970000	-7.009778000	-3.731771000
H	-2.044037000	-8.833084000	0.036762000

H	-2.231489000	-8.801992000	-2.435745000

CHPhCO$_2$H-TS

C	-2.907476000	0.180848000	0.452138000
O	-2.621847000	0.574118000	1.562866000
C	-2.063525000	-0.578752000	-0.460631000
C	-1.304272000	1.161612000	-1.583856000
H	-0.875312000	0.476208000	-2.304257000
H	-2.297934000	1.526138000	-1.813790000
C	-0.510210000	1.877521000	-0.731575000
C	1.648521000	0.704238000	-1.222849000
C	0.886975000	1.605189000	-0.459991000
C	1.502940000	2.233090000	0.634015000
C	2.815717000	1.949576000	0.973690000
C	3.548412000	1.037884000	0.221490000
C	2.959740000	0.424760000	-0.882560000
H	1.211031000	0.220440000	-2.087083000
H	3.269063000	2.435864000	1.828625000
H	3.530536000	-0.277243000	-1.478342000
H	4.573559000	0.810924000	0.486543000
H	0.926989000	2.934449000	1.227317000
H	-0.969223000	2.651860000	-0.124783000
H	-2.575497000	-0.957362000	-1.336424000
O	-4.106412000	0.465712000	-0.101918000
H	-4.601036000	1.005137000	0.536436000
C	-0.875861000	-1.299344000	-0.055229000
C	-0.173247000	-1.048499000	1.135442000
C	-0.363034000	-2.267103000	-0.936615000
C	0.986608000	-1.750146000	1.427744000
C	0.797377000	-2.960376000	-0.641136000
C	1.480021000	-2.704462000	0.544875000
H	-0.542569000	-0.302179000	1.823126000
H	-0.891417000	-2.464930000	-1.862729000
H	1.515166000	-1.544083000	2.350822000
H	1.173542000	-3.701597000	-1.335547000
H	2.389053000	-3.244908000	0.777859000

CHPhCO$_2$H-P

C	-2.900246000	0.741287000	0.358801000
O	-2.966300000	1.168964000	1.481052000
C	-1.623632000	0.464002000	-0.404757000
C	-0.424721000	1.100132000	0.298215000
H	-0.625978000	2.167734000	0.451201000
H	-0.331260000	0.673898000	1.302660000
C	0.838341000	0.913979000	-0.468431000
C	3.273813000	1.025956000	-0.751589000
C	2.117272000	1.251760000	0.033063000
C	2.298155000	1.818215000	1.317409000
C	3.562239000	2.134097000	1.780613000
C	4.686587000	1.899666000	0.992516000
C	4.530450000	1.343330000	-0.278086000
H	3.155878000	0.592684000	-1.738570000
H	3.677177000	2.568687000	2.766458000
H	5.400250000	1.156981000	-0.896530000
H	5.673473000	2.148507000	1.361285000
H	1.435657000	2.012130000	1.943123000
H	0.780193000	0.520700000	-1.477573000
H	-1.746595000	0.907781000	-1.395816000
O	-3.986670000	0.436827000	-0.361423000
H	-4.775378000	0.607068000	0.182207000
C	-1.460295000	-1.035792000	-0.576863000
C	-1.367648000	-1.600694000	-1.844634000
C	-1.378599000	-1.863994000	0.541709000
C	-1.190432000	-2.970997000	-1.995317000
C	-1.206434000	-3.232866000	0.392953000
C	-1.110115000	-3.790101000	-0.877309000
H	-1.429185000	-0.961954000	-2.718405000
H	-1.450898000	-1.435718000	1.536194000
H	-1.114382000	-3.397029000	-2.988165000
H	-1.148083000	-3.865926000	1.269728000
H	-0.972943000	-4.857964000	-0.993998000

CHClCO$_2$H-rad

C	1.211146000	-4.071517000	-0.380622000
O	1.939222000	-3.306752000	0.215446000
C	0.340339000	-4.992042000	0.314054000
H	0.328838000	-5.005546000	1.392768000
Cl	-0.674421000	-6.063608000	-0.492590000
O	1.143835000	-4.137145000	-1.715310000
H	1.763277000	-3.481747000	-2.079224000

CHClCO$_2$H-TS

C	-2.604085000	1.065878000	-0.813205000
O	-3.744877000	0.697810000	-0.657273000
C	-1.434099000	0.208399000	-0.917870000
C	-0.747236000	0.504339000	1.253651000
H	-0.495395000	1.527035000	1.001196000
H	-1.735571000	0.337408000	1.665681000
C	0.203541000	-0.451945000	1.400236000
C	2.115689000	0.801308000	0.366257000
C	1.595271000	-0.344256000	0.985199000
C	2.450056000	-1.434107000	1.194652000
C	3.780228000	-1.381841000	0.808287000
C	4.282695000	-0.238464000	0.199247000
C	3.444156000	0.850732000	-0.020944000
H	1.478674000	1.658405000	0.185001000
H	4.425794000	-2.234007000	0.981381000
H	3.831125000	1.742710000	-0.497964000
H	5.320936000	-0.195049000	-0.105426000
H	2.055422000	-2.327114000	1.666093000
H	-0.091129000	-1.417910000	1.798309000
H	-0.512180000	0.593920000	-1.328394000
Cl	-1.704314000	-1.458351000	-1.161752000
O	-2.253950000	2.364229000	-0.862081000
H	-3.057763000	2.895862000	-0.734520000

CHClCO$_2$H-P

C	-2.933582000	-1.148317000	0.354052000
O	-3.792164000	-1.353170000	1.167472000
C	-1.882888000	-0.066145000	0.498400000
C	-0.473627000	-0.548603000	0.212098000
H	-0.307180000	-1.444119000	0.829175000
H	-0.409708000	-0.880090000	-0.827608000
C	0.550600000	0.488598000	0.515662000
C	2.816308000	1.418945000	0.474692000
C	1.907925000	0.388313000	0.131811000
C	2.414278000	-0.717923000	-0.590393000
C	3.748911000	-0.779069000	-0.946894000
C	4.624116000	0.248421000	-0.602005000
C	4.145772000	1.346999000	0.112768000
H	2.448015000	2.274166000	1.030192000
H	4.114914000	-1.636211000	-1.499326000
H	4.820396000	2.149225000	0.386111000
H	5.667576000	0.194369000	-0.885336000

H	1.752359000	-1.529796000	-0.864976000
H	0.251021000	1.350303000	1.101642000
H	-1.970496000	0.347810000	1.499525000
Cl	-2.346493000	1.259639000	-0.629863000
O	-2.798960000	-1.843900000	-0.773521000
H	-3.508804000	-2.508284000	-0.818772000

cbutCO$_2$H-rad

C	1.093930000	-4.008167000	-0.292831000
O	1.802252000	-3.220682000	0.308908000
O	0.866428000	-3.901277000	-1.619805000
H	1.355285000	-3.126598000	-1.942822000
C	-0.573354000	-6.728817000	1.244003000
C	-0.482315000	-6.189317000	-0.211696000
C	0.424055000	-5.123099000	0.305864000
C	0.393457000	-5.605052000	1.715626000
H	-0.185401000	-7.736137000	1.373645000
H	-1.568628000	-6.665491000	1.677101000
H	-1.418728000	-5.823944000	-0.641062000
H	-0.020760000	-6.866633000	-0.935149000
H	-0.019916000	-4.892703000	2.434041000
H	1.358706000	-5.948576000	2.096890000

cbutCO$_2$H-TS

C	-2.503358000	1.656153000	-0.523059000
O	-2.173772000	2.429765000	-1.401358000
C	-0.366453000	1.088214000	1.131045000
H	-0.015733000	1.827965000	0.420200000
H	-1.157056000	1.397583000	1.803910000
C	0.394301000	0.009518000	1.453601000
C	2.167321000	-1.661821000	1.119698000
C	1.611399000	-0.421477000	0.778927000
C	2.261627000	0.349047000	-0.197187000
C	3.415935000	-0.112096000	-0.807542000
C	3.951664000	-1.349695000	-0.461626000
C	3.322014000	-2.122683000	0.506288000
H	1.676878000	-2.265077000	1.875678000
H	3.904763000	0.497331000	-1.557798000
H	3.731713000	-3.085938000	0.784563000
H	4.854845000	-1.705280000	-0.941593000
H	1.863077000	1.316553000	-0.477526000
H	0.054378000	-0.640918000	2.254271000
O	-3.440623000	1.975883000	0.394878000
H	-3.745945000	2.878438000	0.206759000
C	-1.571353000	-1.734062000	-0.468542000
C	-2.529195000	-0.854890000	0.378707000
C	-1.935447000	0.345533000	-0.311516000
C	-1.186422000	-0.489444000	-1.311231000
H	-2.037191000	-2.551036000	-1.015001000
H	-0.734104000	-2.119986000	0.111433000
H	-2.420934000	-0.907957000	1.462988000
H	-3.583753000	-0.995183000	0.126995000
H	-0.120737000	-0.286028000	-1.428924000
H	-1.655544000	-0.467230000	-2.298728000

cbutCO$_2$H-P

C	-2.095388000	0.970752000	-1.262052000
O	-2.027736000	0.509103000	-2.372083000
C	-0.661444000	0.882325000	0.782903000
H	-0.959311000	1.902243000	1.044264000
H	-0.531960000	0.335359000	1.717523000
C	0.593051000	0.916787000	-0.017402000
C	2.855375000	0.277659000	-0.683069000
C	1.743026000	0.117787000	0.182003000
C	1.855408000	-0.851615000	1.209242000
C	3.008549000	-1.599394000	1.355889000
C	4.089475000	-1.420741000	0.494437000
C	4.000508000	-0.475043000	-0.527423000
H	2.790673000	1.011487000	-1.478636000
H	3.069843000	-2.334261000	2.149789000
H	4.833679000	-0.330071000	-1.204442000
H	4.989088000	-2.010393000	0.616842000
H	1.029787000	-1.015597000	1.889839000
H	0.627259000	1.618451000	-0.845669000
O	-2.441346000	2.247979000	-1.038619000
H	-2.608332000	2.676430000	-1.895519000
C	-2.603106000	-1.433159000	1.149026000
C	-3.071603000	0.020948000	0.926288000
C	-1.830058000	0.229953000	0.017692000
C	-1.700042000	-1.300047000	-0.096937000
H	-3.369088000	-2.205558000	1.122642000
H	-2.024076000	-1.535561000	2.066379000
H	-3.145716000	0.678443000	1.792353000
H	-3.998361000	0.065766000	0.353108000
H	-0.689949000	-1.706247000	-0.042901000
H	-2.191155000	-1.666868000	-0.997298000

CH^{i}PrCO$_2$H-rad

C	1.054333000	-3.791627000	-0.305037000
O	1.811145000	-3.036962000	0.277999000
C	0.817291000	-5.161235000	0.076988000
H	1.391281000	-5.509835000	0.927024000
O	0.353182000	-3.392804000	-1.386848000
H	0.579726000	-2.463709000	-1.556288000
C	-0.065200000	-6.103310000	-0.658164000
H	-0.877713000	-5.535718000	-1.118091000
C	0.734550000	-6.775581000	-1.786841000
H	1.564801000	-7.351578000	-1.371697000
H	0.088188000	-7.455361000	-2.345436000
H	1.139336000	-6.036749000	-2.480111000
C	-0.646699000	-7.149976000	0.288558000
H	-1.229203000	-6.683552000	1.084845000
H	-1.297124000	-7.836295000	-0.255888000
H	0.154586000	-7.732911000	0.749371000

CH^{i}PrCO$_2$H-TS

C	-2.029804000	-1.625474000	1.025440000
O	-2.989018000	-2.221708000	0.575784000
C	-1.552541000	-0.331399000	0.578943000
C	-0.141648000	-1.310577000	-0.906294000
H	0.257503000	-1.900346000	-0.090052000
H	-0.957646000	-1.751357000	-1.465933000
C	0.600948000	-0.324457000	-1.476778000
C	2.262562000	-0.065975000	0.380349000
C	1.789963000	0.281140000	-0.893568000
C	2.483113000	1.263143000	-1.613356000
C	3.617213000	1.864677000	-1.089414000
C	4.079522000	1.502234000	0.170124000
C	3.394916000	0.536555000	0.902237000
H	1.739053000	-0.809667000	0.968759000
H	4.140399000	2.619913000	-1.662910000
H	3.745345000	0.254836000	1.887580000
H	4.964202000	1.971066000	0.582427000
H	2.121535000	1.549302000	-2.594848000

H	0.268052000	0.101296000	-2.417987000
H	-0.770860000	0.122592000	1.178855000
O	-1.255449000	-2.168827000	1.992032000
H	-1.612095000	-3.049823000	2.191436000
C	-2.471163000	0.566279000	-0.192002000
H	-2.804524000	0.032120000	-1.087707000
C	-3.715355000	0.892123000	0.649870000
H	-4.257121000	-0.011263000	0.928436000
H	-3.427481000	1.421695000	1.561645000
H	-4.388067000	1.537514000	0.080996000
C	-1.779046000	1.863351000	-0.596544000
H	-0.901592000	1.685615000	-1.217765000
H	-2.465981000	2.500953000	-1.155742000
H	-1.458517000	2.412749000	0.292693000

CH^{i}PrCO$_2$H-P

C	-1.732969000	1.237099000	-0.932123000
O	-1.488123000	2.227452000	-0.291019000
C	-1.874515000	-0.149293000	-0.345771000
C	-0.580724000	-0.492681000	0.398465000
H	-0.482909000	0.172825000	1.263632000
H	-0.663699000	-1.507623000	0.802782000
C	0.622550000	-0.397084000	-0.476694000
C	3.032125000	-0.323531000	-0.921936000
C	1.956711000	-0.429145000	-0.006939000
C	2.275534000	-0.567073000	1.364716000
C	3.592514000	-0.592670000	1.786095000
C	4.634730000	-0.481658000	0.868211000
C	4.342143000	-0.347665000	-0.489718000
H	2.808376000	-0.219313000	-1.977746000
H	3.813576000	-0.700971000	2.841221000
H	5.146770000	-0.261420000	-1.210034000
H	5.663175000	-0.500586000	1.205688000
H	1.478851000	-0.658272000	2.092738000
H	0.474023000	-0.336333000	-1.550446000
H	-2.017220000	-0.854932000	-1.168374000
O	-1.914530000	1.273787000	-2.258635000
H	-1.823334000	2.197503000	-2.552837000
C	-3.126029000	-0.181496000	0.552835000
H	-2.975491000	0.559745000	1.344380000
C	-4.369100000	0.202655000	-0.245596000
H	-4.289824000	1.204222000	-0.675273000
H	-4.530574000	-0.504642000	-1.063979000
H	-5.254130000	0.187872000	0.392532000
C	-3.313471000	-1.554108000	1.190120000
H	-4.256075000	-1.586288000	1.739676000
H	-3.345469000	-2.332098000	0.421651000
H	-2.514772000	-1.798740000	1.890427000

CMe$_2$CO$_2$H-rad

C	1.259968000	-4.060760000	-0.345224000
O	1.976633000	-3.239373000	0.200508000
C	0.482671000	-5.072598000	0.330696000
O	1.129913000	-4.090095000	-1.691280000
H	1.684996000	-3.379727000	-2.050947000
C	-0.358384000	-6.025005000	-0.439246000
H	0.151498000	-6.384989000	-1.333740000
H	-0.641453000	-6.875631000	0.180500000
H	-1.281140000	-5.537055000	-0.776025000
C	0.485977000	-5.116843000	1.811429000
H	-0.535328000	-5.012423000	2.193535000
H	0.844848000	-6.090659000	2.160952000
H	1.108019000	-4.334327000	2.239494000

CMe$_2$CO$_2$H-TS

C	-2.206718000	0.206786000	-1.258738000
O	-2.942341000	-0.502499000	-1.918503000
C	-2.216614000	0.315097000	0.194094000
C	-0.855011000	-1.428709000	0.505996000
H	-0.877481000	-1.198966000	1.565254000
H	-1.626972000	-2.097872000	0.146130000
C	0.285733000	-1.281593000	-0.224563000
C	1.689508000	-0.103100000	1.494285000
C	1.496111000	-0.590129000	0.191969000
C	2.521680000	-0.395758000	-0.743933000
C	3.690303000	0.266036000	-0.399578000
C	3.863275000	0.747837000	0.892358000
C	2.857012000	0.557411000	1.836247000
H	0.920851000	-0.243657000	2.244208000
H	4.467197000	0.406866000	-1.141004000
H	2.986875000	0.927303000	2.845974000
H	4.773678000	1.266907000	1.164456000
H	2.387594000	-0.769871000	-1.752822000
H	0.292768000	-1.653637000	-1.244827000
O	-1.245675000	0.949701000	-1.846122000
H	-1.279359000	0.764666000	-2.798818000
C	-1.457655000	1.422418000	0.847650000
H	-0.463225000	1.547035000	0.421572000
H	-1.992095000	2.372168000	0.722422000
H	-1.366694000	1.237917000	1.919488000
C	-3.425396000	-0.206418000	0.897634000
H	-3.185229000	-0.465649000	1.930581000
H	-4.200979000	0.568454000	0.929090000
H	-3.842295000	-1.077175000	0.394445000

CMe$_2$CO$_2$H-P

C	-2.337609000	-0.504013000	-1.054945000
O	-2.286637000	0.012326000	-2.141110000
C	-2.001130000	0.169166000	0.263487000
C	-0.900352000	-0.661376000	0.979440000
H	-0.650643000	-0.127588000	1.897736000
H	-1.340205000	-1.619078000	1.269781000
C	0.305212000	-0.915855000	0.143694000
C	2.613359000	-0.627272000	-0.616619000
C	1.547254000	-0.243353000	0.235142000
C	1.792713000	0.813073000	1.145722000
C	3.025567000	1.436396000	1.193827000
C	4.058561000	1.039989000	0.346856000
C	3.839887000	0.002112000	-0.559540000
H	2.448356000	-1.433268000	-1.322718000
H	3.187234000	2.242316000	1.899509000
H	4.636568000	-0.313406000	-1.222403000
H	5.021125000	1.533287000	0.391712000
H	1.007294000	1.144315000	1.812760000
H	0.225435000	-1.693033000	-0.610259000
O	-2.717168000	-1.781449000	-0.895484000
H	-2.917200000	-2.151237000	-1.772035000
C	-1.521728000	1.589682000	-0.001546000
H	-0.625265000	1.602160000	-0.622653000
H	-2.293815000	2.168937000	-0.509298000
H	-1.293631000	2.076914000	0.948286000
C	-3.260923000	0.189852000	1.136133000
H	-3.610679000	-0.819378000	1.353888000
H	-3.033381000	0.688734000	2.079937000

H	-4.065697000	0.740216000	0.644285000

1.6.3. Decarbonylation *vs* radical addition

CF_3CO-decarbonylation-TS (MeCN)

C	-0.365480000	0.164283000	0.105792000
F	0.047981000	-1.002026000	-0.333444000
F	0.253998000	0.500357000	1.210838000
F	-0.229722000	1.090557000	-0.812017000
C	-2.486885000	0.064076000	0.574224000
O	-2.975791000	-0.936814000	0.384731000

Styrene (EtOAc)

C	-1.440906000	-3.392879000	0.550852000
H	-0.462312000	-2.949496000	0.408127000
H	-1.951193000	-3.742968000	-0.337556000
C	-2.007821000	-3.511036000	1.747345000
C	-2.191266000	-3.265445000	4.197944000
C	-1.439593000	-3.081675000	3.036886000
C	-0.175032000	-2.495640000	3.150295000
C	0.314496000	-2.106174000	4.385597000
C	-0.446866000	-2.292793000	5.535156000
C	-1.702500000	-2.874616000	5.437368000
H	-3.173063000	-3.719447000	4.122190000
H	1.296444000	-1.654524000	4.456025000
H	-2.302973000	-3.025979000	6.325906000
H	-0.061572000	-1.984974000	6.499141000
H	0.432409000	-2.342182000	2.267004000
H	-2.993392000	-3.963645000	1.815375000

CF_3CO-rad (EtOAc)

C	0.278435000	-5.812598000	-1.150095000
F	-0.886751000	-6.423681000	-1.284965000
C	0.243000000	-4.892839000	0.110277000
O	1.088016000	-4.823238000	0.905619000
F	1.234818000	-6.727582000	-1.073227000
F	0.497053000	-5.050320000	-2.212349000

CF_3CO-styrene-TS (EtOAc)

C	-2.273679000	-0.336599000	-0.134593000
F	-3.299191000	-0.592532000	-0.945568000
C	-1.544277000	0.925361000	-0.667600000
O	-0.913926000	0.912800000	-1.660115000
F	-2.750214000	-0.150251000	1.088828000
F	-1.481688000	-1.406960000	-0.114280000
C	-0.448092000	1.815132000	1.158438000
H	-0.078613000	2.605993000	0.517497000
H	-1.318797000	2.047067000	1.757889000
C	0.242565000	0.677244000	1.371108000
C	1.985609000	-1.004885000	0.944161000
C	1.461979000	0.264634000	0.680411000
C	2.114598000	1.079219000	-0.251045000
C	3.257330000	0.633744000	-0.894259000
C	3.768704000	-0.630355000	-0.621215000
C	3.129547000	-1.449435000	0.300538000
H	1.481538000	-1.643999000	1.660389000
H	3.753327000	1.274855000	-1.612236000
H	3.521783000	-2.435514000	0.515697000
H	4.662666000	-0.974253000	-1.126362000
H	1.733629000	2.068992000	-0.468587000
H	-0.150497000	-0.039512000	2.086624000

CF_3CO-decarbonylation-TS (EtOAc)

C	-0.365480000	0.164283000	0.105792000
F	0.047981000	-1.002026000	-0.333444000
F	0.253998000	0.500357000	1.210838000
F	-0.229722000	1.090557000	-0.812017000
C	-2.486885000	0.064076000	0.574224000
O	-2.975791000	-0.936814000	0.384731000

2. References

[1] M. J. Frisch, G. W. Trucks, H. B. Schlegel, G. E. Scuseria, M. A. Robb, J. R. Cheeseman, G. Scalmani, V. Barone, B. Mennucci, G. A. Petersson, H. Nakatsuji, M. Caricato, X. Li, H. P. Hratchian, A. F. Izmaylov, J. Bloino, G. Zheng, J. L. Sonnenberg, M. Hada, M. Ehara, K. Toyota, R. Fukuda, J. Hasegawa, M. Ishida, T. Nakajima, Y. Honda, O. Kitao, H. Nakai, T. Vreven, J. J. A. Montgomery, J. E. Peralta, F. Ogliaro, M. Bearpark, J. J. Heyd, E. Brothers, K. N. Kudin, V. N. Staroverov, T. Keith, R. Kobayashi, J. Normand, K. Raghavachari, A. Rendell, J. C. Burant, S. S. Iyengar, J. Tomasi, M. Cossi, N. Rega, J. M. Millam, M. Klene, J. E. Knox, J. B. Cross, V. Bakken, C. Adamo, J. Jaramillo, R. Gomperts, R. E. Stratmann, O. Yazyev, A. J. Austin, R. Cammi, C. Pomelli, J. W. Ochterski, R. L. Martin, K. Morokuma, V. G. Zakrzewski, G. A. Voth, P. Salvador, J. J. Dannenberg, S. Dapprich, A. D. Daniels, O. Farkas, J. B. Foresman, J. V. Ortiz, J. Cioslowski, D. J. Fox, **2013**, *Wallingford, CT.*

[2] a) P. Pracht, F. Bohle, S. Grimme, *Phys. Chem. Chem. Phys.* **2020**, *22*, 7169-7192; b) S. Grimme, *J. Chem. Theory Comput.* **2019**, *15*, 2847-2862.

[3] F. De Vleeschouwer, V. Van Speybroeck, M. Waroquier, P. Geerlings, F. De Proft, *Org. Lett.* **2007**, *9*, 2721-2724.

[4] a) P. C. St. John, Y. Guan, Y. Kim, B. D. Etz, S. Kim, R. S. Paton, *Sci. Data* **2020**, *7*, 244; b) S. R. Jensen, S. Saha, J. A. Flores-Livas, W. Huhn, V. Blum, S. Goedecker, L. Frediani, *J. Phys. Chem. Lett.* **2017**, *8*, 1449-1457; c) F. Jensen, *J. Phys. Chem. A* **2017**, *121*, 6104-6107; d) Y. Zhao, D. G. Truhlar, *Theor. Chem. Account.* **2008**, *120*, 215-241.

[5] a) F. Neese, *WIREs Comput. Mol. Sci.* **2022**, *12*, e1606; b) C. Riplinger, F. Neese, *J. Chem. Phys.* **2013**, *138*, 034106.

[6] a) G. Luchini, J. V. Alegre-Requena, I. Funes-Ardoiz, R. S. Paton, *F1000Research* **2020**, *9*, 291; b) Y.-P. Li, J. Gomes, S. Mallikarjun Sharada, A. T. Bell, M. Head-Gordon, *J. Phys. Chem. C* **2015**, *119*, 1840-1850; c) S. Grimme, *Chem. Eur. J.* **2012**, *18*, 9955-9964.

[7] a) E. Cancès, B. Mennucci, J. Tomasi, *J. Chem. Phys.* **1997**, *107*, 3032-3041; b) A. V. Marenich, C. J. Cramer, D. G. Truhlar, *J. Phys. Chem. B* **2009**, *113*, 6378-6396.

[8] a) A. J. Fernandes, R. Giri, K. N. Houk, D. Katayev, *Angew. Chem. Int. Ed.* **2024**, *63*, e202318377; b) R. Giri, E. Zhilin, A. J. Fernandes, Q. E. L. Ordan, M. Kissling, D. Katayev, *Helv. Chim. Acta* **2024**, *107*, e202400125; c) R. Giri, E. Zhilin, D. Katayev, *Chem. Sci.* **2024**, *15*, 10659-10667.
